# Taxol and β-tubulins from endophytic fungi isolated from the Himalayan Yew, *Taxus wallichiana* Zucc.

**DOI:** 10.3389/fmicb.2022.956855

**Published:** 2022-09-29

**Authors:** Heriberto Vélëz, Dhurva Prasad Gauchan, María del Rosario García-Gil

**Affiliations:** ^1^Department of Forest Mycology and Plant Pathology, Swedish University of Agricultural Sciences, Uppsala, Sweden; ^2^Department of Biotechnology, School of Science, Kathmandu University, Dhulikhel, Nepal; ^3^Department of Forest Genetics and Plant Physiology, Umeå Plant Science Centre, Swedish University of Agricultural Sciences, Umeå, Sweden

**Keywords:** *Taxomyces andreanae*, fungal endophytes, β-tubulin, paclitaxel, Taxol^®^

## Abstract

Paclitaxel, better known as the anticancer drug Taxol^®^, has been isolated from several plant species and has been shown to be produced by fungi, actinomycetes, and even bacteria isolated from marine macroalgae. Given its cytostatic effect, studies conducted in the 1990's showed that paclitaxel was toxic to many pathogenic fungi and oomycetes. Further studies led to the idea that the differences in paclitaxel sensitivity exhibited by different fungi were due to differences in the β-tubulin protein sequence. With the recent isolation of endophytic fungi from the leaves and bark of the Himalayan Yew, *Taxus wallichiana* Zucc., and the availability of genomes from paclitaxel-producing fungi, we decided to further explore the idea that endophytic fungi isolated from Yews should be well-adapted to their environment by encoding β-tubulin proteins that are insensitive to paclitaxel. Our results found evidence of episodic positive/diversifying selection at 10 sites (default *p*-value threshold of 0.1) in the β-tubulin sequences, corresponding to codon positions 33, 55, 172, 218, 279, 335, 359, 362, 379, and 406. Four of these positions (i.e., 172, 279, 359, and 362) have been implicated in the binding of paclitaxel by β-tubulin or formed part of the binding pocket. As expected, all the fungal endophytes grew in different media regardless of the paclitaxel concentration tested. Furthermore, our results also showed that *Taxomyces andreanae* CBS 279.92, the first fungus shown to produce paclitaxel, is a Basidiomycete fungus as the two beta tubulins encoded by the fungus clustered together with other Basidiomycete fungi.

## Introduction

**Figure 1:**
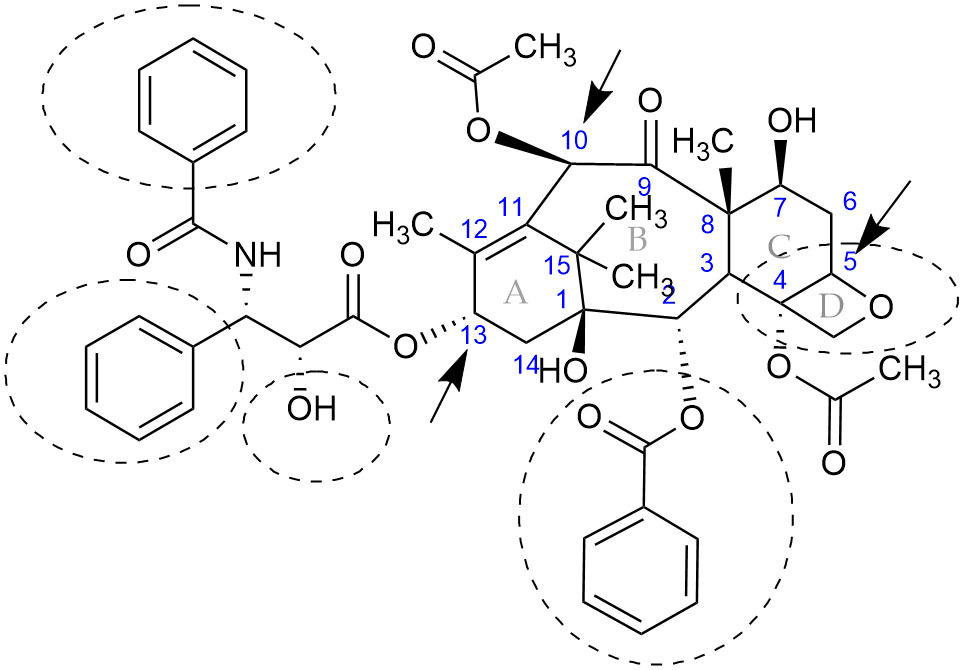
Chemical structure of the anticancer compound, paclitaxel, better known by its registered name, Taxol^®^. The central rings are specified with the letters A, B, C, and D, while the carbons are numbered in blue according to (Nicolaou et al., 1994). The compound is produced by *Taxus* spp., some fungi, actinomycetes, and, most recently, bacteria isolated from marine macroalgae. Some fungal endophytes could modify the functional groups at C-10 and C-13 (indicated by arrows at rings B and A, respectively), or modify the oxetane (ring D) and convert the paclitaxel into less active metabolites (Kingston, 1994; Hu et al., 1996; Zhang et al., 1998; Baloglu and Kingston, 1999). Functional groups that are known to be required for activity are encircled with dashed lines (Kingston, 1994).

The anticancer compound, paclitaxel (Taxol^®^ Figure 1), has been isolated from several plants, including yews (*Taxus* species), bald cypress (*Taxodium distichum* L.), plum-yew (*Cephalotaxus mannii, C. fortunei, C. hainanensis*), yew-pine (*Podocarpus forrestii*), and hazel (*Corylus avellana* L.) (Baloglu and Kingston, 1999; Zhou et al., 2010; Wang et al., 2011; Hao et al., 2013; Gond et al., 2014; Kusari et al., 2014). Likewise, there are many reports of fungi that can produce paclitaxel that were isolated as endophytes from plants that also produce paclitaxel and from plants that do not produce paclitaxel (e.g., *Wollemia nobilis, Ginkgo biloba*, and *Corchorus olitorius* L.) (Flores-Bustamante et al., 2010; Zhou et al., 2010; Hao et al., 2013; Gond et al., 2014; Kusari et al., 2014; Das et al., 2017). In *Taxus* spp., production of paclitaxel requires about 19 enzymatic steps, but very little is known about the biosynthetic machinery responsible for paclitaxel production in fungi (Gond et al., 2014; Kusari et al., 2014). In addition, paclitaxel has been shown to be produced by actinomycetes and, most recently, bacteria isolated from marine macroalgae; however, the biosynthetic pathway also remains elusive in these organisms (Caruso et al., 2000; Subramanian and Marudhamuthu, 2020). Interestingly, fungi that have been isolated from *Taxus* spp. as endophytes known to produce paclitaxel (Flores-Bustamante et al., 2010; Zhou et al., 2010; Hao et al., 2013; Gond et al., 2014; Kusari et al., 2014) also cause diseases on *Taxus* spp. (Mirski, 2012). Though it is known that certain endophytes can change their lifestyle to a pathogenic state (Khare et al., 2018), the role of paclitaxel in these cases has not been explored. However, it has been reported that an endophyte produces paclitaxel *in planta* to ward off wood-decaying fungi, thereby protecting its niche and assisting in plant defense (Soliman et al., 2013, 2015).

Initial studies, with paclitaxel and cancer cells, showed that paclitaxel disrupted the tubulin-microtubule equilibrium during mitosis, by stabilizing microtubules against depolymerization and thereby blocking cells in the G2 and M phase of the cell cycle (Wall and Wani, 1994). Paclitaxel and some of its analogs (e.g., cephalomannine) were also found to be fungi-toxic to many pathogenic fungi, but in particular toward oomycetes (Young et al., 1992; Elmer et al., 1994; Mu et al., 1999). Studies with the paclitaxel producer, *Pestalotiopsis microspora* Ne32, and the paclitaxel-sensitive oomycete, *Pythium ultimum*, established the importance of certain amino acids in the biding between paclitaxel and the β-tubulin protein (Mu et al., 1999). In fact, the epiphytic yeast, *Saccharomyces cerevisiae*, is not sensitive to paclitaxel (Gupta et al., 2003). Thus, Gupta et al. mutated the β-tubulin gene in *S. cerevisiae* resulting in five amino acid changes to study the differences in binding affinity with paclitaxel and epothilone (Gupta et al., 2003). Though the mutated β-tubulin protein could now bind paclitaxel, it remained insensitive to the compound. Thus, the mutated the β-tubulin gene was introduced in a *S. cerevisiae* mutant strain (AD1-8) lacking seven ABC transporters and a transporter transcription factor to create the first paclitaxel-sensitive yeast strain (Foland et al., 2005). Accordingly and as suggested by researchers, the differences in paclitaxel sensitivity exhibited by different fungi were due to differences in target sites or the different mechanism for detoxification (Elmer et al., 1994; Mu et al., 1999).

With the recent isolation of endophytic fungi from the leaves and bark of the Himalayan Yew, *Taxus wallichiana* Zucc. (Gauchan et al., 2021), we decided to further explore the idea that endophytic fungi isolated from *Taxus* spp. should be well-adapted to their environment by encoding β-tubulin proteins that are insensitive to paclitaxel.

## Materials and methods

### Strains and media

Fungi used in this study were previously isolated from *T. wallichiana* [Table 1; (Gauchan et al., 2021)] and were maintained in malt extract agar (MEA; Duchefa Biochemie) at 20°C. Solid Modified Melin Norkrans medium (MMN; Marx 1969) and potato dextrose agar (PDA; Thermo Fisher) were used for the growth assays. Liquid MMN media were used to grow the fungi for genomic DNA (gDNA) and RNA extraction.

**Table 1:** Fungi isolated from the Himalayan Yew (*Taxus wallichiana Zucc*.) and used in this study.

**Sample code**	**Division**	**Fungus name**	**β-tubulin accession no**.	**ITS accession no**.
P4*§	B	*Bjerkandera adusta* strain PAR4 (monokaryon)	ON960039	ON508859
P5§	A	*Diaporthe* sp. strain PAR5	OM674446	ON508862
P7§	B	*Bjerkandera adusta* strain PAR7	OM674444	ON508860
P10A	A	*Hyphopichia* sp. strain PAR10A	OM674451	ON508865
P10B	A	*Aspergillus tubingensis* strain PAR10B	OM674443	ON508858
P14	A	*Kurtzmaniella quercitrusa* strain PAR14	OM674445	ON508861
P15§	A	*Alternaria arborescens* strain PAR15	OM674441	ON508857
M1§	A	*Annulohypoxylon* sp. strain MUS1	OM674442	MN699475
M2	A	*Meyerozyma guilliermondii* strain MUS2	ON960040	ON508869
M9	A	*Trichoderma atroviride* strain MUS9	OM674454	ON508868
M16	A	*Trichoderma atroviride* strain MUS16	OM674453	ON508867
S5	A	*Penicillium* sp. strain SOL5	OM674452	ON508866
S2	A	*Fusarium* sp. strain SOL2	OM674449	ON508864
			OM674450	
S6	A	*Fusarium* sp. strain SOL6	OM674447	ON508863
			OM674448	

^*^Mycelium does not form clamp connections. §Fungi used in the grow assay experiment.

### *In vitro* fungal growth assays

Fungal growth assays were performed as described by Mu et al. (1999). Briefly, five out of 14 morphotypes (marked § in Table 1), which did not sporulate profusely in culture, were selected for the growth assays. The well-known pathogenic Basidiomycete fungus, *Heterobasidion annosum* TC32-1 (Olson et al., 2012), was included in the growth assays. Agar plugs (5-mm diameter) from each fungus were equidistantly placed in 150φ x 20 mm Petri plates (Sarstedt) containing solid MMN or PDA, amended with increasing concentrations (i.e., 0, 23, 47, 70, and 94 μM) of paclitaxel (Cayman Chemical Company, Michigan-USA) dissolved in dimethyl sulfoxide (DMSO). The assays were done in triplicates for each concentration, and the Petri plates were scanned daily. The images were analyzed at the end of the experiment.

### Area and mycelial growth rate determination

Fungal growth rate was determined as described in Montini et al. (2006). Briefly, using the ImageJ software (Schneider et al., 2012), the growth area for each fungus was calculated by outlining the edge of the colony and converting the pixel area to mm^2^ after proper calibration. The mycelial growth area was plotted as a function of time (mm^2^/day) and used in the statistical analysis. The radial growth rate (K_r_) was calculated as defined by Trinci (1971), where growth rate = (radius_1_-radius_0_) / (time_1_-time_0_) (Trinci, 1971). Only the days corresponding to the exponential growth phase were included in the calculations (Meletiadis et al., 2001; Montini et al., 2006).

### Statistical analysis

A one-way analysis of variance (ANOVA) test and Duncan *post-hoc* analysis (*p* < 0.01) were used to determine the effects of the paclitaxel treatment on the mycelia growth rate.

### RNA and gDNA extraction, CDNA synthesis, PCR of the β-tubulin genes, and ITS sequence

Agar plugs from fungi (Table 1), grown in solid MMN, were inoculated into 250-ml Erlenmeyer flasks containing 50 ml of MMN liquid medium and grown at 25°C without shaking for 6 days. Mycelia were harvested by centrifugation, lyophilized, and then grounded to a powder using a mortar and pestle cooled with liquid nitrogen. RNA extraction was done using the RNeasy Plant Mini Kit (Qiagen) according to the manufacturer's protocol. One μg of total RNA was reverse-transcribed with the iScript cDNA Synthesis Kit (Bio-Rad) according to the manufacturer's instructions. Primers F-βtub1 (5'-CAR RCY GGT CAR TGY GGT AAC CA-3') and βtub4r (5'-GCC TCM GTR AAY TCC ATY TCR TCC AT-3') were purchased from Eurofins Genomics Denmark A/S (Denmark) and used to amplify the β-tubulin genes from cDNA (Einax and Voigt, 2003).

gDNA was extracted using the NucleoSpin Plant II kit (Macherey-Nagel GmbH & Co. KG, Düren, Germany) by following their protocol. The internal transcribed spacer (ITS) using primers ITS1F (5'-CTT GGT CAT TTA GAG GAA GTA A-3') and ITS4 (5'-TCC TCC GCT TAT TGA TAT GC-3') was amplified and sequenced to identify the endophytic fungi at the molecular level (White et al., 1990; Gardes and Bruns, 1993).

Polymerase chain reactions were done using Phusion Hot Start II DNA Polymerase (Thermo Scientific™) following the manufacturer's protocol, but annealing temperatures were carried at 59°C and 58°C for the beta-tubulin gene and ITS sequence, respectively. PCR products were gel-purified using the GeneJET Gel Extraction Kit (Thermo Scientific™), cloned into pJet1.2 (Thermo Scientific™), and transformed into One Shot^®^ TOP10 Chemically Competent *E. coli* (Invitrogen). Plasmids were isolated using the GeneJET Plasmid Miniprep Kit (Thermo Scientific™) and sent to Macrogen Europe (The Netherlands) for sequencing using the universal primers provided by the facility.

### Phylogenetic analysis

The β-tubulin genes sequences were translated, and the Molecular Evolutionary Genetics Analysis (MEGA) software was used to align and analyze the protein sequences. The analysis only included the 428 amino acids that have been implicated in the paclitaxel-β-tubulin interaction. In addition, GenBank protein sequences from fungal endophytes isolated from *Taxus* spp. or from fungi deemed able to synthesize paclitaxel were included in the analysis (Supplementary Table S2). For comparison, the human and *Taxus* β-tubulins protein sequences were also included. The online application MEME (Mixed Effects Model of Evolution: www.datamonkey.org) was used to test for episodic positive/diversifying selection, but the analysis was limited to those organisms isolated as endophytes from *Taxus* spp. or able to produce paclitaxel.

## Results

### *In vitro* fungal growth assay

**Figure 2:**
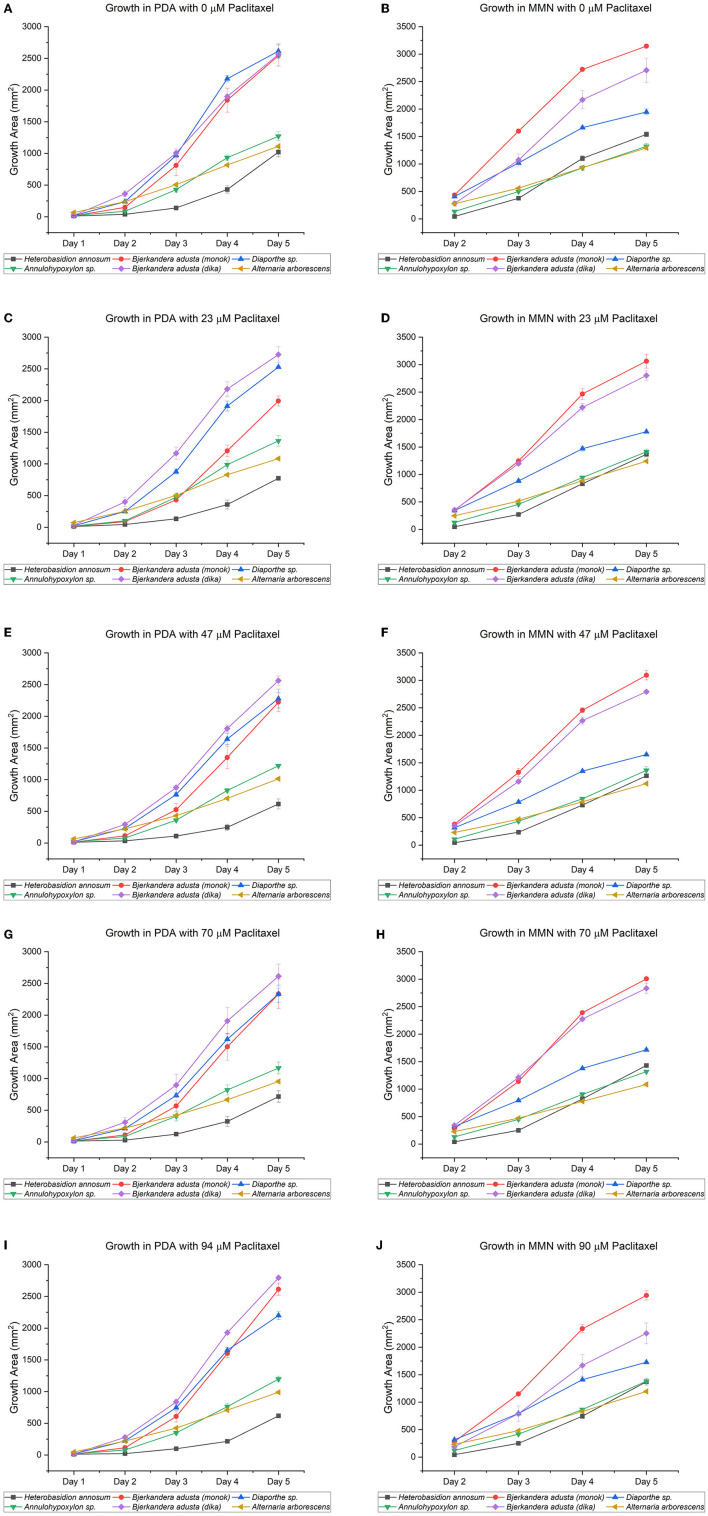
Growth of endophytic fungi isolated from *Taxus wallichiana* Zucc., compared to *Heterobasidion annosum* TC32-1, a well-known conifer pathogen. The five fungi, which did not sporulate profusely in culture, were chosen from 14 morphotypes. Both media, potato dextrose agar [PDA; graphs **(A,C,E,G,I)**] and Minimal Melin Norkrans [MMN; graphs **(B,D,F,H,J)**], contained increasing concentrations of paclitaxel (i.e., 0, 23, 47, 70, and 94 μM). DMSO was added to the 0 μM paclitaxel plates. The MMN values for “Day 1” are missing due to COVID-19 restrictions when the experiments were performed. All the endophytic fungi, as well as *H. annosum*, grew in both media containing different concentrations of paclitaxel, though nutritional differences may influence the capability of fungi to tolerate, degrade, or detoxify paclitaxel. Only the days corresponding to the exponential growth phase (Days 2–5) were included in the calculations (Meletiadis et al., 2001; Montini et al., 2006). The bars indicate the standard error.

**Figure 3:**
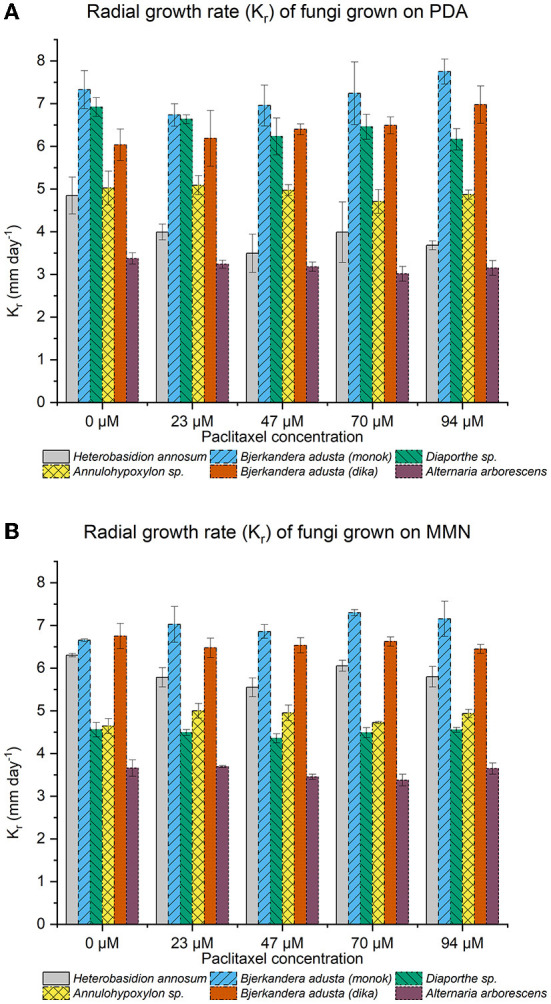
**(A,B)** Radial growth rate (K_r_) for fungi grown at increasing concentrations of paclitaxel in potato dextrose agar (PDA) or Minimal Melin Norkrans (MMN) media. The radial growth rate [K_r_ = (radius_1_-radius_0_)/(time_1_-time_0_)] was calculated as a linear regression (Trinci, 1971). The fungi displayed different radial growth rates, which remained fairly constant when grown in either medium, regardless of the concentration of paclitaxel used. However, the radial growth rate for *H. annosum* was lower when grown in PDA, while *Diaporthe* sp. strain PAR5 showed a decrease when grown in MMN. Only the days corresponding to the exponential growth phase (Days 2–5) were included in the calculations. The bars indicate the standard deviation.

All the endophytic fungi, as well as *H. annosum*, grew in both media containing different concentrations of paclitaxel (i.e., 0, 23, 47, and 94 μM, respectively) (Tables 2, 3; Figure 2). When grown in MMN, the growth area (GA) for *H. annosum* was higher compared to when it was grown in PDA (*P* < 0.001) (Tables 2, 3; Figure 2). Though *Diaporthe* sp. strain PAR5 showed a higher GA when grown in MMN, compared to when it was grown in PDA, it was not statistically significant (Tables 2, 3; Figure 2). The Basidiomycetes, *B. adusta* strain PAR4 (monokaryon) and *B. adusta* strain PAR7 (dikaryon), showed a significantly higher GA than *H. annosum*, regardless of the media used (*P* < 0.001) (Tables 2, 3; Figure 2). *Alternaria arborescens* strain PAR15 had a significantly smaller GA than the other fungi irrespective of the medium (*P* < 0.001) (Tables 2, 3; Figure 2). Though *A. arborescens* strain PAR15 had similar GA as *H. annosum* on MMN, it had significantly higher GA than *H. annosum* on PDA (*P* < 0.001). The *Annulohypoxylon* sp. strain MUS1, known to produce paclitaxel, showed an identical GA as *A. arborescens* strain PAR15 irrespective of the medium, and it showed a slight but significant (*P* < 0.05) increase in the GA when it was grown in MMN (Tables 2, 3; Figure 2). The fungi displayed different radial growth rates (K_r_) and when grown in either medium, the K_r_ remained relatively constant regardless of the concentration of paclitaxel used (Figure 3; Supplementary Table S1). However, the K_r_ of *H. annosum* was significantly lower when grown in PDA (*P* < 0.001), while *Diaporthe* sp. strain PAR5 showed a decrease in the K_r_ when grown in MMN. Though not significant, *B. adusta* strain PAR4 (monokaryon) and *B. adusta* strain PAR7 (dikaryon) showed a slight increase in the K_r_ when grown in MMN. Similarly, *Annulohypoxylon* sp. strain MUS1 showed a slight increase in the K_r_ when grown in MMN. All the endophytic yeasts isolated (i.e., *Kurtzmaniella quercitrusa* strain PAR14, *Hyphopichia* sp. strain PAR10a and *Meyerozyma guilliermondii* strain MUS2) were able to grow at all the different concentrations of paclitaxel tested (data not shown).

**Table 2:** Comparison of growth area for the fungi grown in PDA media at increasing concentrations of paclitaxel.

	***H. annosum*** **TC32-1**	***B. adusta*** **strain PAR4**	***Diaporthe*** **sp.** **strain PAR5**	***Annulohypoxylon*** **sp.** **strain MUS1**	***B. adusta*** **strain PAR7**	***A. arborescens*** **strain PAR15**
0 μM paclitaxel						
Day 1	12.8 ± 0.8	15.2 ± 0.9	18.4 ± 1.3	20.4 ± 1.4	21.2 ± 3.6	67.8 ± 2.6
Day 2	38.8 ± 2.7	147.9 ± 37.4	237.9 ± 12.4	86.4 ± 10.7	361.6 ± 46.3	238.9 ± 6.8
Day 3	139.1 ± 4.2	810.4 ± 160.0	968.5 ± 35.6	428.8 ± 41.7	1,006.1 ± 61.5	507.5 ± 8.7
Day 4	430.6 ± 58.1	1,840.1 ± 189.4	2,178.3 ± 52.2	931.9 ± 40.5	1,897.7 ± 33.4	815.6 ± 3.0
Day 5	1,019.7 ± 74.2	2,544.9 ± 165.6	2,614.1 ± 113.1	1,272.3 ± 71.3	2,563.4 ± 30.5	1,112.5 ± 16.8
23 μM paclitaxel						
Day 1	13.5 ± 0.2	14.0 ± 0.2	23.3 ± 1.4	19.7 ± 1.7	30.9 ± 5.5	72.4 ± 0.9
Day 2	43.9 ± 1.1	90.1 ± 7.6	250.7 ± 9.5	102.9 ± 14.9	400.4 ± 72.9	253.2 ± 1.9
Day 3	133.2 ± 7.9	431.4 ± 1.4	877.4 ± 36.4	474.3 ± 66.3	1,167.4 ± 95.9	502.9 ± 2.5
Day 4	359.2 ± 70.0	1,205.5 ± 93.8	1,912.6 ± 78.8	988.3 ± 68.3	2,181.3 ± 112.6	828.4 ± 2.3
Day 5	772.4 ± 24.4	1,992.8 ± 79.7	2,530.5 ± 11.8	1,364.3 ± 82.9	2,725.2 ± 121.8	1,083.8 ± 17.6
47 μM paclitaxel						
Day 1	12.9 ± 0.2	14.2 ± 0.4	22.2 ± 0.6	19.0 ± 1.0	19.4 ± 2.4	65.4 ± 1.7
Day 2	34.1 ± 6.0	113.9 ± 14.8	230.6 ± 12.2	78.4 ± 7.7	294.4 ± 21.2	224.6 ± 2.2
Day 3	109.7 ± 9.4	526.5 ± 94.6	764.2 ± 32.6	360.2 ± 24.2	875.3 ± 29.3	430.4 ± 0.9
Day 4	247.8 ± 47.5	1,349.7 ± 178.9	1,641.8 ± 89.3	829.7 ± 28.9	1,807.0 ± 54.3	704.6 ± 12.8
Day 5	615.4 ± 79.4	2,223.1 ± 149.2	2,279.5 ± 149.2	1,220.0 ± 8.0	2,563.3 ± 72.9	1,013.3 ± 19.3
70 μM paclitaxel						
Day 1	14.3 ± 0.3	15.6 ± 0.4	20.8 ± 0.5	18.8 ± 1.9	22.9 ± 4.4	63.6 ± 0.7
Day 2	28.3 ± 2.9	109.6 ± 21.0	211.3 ± 17.4	85.4 ± 18.3	310.4 ± 71.1	220.1 ± 0.1
Day 3	123.7 ± 3.2	566.3 ± 83.4	733.4 ± 37.4	406.8 ± 76.6	897.0 ± 171.8	419.8 ± 6.4
Day 4	324.2 ± 77.2	1,501.2 ± 212.8	1,621.9 ± 87.5	819.4 ± 80.4	1,907.8 ± 212.0	665.7 ± 20.7
Day 5	715.6 ± 91.4	2,333.0 ± 231.1	2,334.2 ± 137.8	1,165.6 ± 94.3	2,613.9 ± 189.3	953.7 ± 29.0
94 μM paclitaxel						
Day 1	11.9 ± 0.9	13.6 ± 0.3	18.6 ± 0.8	17.8 ± 0.3	17.5 ± 0.4	47.4 ± 0.9
Day 2	22.3 ± 4.4	114.2 ± 33.9	223.0 ± 7.2	78.1 ± 5.7	279.8 ± 34.9	220.4 ± 12.3
Day 3	99.4 ± 15.1	607.0 ± 89.0	745.6 ± 22.0	349.2 ± 29.3	837.2 ± 40.9	425.0 ± 15.9
Day 4	215.6 ± 18.7	1,601.1 ± 66.1	1,652.3 ± 41.3	763.8 ± 50.5	1,929.1 ± 5.3	708.0 ± 20.6
Day 5	618.1 ± 33.4	2,612.8 ± 97.9	2,199.6 ± 61.9	1,197.8 ± 44.0	2,795.2 ± 29.2	988.7 ± 24.2

Values are means (mm^2^) ± standard error. Day 0 was considered the day the fungal plug (5-mm diameter) was placed mycelium-faced to the culture media. DMSO was added to the 0 μM paclitaxel plates. Days 2–5 were considered the exponential growth phase and used in the analysis (Meletiadis et al., 2001; Montini et al., 2006).

**Table 3:** Comparison of growth area for the fungi grown in MMN media at increasing concentrations of paclitaxel.

	***H. annosum*** **TC32-1**	***B. adusta*** **strain PAR4**	***Diaporthe*** **sp.** **strain PAR5**	***Annulohypoxylon*** **sp.** **strain MUS1**	***B. adusta*** **strain PAR7**	***A. arborescens*** **strain PAR15**
0 μM paclitaxel						
Day 2	43.9 ± 6.2	434.8 ± 2.2	406.8 ± 6.9	134.6 ± 7.5	280.3 ± 37.8	276.1 ± 4.4
Day 3	375.5 ± 21.2	1,598.2 ± 10.8	1,017.9 ± 16.9	497.8 ± 10.8	1,072.7 ± 110.1	559.7 ± 7.1
Day 4	1,101.3 ± 46.7	2,722.5 ± 33.4	1,662.8 ± 25.4	928.8 ± 41.5	2,169.6 ± 166.2	931.9 ± 22.6
Day 5	1,539.8 ± 45.1	3,145.4 ± 3.3	1,948.0 ± 54.8	1,319.7 ± 55.8	2,706.7 ± 220.2	1,292.3 ± 36.5
23 μM paclitaxel						
Day 2	47.8 ± 1.2	347.7 ± 27.9	343.7 ± 11.7	124.8 ± 3.1	355.3 ± 16.7	248.8 ± 4.6
Day 3	272.5 ± 5.2	1,242.7 ± 52.0	882.1 ± 33.1	457.2 ± 16.6	1,198.7 ± 1.6	515.5 ± 5.3
Day 4	831.4 ± 15.0	2,466.2 ± 104.7	1,470.4 ± 43.9	943.2 ± 25.0	2,221.6 ± 68.7	886.2 ± 5.3
Day 5	1,366.9 ± 48.4	3,062.5 ± 125.6	1,779.7 ± 6.8	1,414.1 ± 47.1	2,801.7 ± 84.9	1,240.9 ± 11.4
47 μM paclitaxel						
Day 2	44.5 ± 5.6	381.5 ± 27.7	317.2 ± 3.4	108.3 ± 8.2	353.2 ± 20.6	232.0 ± 4.4
Day 3	234.4 ± 2.2	1,325.2 ± 73.4	784.4 ± 19.8	434.9 ± 28.5	1,159.7 ± 23.2	470.4 ± 1.5
Day 4	729.5 ± 21.4	2,457.0 ± 43.7	1,348.3 ± 15.0	844.7 ± 23.1	2,267.3 ± 65.7	789.9 ± 10.8
Day 5	1,263.0 ± 29.7	3,093.9 ± 86.4	1,650.8 ± 18.0	1,362.8 ± 66.2	2,793.6 ± 26.6	1,121.3 ± 15.7
70 μM paclitaxel						
Day 2	40.2 ± 1.9	281.1 ± 10.7	320.5 ± 4.0	129.3 ± 9.6	339.6 ± 20.2	226.2 ± 5.4
Day 3	249.6 ± 14.5	1,138.8 ± 18.0	793.4 ± 6.4	448.9 ± 15.6	1,211.1 ± 65.1	470.3 ± 12.4
Day 4	821.8 ± 43.5	2,390.5 ± 6.4	1,376.6 ± 21.5	903.9 ± 34.0	2,275.2 ± 39.6	777.4 ± 18.5
Day 5	1,430.2 ± 22.7	3,006.5 ± 34.4	1,716.2 ± 21.5	1,318.6 ± 29.2	2,834.3 ± 90.8	1,084.3 ± 37.4
94 μM paclitaxel						
Day 2	43.4 ± 2.4	281.8 ± 12.7	316.3 ± 2.6	121.3 ± 7.9	189.0 ± 45.1	234.2 ± 3.3
Day 3	251.1 ± 11.1	1148.6 ± 13.1	793.4 ± 16.8	418.5 ± 13.5	788.9 ± 140.3	480.8 ± 5.4
Day 4	744.3 ± 40.9	2,336.3 ± 72.9	1,408.1 ± 17.2	864.8 ± 31.3	1,668.5 ± 198.2	830.3 ± 14.1
Day 5	1,371.6 ± 61.3	2,942.2 ± 82.8	1,727.0 ± 11.0	1,382.3 ± 21.9	2,252.4 ± 187.8	1,194.0 ± 23.6

Values are means (mm^2^) ± standard error. Day 0 was considered the day the fungal plug (5-mm diameter) was placed mycelium-faced to the culture media. Values for Day 1 are missing. DMSO was added to the 0 μM paclitaxel plates. Days 2–5 were considered the exponential growth phase and used in the analysis (Meletiadis et al., 2001; Montini et al., 2006).

### β-tubulin genes and ITS sequences

A partial β-tubulin coding-sequence was successfully amplified for the 14 fungal endophytes chosen. The β-tubulin sequence and the ITS sequence for each fungus were deposited in GenBank. However, a different forward primer (ATG AGA GAA ATT ATT CAC TTG) was used to amplify the β-tubulin for *M. guilliermondii* strain MUS2. The accession numbers are included in Table 1.

### Phylogenetic analysis

**Figure 4:**
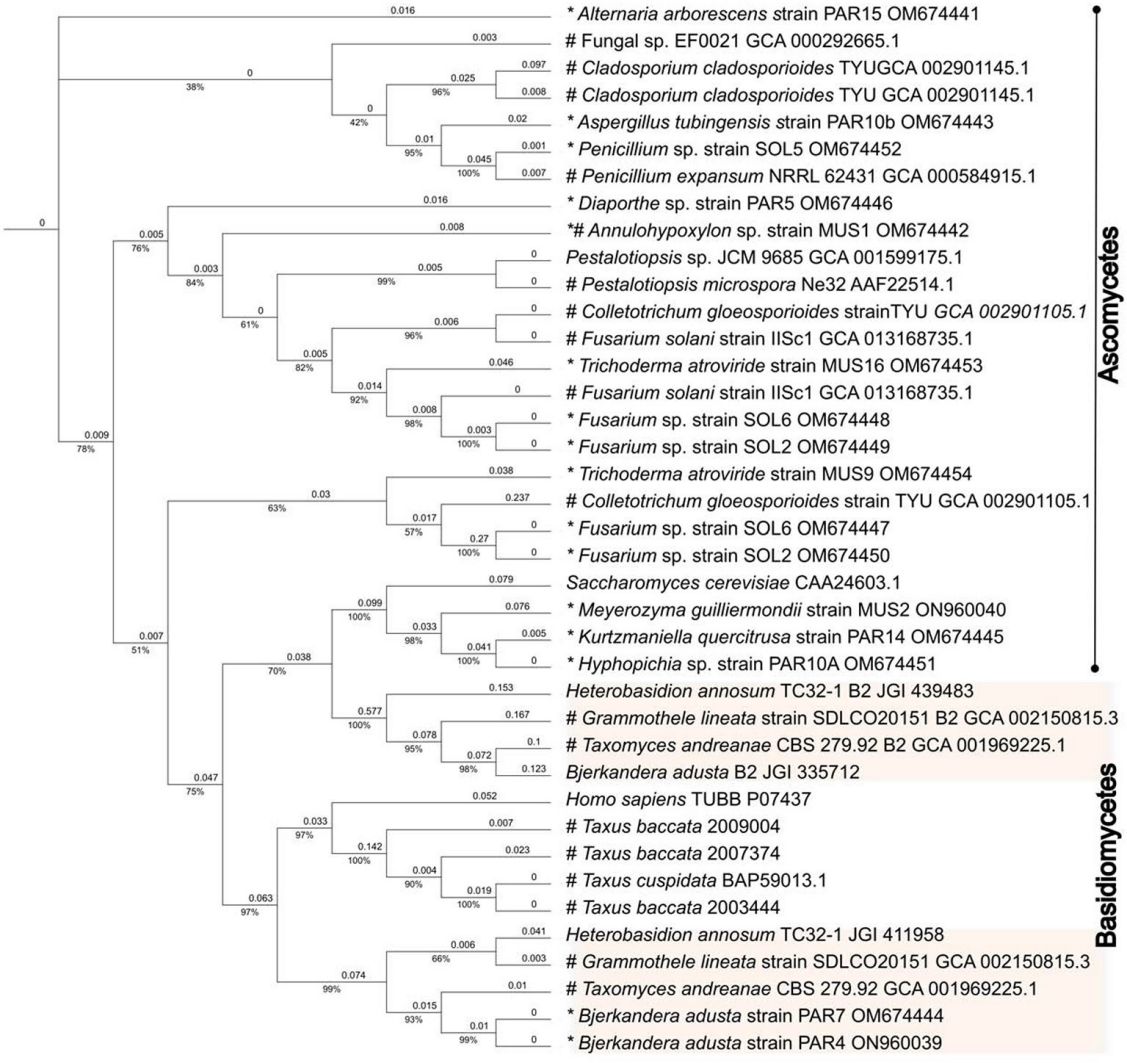
Evolutionary analysis of the β-tubulins proteins. The evolutionary history was inferred by using the IQ-TREE web server (Trifinopoulos et al., 2016) and edited using the Interactive Tree Of Life (iTOL) online tool (Letunic and Bork, 2021). The analysis was done using the default parameters. The analysis only included the 428 amino acids that have been implicated in the paclitaxel-β-tubulin interaction. In addition, GenBank protein sequences from fungal endophytes isolated from *Taxus* spp. or from fungi deemed able to synthesize paclitaxel were included in the analysis. For comparison, the human and *Taxus* β-tubulins protein sequences were also included. The phylogenetic tree generated formed branches that clearly allocated the β-tubulins along the Ascomycetes and Basidiomycetes divisions. The β-tubulins from *Taxus* spp. and the most highly expressed human β-tubulin (P07437) clustered together in a separate branch with the Basidiomycete β-tubulins. The *Taxomyces andreanae* β_1_-tubulin also clustered in this group, and the β_2_ tubulin clustered with the rest of the β_2_ tubulin from Basidiomycete fungi in a separate clade. This would suggest that *T. andreanae* CBS 279.92 belongs in the phylum Basidiomycota, rather than Ascomycota as originally proposed. An asterisk (*) indicates the fungi isolated by Gauchan et al. (2021), which were used in this study, and the pound sign (#) indicates plant/fungus that can synthesize paclitaxel. JGI numbers refer to protein sequence number available from the Joint Genome Institute (https://mycocosm.jgi.doe.gov/mycocosm/home).

**Figure 5:**
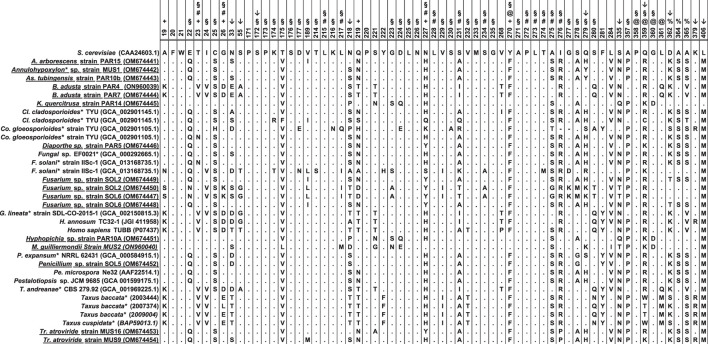
Amino acid sequence comparison of the β-tubulin proteins. The alignment included fungi isolated in this study (shown underlined) and compared to published sequences from organisms that have been shown to produce paclitaxel (indicated with an asterisk), or other fungal endophytes (except for *H. annosum*). For comparison, the β-tubulins from *Taxus* spp. and the most highly expressed human β-tubulin (P07437) were also included. Amino acids are numbered and used as consensus according to the β-tubulin protein of *Saccharomyces cerevisiae* (CAA24603.1). Symbols above the numbering indicate the following: +: the residues A19K, T23V, G26D, N227H, and Y270F which were mutated by Gupta et al. (2003) to create a paclitaxel-sensitive *S. cerevisiae* strain; #: the residues that were shown to interact directly with paclitaxel Löwe et al. (2001); @: the residues that form the binding pocket but have no direct interaction with paclitaxel (Löwe et al., 2001; Tuszynski et al., 2012); §: the residues that bind paclitaxel as defined by Tuszynski et al. (2012). Residue at position 219 was initially suggested by Mu et al. (1999) to be important for the interaction with paclitaxel (T = sensitive; N/Q = resistant). Positions with an arrow (↓), are the amino acids that, according to the MEME analysis, show episodic positive/diversifying selection (default p-value threshold of 0.1). Four of these positions (i.e., 172, 279, 359, and 362) have been implicated in the binding of paclitaxel by β-tubulin or formed part of the binding pocket. The alignment was done with MEGA X using the ClustalW alignment program (Kumar et al., 2018). Sequences used in the analysis are included in the Supplementary File S1.

Only 428 amino acids were used in the phylogenetic analysis, which also included β-tubulin protein sequences from paclitaxel producers as well as non-producers found in GenBank (Supplementary Table S2). When analyzed by the maximum-likelihood approach, the phylogenetic tree generated formed branches that clearly allocated the β-tubulins along the Ascomycetes and Basidiomycetes divisions (Figure 4). The β-tubulins from *Taxus* spp. and the most highly expressed human β-tubulin (P07437) clustered together in a separate branch with the rest of the Basidiomycete β-tubulins. Surprisingly, the *Taxomyces andreanae* β_1_-tubulin also clustered in this group, and the β_2_ tubulin clustered with the rest of the β_2_ tubulin from Basidiomycete fungi in a separate clade. This would suggest that *T. andreanae* CBS 279.92 belongs in the phylum Basidiomycota, rather than Ascomycota as originally proposed. The β-tubulin from the endophytic yeasts (i.e., *K. quercitrusa* strain PAR14, *M. guilliermondii* strain MUS2, and *Hyphopichia* sp. strain PAR10a) clustered together with *S. cerevisiae* and was similar at four out of the five key amino acids (i.e., Ala19, Thr23, Gly26, and Tyr270) initially mutated to create a paclitaxel-sensitive yeast (Figure 5). The remaining clades were composed of Ascomycete fungi, with the β_2_- and β_1_-tubulins distributed among the clades. The β_2_- or β_1_-tubulins from paclitaxel-producing fungi (indicated with an asterisk) did not cluster together (Figure 4). This would suggest that β-tubulins are not a good predictor of fungi capable of synthesizing paclitaxel.

To test the idea that there is a correlation between amino acid changes and the capacity to grow in the presence of paclitaxel, the online application MEME (Mixed Effects Model of Evolution) was used to analyze sequences from fungal endophytes isolated from *Taxus* spp. as well as organisms shown to synthesize paclitaxel. MEME uses a mixed-effects maximum likelihood approach looking for individual sites under positive selection and found evidence of episodic positive/diversifying selection at 10 sites (default p-value threshold of 0.1) in the β-tubulin genes, corresponding to codon positions 33, 55, 172, 218, 279, 335, 359, 362, 379, and 406. Five of these positions (i.e., 172, 218, 279, 359, and 362) have been implicated in the binding of paclitaxel by β-tubulin (Figure 5).

## Discussion

Endophytic fungi that reside in paclitaxel-producing plants are exposed to various concentrations of this metabolite depending on whether they reside in the leaves or in the bark, the age of the plant, and the environmental conditions (Kelsey and Vance, 1992; Wheeler et al., 1992; Mu et al., 1999). It has been known that the β-tubulin protein is the intended target of paclitaxel, and early studies with the paclitaxel-producing fungus, *P. microspora* Ne32, identified two regions in the β-tubulin protein involved in the binding of paclitaxel, with amino acid residue 219 being a Thr for susceptible fungi or Asn or Gln for resistant fungi (Mu et al., 1999) (Figure 5). Further studies were done to refine the structure of the β-tubulin protein using X-ray crystallography, and five key amino acid changes (i.e., Ala19Lys, Thr23Val, Gly26Asp, Asn227His, and Tyr270Phe) were introduced in *S. cerevisiae* to create a paclitaxel-sensitive yeast (Löwe et al., 2001; Gupta et al., 2003). The analysis of the interactions between β-tubulin and paclitaxel showed that the amino acid residues that were in direct contact with the paclitaxel molecule were Val23, Asp26, Leu215, Leu217, His227, Leu228, Ala231, Ser234, Pro272, Leu273, Thr274, Ser275, and Arg276 (Löwe et al., 2001), while amino acids Pro358, Arg359, Gly360, and Leu361 formed the binding pocket in which Phe270 was a key residue (Löwe et al., 2001; Tuszynski et al., 2012). Tuszynski et al. (2012) defined the binding sites of paclitaxel to be residues 22–26, 172–177, 214–217, 223–235, 270–280, and 357–360 and compared the different β-tubulin isotypes of humans and yew trees (*Taxus* spp.). Based on their calculations, mutations at positions 24, 25, 26, 229, 280, and 359 would likely affect the binding energy of paclitaxel to β-tubulin, and later, this was validated in part by Kudo et al. (2014). A similar concept proposed in the 1990's suggested that the differences in paclitaxel sensitivity exhibited by different fungi were mainly due to the modifications in the β-tubulin protein (Elmer et al., 1994; Mu et al., 1999). Consequently, endophytic fungi isolated from *Taxus* plants would have evolved to encode a β-tubulin protein that is insensitive to paclitaxel. We explored this idea using endophytic fungi isolated from the leaves and bark of the Himalayan Yew [*Taxus wallichiana* Zucc.; (Gauchan et al., 2021)], focusing only on the 428 amino acids of the β-tubulin that have been shown to be interacting with paclitaxel.

The β-tubulin gene sequences have been used in phylogenetic analysis to study the evolutionary relationships among fungi, and therefore, it has been known that most Ascomycete fungi encode one β-tubulin gene, while Basidiomycetes have two, with different evolutionary mechanisms driving their diversification (Zhao et al., 2015). Nevertheless, some Ascomycete fungi encode two β-tubulin genes, for example, certain species of *Aspergillus, Trichoderma, Colletotrichum, Cladosporium*, and *Fusarium*. Many fungal endophytes isolated from paclitaxel-producing plants belong to these genera and in fact, the paclitaxel producers, *F. solani* Strain IISc-1, *Co. gloeosporioides* Strain TYU, and *Cl. cladosporioides* TYU, encode two β-tubulin genes (Chakravarthi et al., 2008; Miao et al., 2018). As expected, the *Trichoderma* and *Fusarium* spp., isolated in our study, also encoded two β-tubulin genes (Table 1 and Figure 4).

Not surprisingly, the alignment of the β-tubulin proteins from the endophytes in our study and from known paclitaxel producers (as well as fungi deemed to be sensitive to paclitaxel) formed distinct clades along the Ascomycete and Basidiomycete divisions (Figure 4). Interestingly, the β-tubulins from *Taxus* spp. and the most highly expressed human β-tubulin (Tuszynski et al., 2012) clustered together in a separate branch with the rest of the Basidiomycete β-tubulins (Figure 4). We were surprised to see *T. andreanae* CBS 279.92, which was the first fungus purported to synthesize paclitaxel (Stierle et al., 1993), clustering together with other Basidiomycetes rather than Ascomycetes (Figure 4). Based on phenotypic traits, the original patent filed in 1994 indicated that *T. andreanae* could be related to *Oidium, Rhinotrichum*, or *Monilia* (Strobel et al., 1994). Further mining of the *T. andreanae* genome (GenBank assembly accession GCA_001969225.1) yielded the second β-tubulin gene, a partial 5.S RNA sequence, and both the small (SSU) and large subunit (LSU) ribosomal RNA genes. Based on comparisons of the SSU and LSU sequences, *T. andreanae* CBS 279.92 should be placed under the Phanerochaetaceae family and most likely belongs in the genus *Ceriporiopsis*, with *C. gilvescens* being the closest relative (data not shown). We were unable to obtain *T. andreanae* CBS 279.92 from the culture collection (Westerdijk Institute) to further validate our findings by ITS sequencing since the fungus is not available from their public collection (personal email communication). In addition, we were surprised to see that isolate PAR4, chosen before the ITS and β-tubulin genes were amplified and sequenced, was another isolate of *Bjerkandera adusta*. Unlike *B. adusta* strain PAR7, strain PAR4 did not form clamp connections in culture. When looking at the sites 24, 25, 26, 229, 280, and 359, which were mentioned by Tuszynski et al. (2012), no clear pattern emerged. However, amino acids at positions 24, 25, 26, and 280 showed more variation than for positions 229 and 359, and none of the fungi showed the same sequence as the most highly expressed β-tubulin protein from humans.

To test the idea that there is a correlation between amino acid changes and the ability to grow in the presence of paclitaxel, the Mixed Effects Model of Evolution (MEME; www.datamonkey.org) was used to analyze sequences from fungal endophytes isolated from *Taxus* spp., as well as organisms shown to synthesize paclitaxel. MEME, which uses a mixed-effects maximum likelihood approach looking for individual sites under positive selection (Murrell et al., 2012), found evidence of episodic positive/diversifying selection at 10 sites (default *p*-value threshold of 0.1) in the β-tubulin sequences, corresponding to codon positions 33, 55, 172, 218, 279, 335, 359, 362, 379, and 406. Four of these positions (i.e., 172, 279, 359, and 362) have been implicated in the binding of paclitaxel by β-tubulin or formed part of the binding pocket [(Tuszynski et al., 2012); Figure 5]. The apparent significance for codon at positions 33, 55, 218, 335, 379, and 406 is not very clear to us. However, these may be sites for post-transcriptional modifications to the β-tubulin protein, which to our knowledge have not been studied in fungi in the context of paclitaxel resistance. For example, post-transcriptional modification (i.e., phosphorylation) on Ser172 by CDK1 inhibits tubulin assembly into microtubules during parts of the cell cycle; thus, it is not surprising that serine is conserved at this position (Fourest-Lieuvin et al., 2006). Though the amino acid at position 55 is not conserved, for humans there are data suggesting that phosphorylation at Thr55 is also possible (Zhou et al., 2013). Most of the fungi showed a serine, glycine, or alanine, except for *F. solani* Strain IISc-1, which also showed a threonine for its second β-tubulin at this position (Figure 5). Moreover, resistance of cancer cells to paclitaxel has implicated position 218, which in humans is Ala in the βIII-tubulin isotype (Yang et al., 2016). In most of the fungi, serine occupied this position, followed by threonine, proline, asparagine, and alanine (Figure 5). Nevertheless, our data only focused on 428 amino acids, and thus, we cannot exclude that post-translational modifications could be taking place on the variable carboxyl terminus of the β-tubulin proteins that could also be part of the interaction with paclitaxel.

As shown by the growth assays, the endophytes tested (i.e., *Bjerkandera adusta* strain PAR4; *Diaporthe* sp. strain PAR5; *B. adusta* strain PAR7; *Alternaria arborescens* strain PAR15; and *Annulohypoxylon* sp. strain MUS1) were able to grow at all the paclitaxel concentrations (Figure 2). The assays also included the well-known pathogenic Basidiomycete fungus, *H. annosum*, which had been shown to be sensitive to paclitaxel by Soliman et al. (2013). They suggested that the endophyte, *Paraconiothyrium* SSM001, produces paclitaxel *in planta* to ward off wood-decaying fungi that are known to infect conifers (Soliman et al., 2015). However, in our assays, though the initial growth of *H. annosum* was slower in PDA than in MMN, the fungus was able to grow even at the highest concentration of paclitaxel tested (80 μM). Though we do not dispute the findings of Soliman et al., we contend that the wood-decaying fungi used in their study (*i.e., H. annosum, P. schweinitzii*, and *Pe. subacida*) are mainly root and butt rot-causing pathogens. In fact, our department has worked with *Heterobasidion* species for more than 20 years, and PDA is never used for culturing this fungus. Though paclitaxel growth assays conducted since the 1990s have only used PDA media (Young et al., 1992; Elmer et al., 1994; Mu et al., 1999), our results would underscore the need to conduct growth assays in different media before deciding whether a given fungus is inhibited by paclitaxel. In addition, our results would suggest that nutrition, which has been known to influence antifungal susceptibility assays (Meletiadis et al., 2001), may affect the capability of fungi to tolerate, degrade, or detoxify paclitaxel. Also, variations in *Heterobasidion* strains may account for the apparent discrepancies between our assays and the ones done by Soliman et al. (2013, 2015). Though the idea that paclitaxel is used to award off against wood-degrading fungi has some merit, there is at least one example of a wood-degrading fungus, *Ganoderma carnosum*, that seems to appear exclusively on *Taxus* spp. (Mattock, 2001). In addition, *G. lucidum, G. resinaceum*, and *Laetiporus sulphureus* are known to cause heart rot and decay in *T. baccata*; however, it is thought that they enter through pruning wounds and *via* roots (Phillips and Burdekin, 1982). Nevertheless, new insights seem to indicate that wood-degrading fungi are already present as endophytes or in a latent stage and that their lifestyle changes when the tree dies (Parfitt et al., 2010; Song et al., 2017). The fact that the wood-degrading fungus, *B. adusta* was isolated as an endophyte from *T. wallichiana*, would further support this idea.

Interestingly, some endophytic fungi, isolated from *Taxus* spp. that have been shown to produce paclitaxel, are also pathogens of Yews (e.g., *Alternaria* spp., *Fusarium solani*, and *Cladosporium* spp. *Botrytis* sp.) (Mirski, 2012). Though it is known that certain endophytes can change their lifestyle from mutualistic to a pathogenic state (Khare et al., 2018), the role of paclitaxel in triggering the change to a pathogenic state has not been explored. Furthermore, the ability of some fungi to form protoplast structures inside the plant called mycosomes (Atsatt and Whiteside, 2014) could add another layer of complexity in plant-endophyte-paclitaxel interactions. Nevertheless, experiments have shown that paclitaxel can be used as chemical signal for interaction among endophytic fungi and the plant (Li and Tao, 2009; Soliman and Raizada, 2013). Fungi have the capability to degrade toxic metabolites (Harms et al., 2011), but since the goal has been to increase the production of paclitaxel, no recent studies have been done to elucidate the pathway for paclitaxel degradation. However, early studies suggested that different side chains at the A-ring C-13 (Figure 1) may be important for the increase or decrease in activity of paclitaxel (Young et al., 1992). Later investigations on the biotransformation of paclitaxel analogs showed that some fungal endophytes could modify the functional groups (e.g., oxetane ring, C-10, C-13; Figure 1) and convert the paclitaxel analogs into less active metabolites (Kingston, 1994; Hu et al., 1996; Zhang et al., 1998; Baloglu and Kingston, 1999).

Fungi are known to be biological machines capable of synthesizing complex secondary metabolites. These metabolites can be used as chemical signals for interaction and communication with other microorganisms or as defense mechanisms to inhibit the growth of competitors (Brakhage, 2013). Paclitaxel would also present a challenge to the producing fungus. However, fungi possess different mechanisms to deal with the potential toxic effects of the secondary metabolites they produce. Often, these mechanisms come in the form of efflux transporters that actively pump the toxic metabolite to the outside of the cell or by the employment of two main classes of transporters, the ATP-binding cassette (ABC) and the major facilitator superfamily (MFS) (Martín et al., 2005; Paterson and Lima, 2009). In addition, the fungus could sequester the toxic metabolites, including their precursors, alter their cell wall structure, and even modify the intended target of the metabolite (Martín et al., 2005; Paterson and Lima, 2009).

## Conclusion

We found evidence of episodic positive/diversifying selection at 10 sites in the β-tubulin sequences from fungi isolated from Yews. Four of these positions have been implicated in the binding of paclitaxel by β-tubulin or formed part of the binding pocket. All the fungi tested grew in the presence of paclitaxel. Our results would also underscore the need to conduct growth assays in different media and would suggest that nutrition may influence the capability of fungi to degrade or detoxify paclitaxel. Nevertheless, fungi still possess different mechanisms to deal with the toxic effects of the metabolites they are exposed to or produce. The first fungus shown to produce paclitaxel, *Taxomyces andreanae* CBS 279.92, is a Basidiomycete fungus.

## Data availability statement

The datasets presented in this study can be found in online repositories. The names of the repository/repositories and accession number(s) can be found in the article/Supplementary material.

## Author contributions

HV conceived, designed, performed the experiments, and wrote the manuscript. DG provided the fungal isolates. MG-G contributed to the reagents and materials. HV and MG-G analyzed the data. All authors read, edited, and approved the manuscript.

## Funding

This work was financially supported by a grant from the Swedish Research Council (Ref/348-2012-6138/Agreement/C0613801) to MG-G and DG.

## Conflict of interest

The authors declare that the research was conducted in the absence of any commercial or financial relationships that could be construed as a potential conflict of interest.

## Publisher's note

All claims expressed in this article are solely those of the authors and do not necessarily represent those of their affiliated organizations, or those of the publisher, the editors and the reviewers. Any product that may be evaluated in this article, or claim that may be made by its manufacturer, is not guaranteed or endorsed by the publisher.
