# Supplementary material for: Taxol and β-tubulins from endophytic fungi isolated from the Himalayan Yew, Taxus wallichiana Zucc
Source: Front Microbiol. 2022 Sep 29;13:956855. doi: 10.3389/fmicb.2022.956855 (PMC9557061; doi:10.3389/fmicb.2022.956855)
Supplement: Supplementary file 1 [file Data_Sheet_1.docx]

Supplementary Material

# Supplementary Data

Fasta files:

MEGA alignment: Sequences protein b-tubulins.fasta

MEME: Sequences DNA datamonkey.fasta

MEME analysis result.json (the file can be uploaded and read here: <http://vision.hyphy.org/MEME>)

Taxomyces andreanae LSU ALYI01002927.1.fasta

Taxomyces andreanae 18S RNA ALYI01003129.1.fasta

Taxomyces andreanae 5SRNA partial ALYI01001764.1.fasta

Raw data images:

MMN growth Assays.pdf

PDA growth Assays.pdf

# Supplementary Table S1. Radial growth rate (K_r_) for fungi grown at increasing concentrations of Paclitaxel in two types of media.

|  |  | | | | |
| --- | --- | --- | --- | --- | --- |
|  | Radial Growth Rate in PDA | | | | |
| Fungus |  | | | | |
|  | Paclitaxel Concentration | | | | |
|  |  | | | | |
|  | 0 µM | 23 µM | 47 µM | 70 µM | 94 µM |
|  |  |  |  |  |  |
| *Heterobasidion annosum* | 4.84 (0.43) | 3.99 (0.18) | 3.49 (0.45) | 3.99 (0.71) | 3.68 (0.10) |
| *Bjerkandera adusta* (monok) | 7.33 (0.36) | 6.73 (0.29) | 6.95 (0.39) | 7.24 (0.85) | 7.75 (0.74) |
| *Diaporthe* sp. | 6.92 (0.22) | 6.63 (0.10) | 6.23 (0.43) | 6.46 (0.29) | 6.16 (0.25) |
| *Annulohypoxylon* sp. | 5.02 (0.40) | 5.09 (0.22) | 4.97 (0.13) | 4.70 (0.28) | 4.87 (0.11) |
| *Bjerkandera adusta* (dika) | 6.03 (0.37) | 6.19 (0.65) | 6.40 (0.13) | 6.49 (0.20) | 6.97 (0.44) |
| *Alternaria arborescens* | 3.37 (0.13) | 3.24 (0.09) | 3.18 (0.11) | 3.01 (0.17) | 3.15 (0.17) |
|  |  |  |  |  |  |
|  |  | | | | |
|  | Radial Growth Rate in MMN | | | | |
|  |  |  |  |  |  |
| *Heterobasidion annosum* | 6.30 (0.04) | 5.78 (0.23) | 5.55 (0.22) | 6.05 (0.13) | 5.80 (0.24) |
| *Bjerkandera adusta* (monok) | 6.65 (0.03) | 7.02 (0.42) | 6.86 (0.16) | 7.30 (0.07) | 7.15 (0.41) |
| *Diaporthe* sp. | 4.56 (0.17) | 4.49 (0.07) | 4.35 (0.11) | 4.49 (0.12) | 4.55 (0.06) |
| *Annulohypoxylon* sp. | 4.64 (0.17) | 5.00 (0.17) | 4.95 (0.18) | 4.72 (0.03) | 4.93 (0.10) |
| *Bjerkandera adusta* (dika) | 6.75 (0.30) | 6.47 (0.23) | 6.53 (0.18) | 6.62 (0.11) | 6.45 (0.10) |
| *Alternaria arborescens* | 3.66 (0.19) | 3.69 (0.03) | 3.45 (0.06) | 3.38 (0.14) | 3.65 (0.13) |
|  |  |  |  |  |  |

Standard deviation is shown in parenthesis.

Supplementary Table S2. β-tubulins mined from GeneBank.

|  |  |  |
| --- | --- | --- |
| Organism | GenBank | Reference |
|  |  |  |
|  |  |  |
| *Annulohypoxylon* sp. Strain MUS1 | OM674442 | (Gauchan et al., 2021) |
| *Cladosporium cladosporioides* TYU | GCA 002901145.1 |  |
| *Colletotrichum gloeosporioides* strain TYU | GCA 002901105.1 |  |
| Fungal sp. EF0021 | GCA 000292665.1 | (Heinig et al., 2013) |
| *Fusarium solani* strain IISc1 | GCA 013168735.1 | (Chakravarthi et al., 2008) |
| *Grammothele lineata* strain SDLCO20151 | GCA 002150815.3 | (Das et al., 2017) |
| *Penicillium expansum* NRRL 62431 | GCA 000584915.1 | (Yang et al., 2014, 62431) |
| *Pestalotiopsis microspora* Ne32 | AAF22514.1 | (Mu et al., 1999) |
| *Pestalotiopsis* sp. JCM 9685 | GCA 001599175.1 | (Pulici et al., 1997) |
| *Taxomyces andreanae* CBS 279.92 | GCA 001969225.1 | (Heinig et al., 2013) |
| *Taxus baccata* | 2009004 | (Tuszynski et al., 2012) |
| *Taxus baccata* | 2007374 | (Tuszynski et al., 2012) |
| *Taxus baccata* | 2003444 | (Tuszynski et al., 2012) |
| *Taxus cuspidata* | BAP59013.1 |  |
|  |  |  |

References:

Chakravarthi, B. V. S. K., Das, P., Surendranath, K., Karande, A. A., and Jayabaskaran, C. (2008). Production of paclitaxel by Fusarium solani isolated from Taxus celebica. *Journal of Biosciences* 33, 259–267. doi: 10.1007/s12038-008-0043-6.

Das, A., Ahmed, O., Baten, A. K. M. A., Bushra, S., Islam, M. T., Ferdous, A. S., et al. (2017). Draft Genome Sequence of Grammothele lineata SDL-CO-2015-1, a Jute Endophyte with a Potential for Paclitaxel Biosynthesis. *Genome Announcements* 5, e00556-18. doi: 10.1128/genomeA.00825-17.

Gauchan, D. P., Vélëz, H., Acharya, A., Östman, J. R., Lundén, K., Elfstrand, M., et al. (2021). Annulohypoxylon sp. strain MUS1, an endophytic fungus isolated from Taxus wallichiana Zucc., produces taxol and other bioactive metabolites. *3 Biotech* 11, 152. doi: 10.1007/s13205-021-02693-z.

Heinig, U., Scholz, S., and Jennewein, S. (2013). Getting to the bottom of Taxol biosynthesis by fungi. *Fungal Diversity* 60, 161–170. doi: 10.1007/s13225-013-0228-7.

Mu, J. H., Bollon, A. P., and Sidhu, R. S. (1999). Analysis of β-tubulin cDNAs from taxol-resistant Pestalotiopsis microspora and taxol-sensitive Pythium ultimum and comparison of the taxol-binding properties of their products. *Molecular and General Genetics* 262, 857–868. doi: 10.1007/s004380051151.

Pulici, M., Sugawara, F., Koshino, H., Okada, G., Esumi, Y., Uzawa, J., et al. (1997). Metabolites of Pestalotiopsis spp., endophytic fungi of Taxus brevifolia. *Phytochemistry* 46, 313–319. doi: 10.1016/S0031-9422(97)00285-9.

Tuszynski, J. A., Craddock, T. J. A., Mane, J. Y., Barakat, K., Tseng, C.-Y., Gajewski, M., et al. (2012). Modeling the Yew Tree Tubulin and a Comparison of its Interaction with Paclitaxel to Human Tubulin. *Pharmaceutical Research* 29, 3007–3021. doi: 10.1007/s11095-012-0829-y.

Yang, Y., Zhao, H., Barrero, R. A., Zhang, B., Sun, G., Wilson, I. W., et al. (2014). Genome sequencing and analysis of the paclitaxel-producing endophytic fungus Penicillium aurantiogriseum NRRL 62431. *BMC Genomics* 15, 69. doi: 10.1186/1471-2164-15-69.
