# Supplementary material for: Taxol and β-tubulins from endophytic fungi isolated from the Himalayan Yew, Taxus wallichiana Zucc
Source: Front Microbiol. 2022 Sep 29;13:956855. doi: 10.3389/fmicb.2022.956855 (PMC9557061; doi:10.3389/fmicb.2022.956855)
Supplement: Supplementary file 2 [file Data_Sheet_2.zip › Data Sheet 1.docx]

Supplementary Material

# Supplementary Data

Fasta files:

MEGA alignment: Sequences protein b-tubulins.fasta

MEME: Sequences DNA datamonkey.fasta

MEME analysis result.json.docx (delete the docx to read the file as “json”)

Taxomyces andreanae LSU ALYI01002927.1.fasta

Taxomyces andreanae 18S RNA ALYI01003129.1.fasta

Taxomyces andreanae 5SRNA partial ALYI01001764.1.fasta

Raw data images:

MMN growth Assays.pdf

PDA growth Assays.pdf

# Supplementary Table S1. Radial growth rate (K_r_) for fungi grown at increasing concentrations of Paclitaxel in two types of media.

|  |  | | | | |
| --- | --- | --- | --- | --- | --- |
|  | Radial Growth Rate in PDA | | | | |
| Fungus |  | | | | |
|  | Paclitaxel Concentration | | | | |
|  |  | | | | |
|  | 0 µM | 23 µM | 47 µM | 70 µM | 94 µM |
|  |  |  |  |  |  |
| *Heterobasidion annosum* | 4.84 (0.43) | 3.99 (0.18) | 3.49 (0.45) | 3.99 (0.71) | 3.68 (0.10) |
| *Bjerkandera adusta* (monok) | 7.33 (0.36) | 6.73 (0.29) | 6.95 (0.39) | 7.24 (0.85) | 7.75 (0.74) |
| *Diaporthe* sp. | 6.92 (0.22) | 6.63 (0.10) | 6.23 (0.43) | 6.46 (0.29) | 6.16 (0.25) |
| *Annulohypoxylon* sp. | 5.02 (0.40) | 5.09 (0.22) | 4.97 (0.13) | 4.70 (0.28) | 4.87 (0.11) |
| *Bjerkandera adusta* (dika) | 6.03 (0.37) | 6.19 (0.65) | 6.40 (0.13) | 6.49 (0.20) | 6.97 (0.44) |
| *Alternaria arborescens* | 3.37 (0.13) | 3.24 (0.09) | 3.18 (0.11) | 3.01 (0.17) | 3.15 (0.17) |
|  |  |  |  |  |  |
|  |  | | | | |
|  | Radial Growth Rate in MMN | | | | |
|  |  |  |  |  |  |
| *Heterobasidion annosum* | 6.30 (0.04) | 5.78 (0.23) | 5.55 (0.22) | 6.05 (0.13) | 5.80 (0.24) |
| *Bjerkandera adusta* (monok) | 6.65 (0.03) | 7.02 (0.42) | 6.86 (0.16) | 7.30 (0.07) | 7.15 (0.41) |
| *Diaporthe* sp. | 4.56 (0.17) | 4.49 (0.07) | 4.35 (0.11) | 4.49 (0.12) | 4.55 (0.06) |
| *Annulohypoxylon* sp. | 4.64 (0.17) | 5.00 (0.17) | 4.95 (0.18) | 4.72 (0.03) | 4.93 (0.10) |
| *Bjerkandera adusta* (dika) | 6.75 (0.30) | 6.47 (0.23) | 6.53 (0.18) | 6.62 (0.11) | 6.45 (0.10) |
| *Alternaria arborescens* | 3.66 (0.19) | 3.69 (0.03) | 3.45 (0.06) | 3.38 (0.14) | 3.65 (0.13) |
|  |  |  |  |  |  |

Standard deviation is shown in parenthesis.

Supplementary Table S2. β-tubulins mined from GeneBank.

|  |  |  |
| --- | --- | --- |
| Organism | GenBank | Reference |
|  |  |  |
|  |  |  |
| *Annulohypoxylon* sp. Strain MUS1 | OM674442 | (Gauchan et al., 2021) |
| *Cladosporium cladosporioides* TYU | GCA 002901145.1 |  |
| *Colletotrichum gloeosporioides* strain TYU | GCA 002901105.1 |  |
| Fungal sp. EF0021 | GCA 000292665.1 | (Heinig et al., 2013) |
| *Fusarium solani* strain IISc1 | GCA 013168735.1 | (Chakravarthi et al., 2008) |
| *Grammothele lineata* strain SDLCO20151 | GCA 002150815.3 | (Das et al., 2017) |
| *Penicillium expansum* NRRL 62431 | GCA 000584915.1 | (Yang et al., 2014, 62431) |
| *Pestalotiopsis microspora* Ne32 | AAF22514.1 | (Mu et al., 1999) |
| *Pestalotiopsis* sp. JCM 9685 | GCA 001599175.1 | (Pulici et al., 1997) |
| *Taxomyces andreanae* CBS 279.92 | GCA 001969225.1 | (Heinig et al., 2013) |
| *Taxus baccata* | 2009004 | (Tuszynski et al., 2012) |
| *Taxus baccata* | 2007374 | (Tuszynski et al., 2012) |
| *Taxus baccata* | 2003444 | (Tuszynski et al., 2012) |
| *Taxus cuspidata* | BAP59013.1 |  |
|  |  |  |

References:

Chakravarthi, B. V. S. K., Das, P., Surendranath, K., Karande, A. A., and Jayabaskaran, C. (2008). Production of paclitaxel by Fusarium solani isolated from Taxus celebica. *Journal of Biosciences* 33, 259–267. doi: 10.1007/s12038-008-0043-6.

Das, A., Ahmed, O., Baten, A. K. M. A., Bushra, S., Islam, M. T., Ferdous, A. S., et al. (2017). Draft Genome Sequence of Grammothele lineata SDL-CO-2015-1, a Jute Endophyte with a Potential for Paclitaxel Biosynthesis. *Genome Announcements* 5, e00556-18. doi: 10.1128/genomeA.00825-17.

Gauchan, D. P., Vélëz, H., Acharya, A., Östman, J. R., Lundén, K., Elfstrand, M., et al. (2021). Annulohypoxylon sp. strain MUS1, an endophytic fungus isolated from Taxus wallichiana Zucc., produces taxol and other bioactive metabolites. *3 Biotech* 11, 152. doi: 10.1007/s13205-021-02693-z.

Heinig, U., Scholz, S., and Jennewein, S. (2013). Getting to the bottom of Taxol biosynthesis by fungi. *Fungal Diversity* 60, 161–170. doi: 10.1007/s13225-013-0228-7.

Mu, J. H., Bollon, A. P., and Sidhu, R. S. (1999). Analysis of β-tubulin cDNAs from taxol-resistant Pestalotiopsis microspora and taxol-sensitive Pythium ultimum and comparison of the taxol-binding properties of their products. *Molecular and General Genetics* 262, 857–868. doi: 10.1007/s004380051151.

Pulici, M., Sugawara, F., Koshino, H., Okada, G., Esumi, Y., Uzawa, J., et al. (1997). Metabolites of Pestalotiopsis spp., endophytic fungi of Taxus brevifolia. *Phytochemistry* 46, 313–319. doi: 10.1016/S0031-9422(97)00285-9.

Tuszynski, J. A., Craddock, T. J. A., Mane, J. Y., Barakat, K., Tseng, C.-Y., Gajewski, M., et al. (2012). Modeling the Yew Tree Tubulin and a Comparison of its Interaction with Paclitaxel to Human Tubulin. *Pharmaceutical Research* 29, 3007–3021. doi: 10.1007/s11095-012-0829-y.

Yang, Y., Zhao, H., Barrero, R. A., Zhang, B., Sun, G., Wilson, I. W., et al. (2014). Genome sequencing and analysis of the paclitaxel-producing endophytic fungus Penicillium aurantiogriseum NRRL 62431. *BMC Genomics* 15, 69. doi: 10.1186/1471-2164-15-69.
