# Supplementary figures and images for: Taxol and β-tubulins from endophytic fungi isolated from the Himalayan Yew, Taxus wallichiana Zucc
Source: Front Microbiol. 2022 Sep 29;13:956855. doi: 10.3389/fmicb.2022.956855 (PMC9557061; doi:10.3389/fmicb.2022.956855)

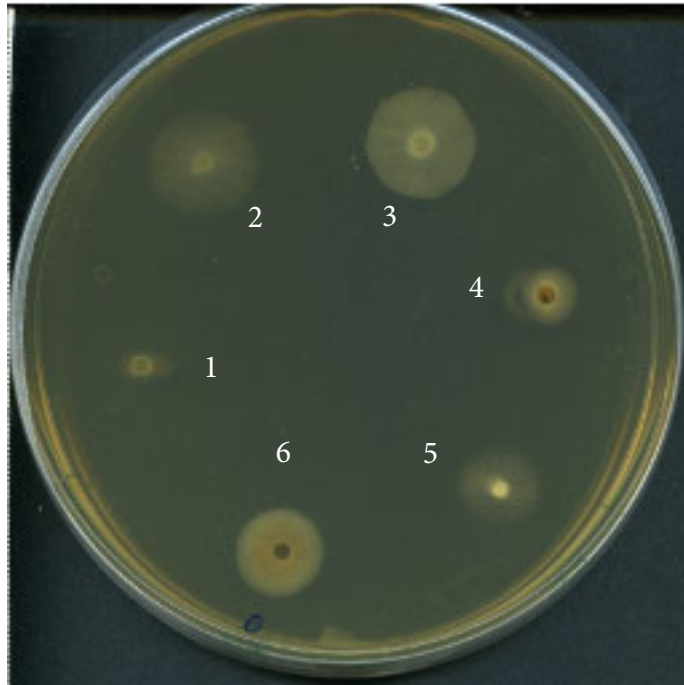

1. *H. annosum*
2. *B. adusta* (m)
3. *Diaporthe* sp.
4. *Annulohypoxylon* sp.
5. *B. adusta* (d)
6. *A. arborescens*

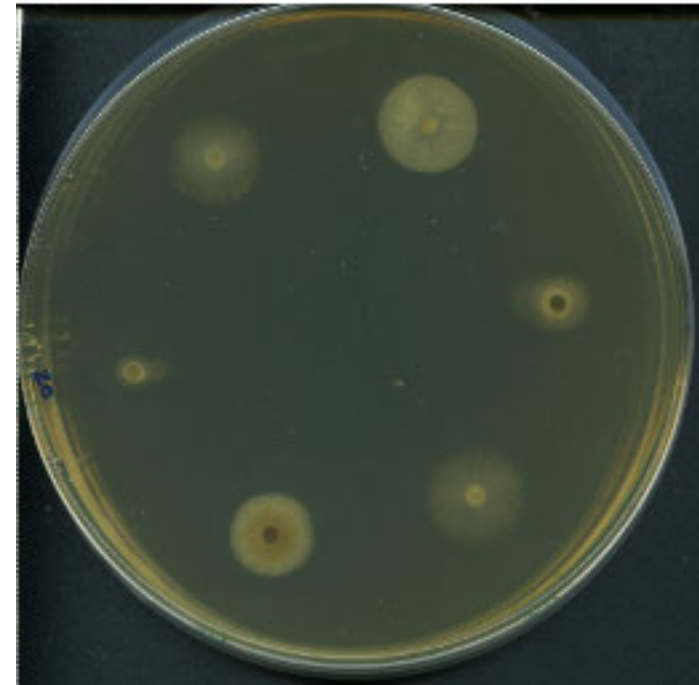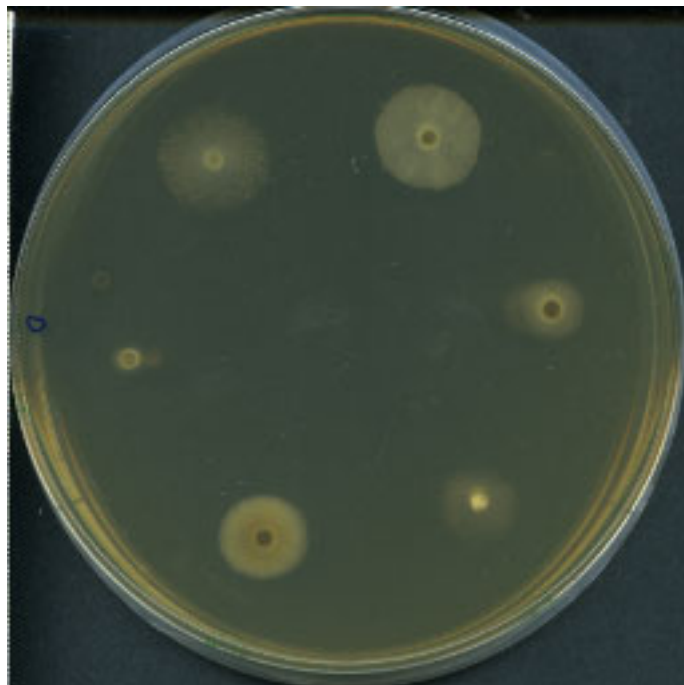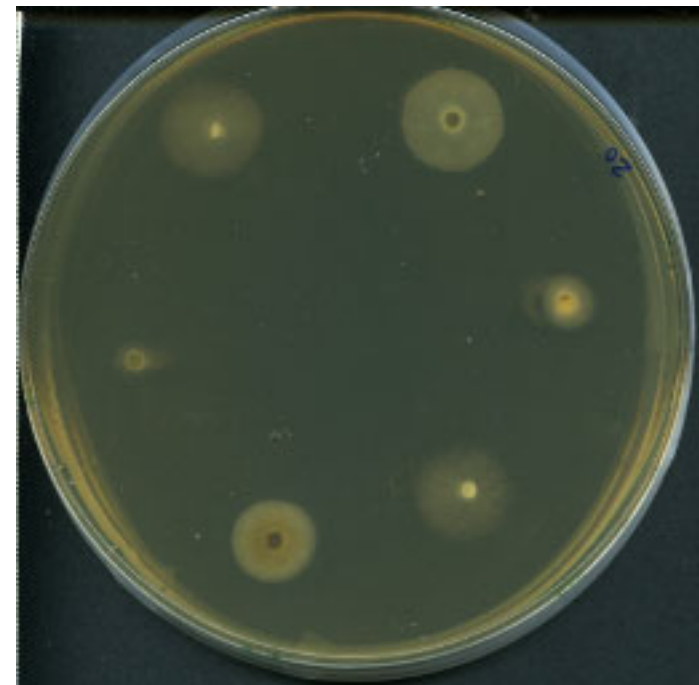

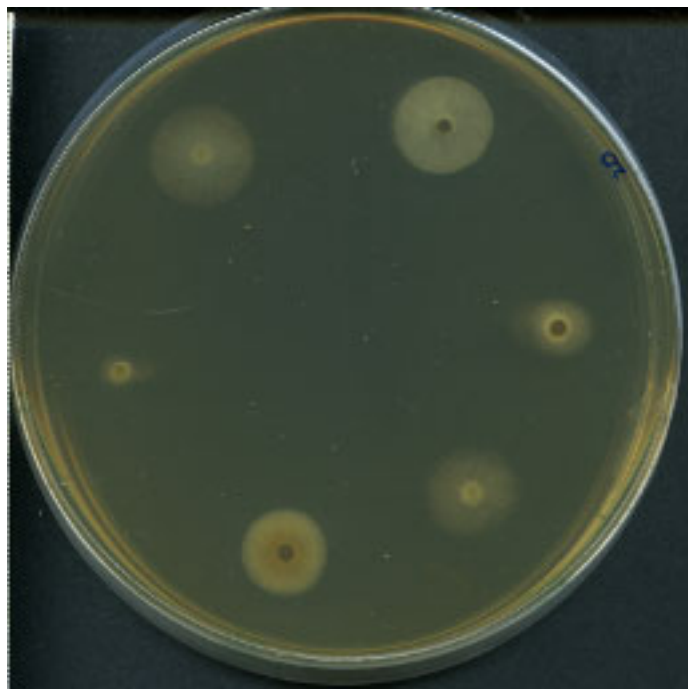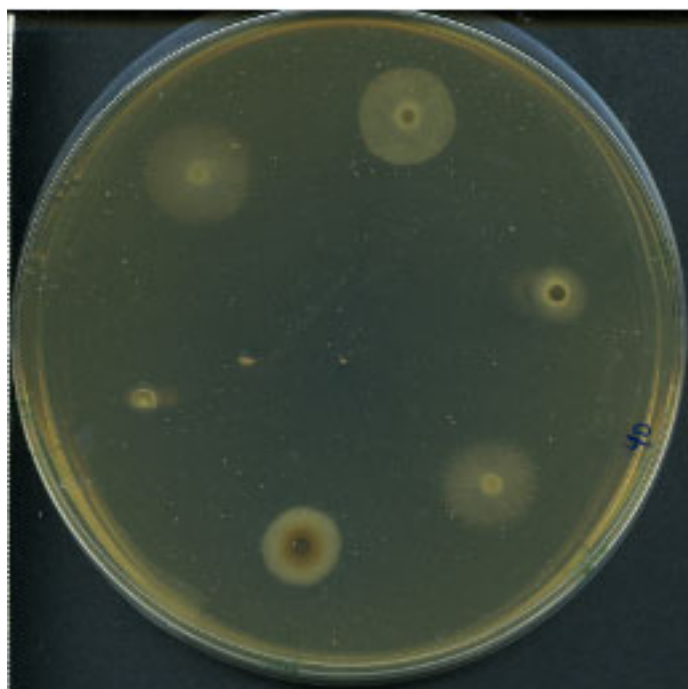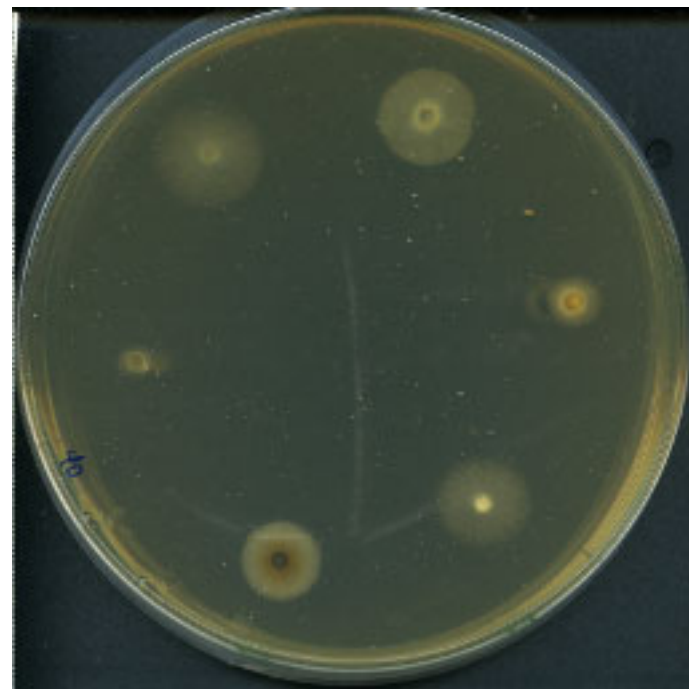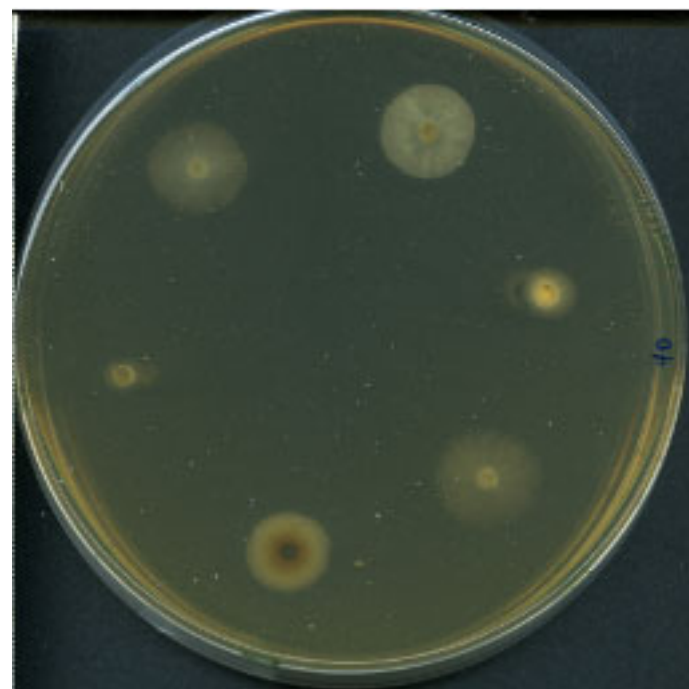

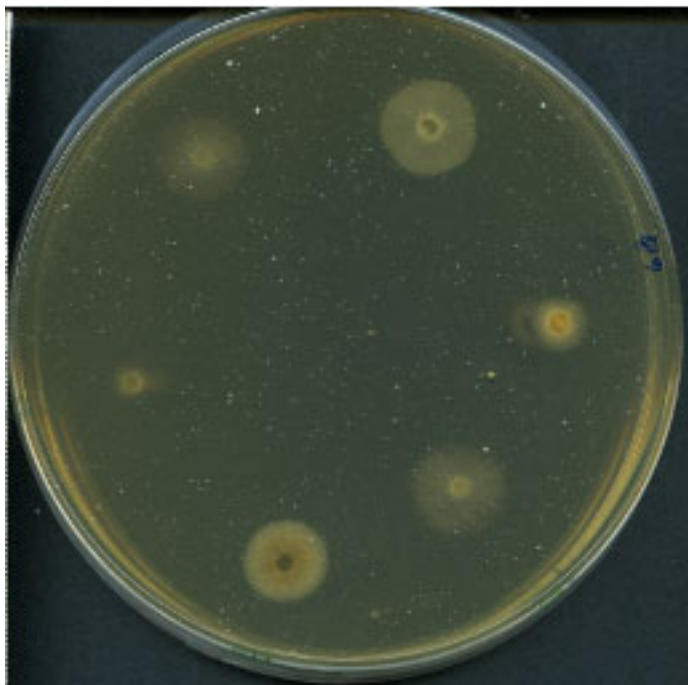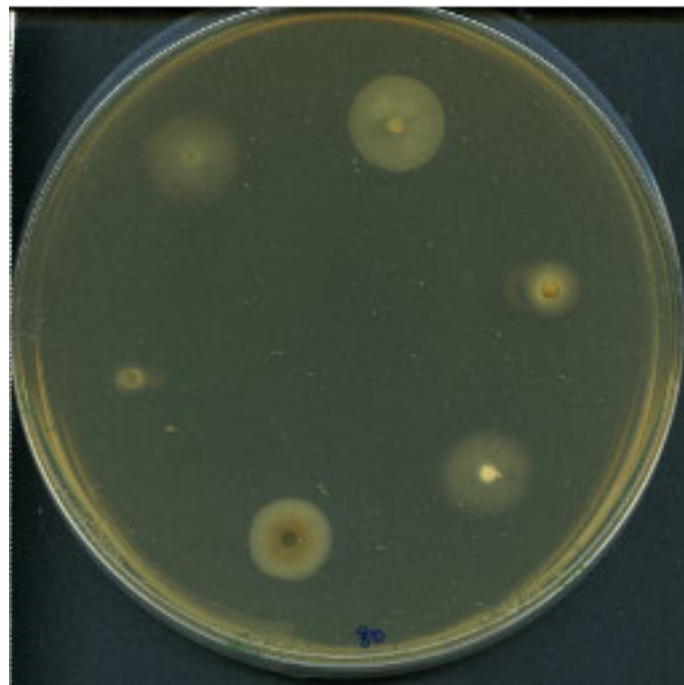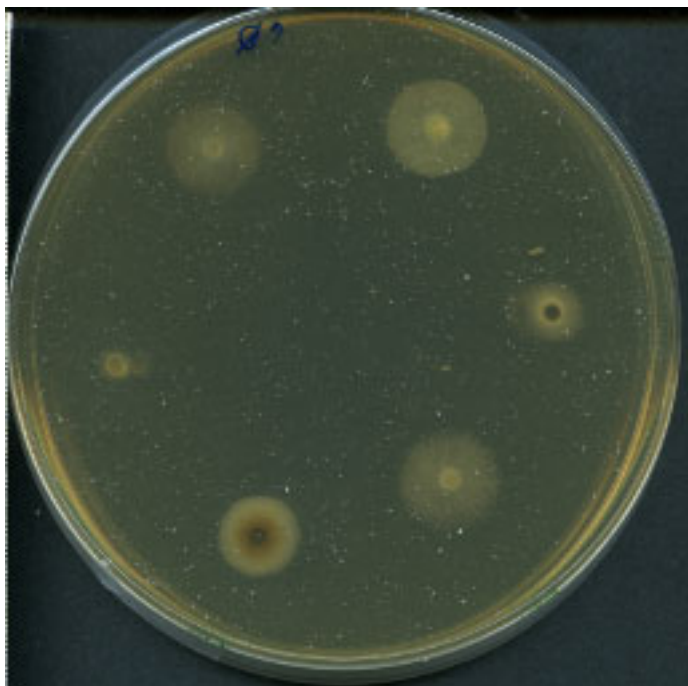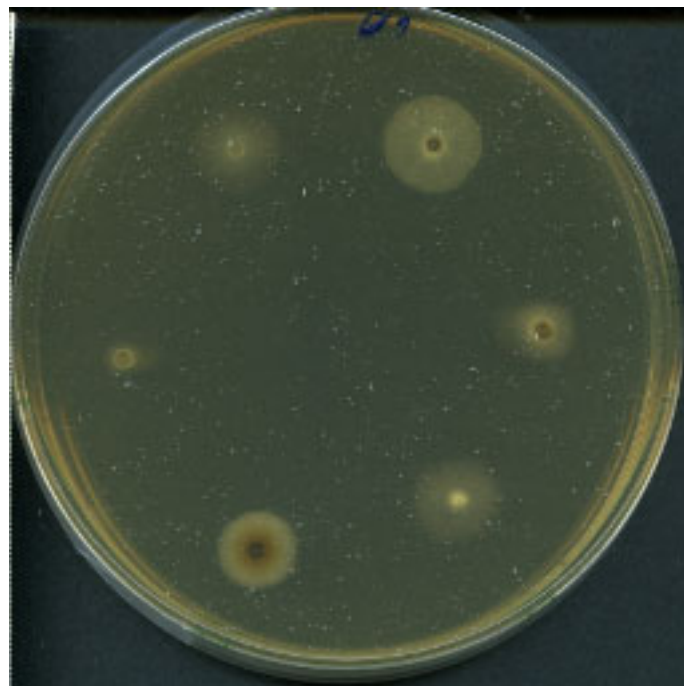

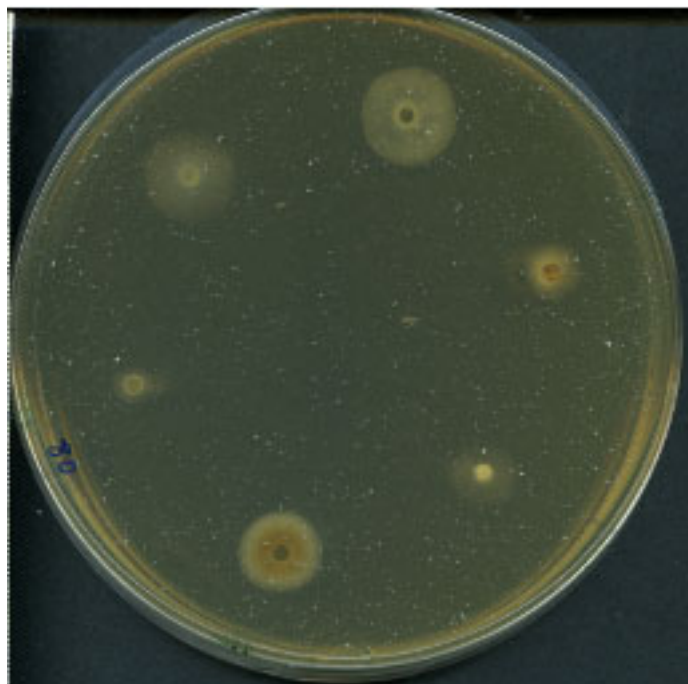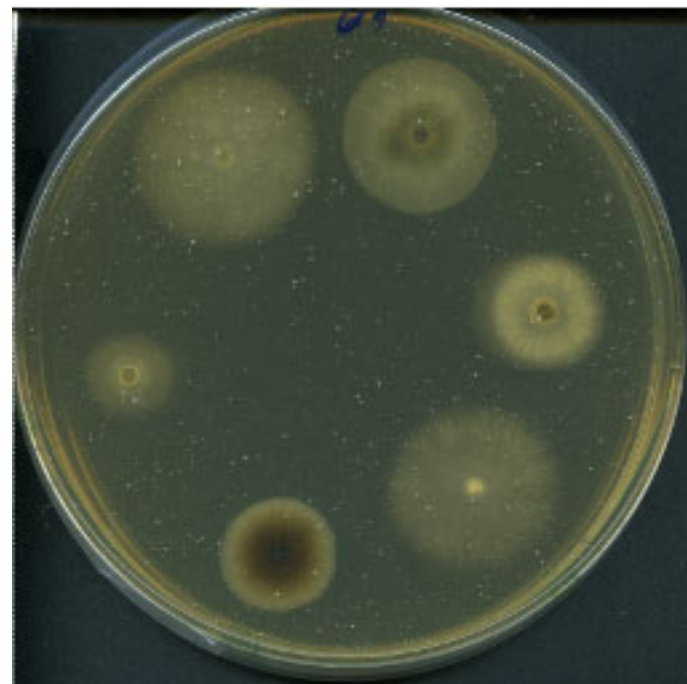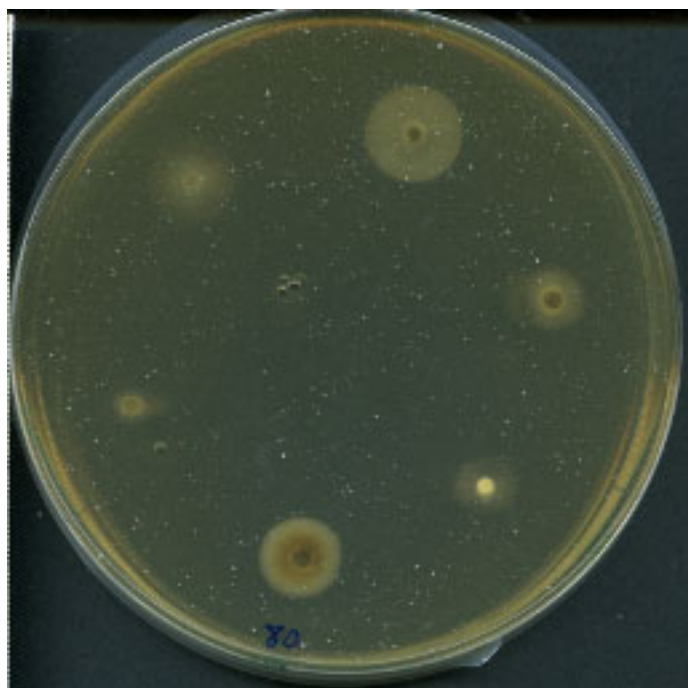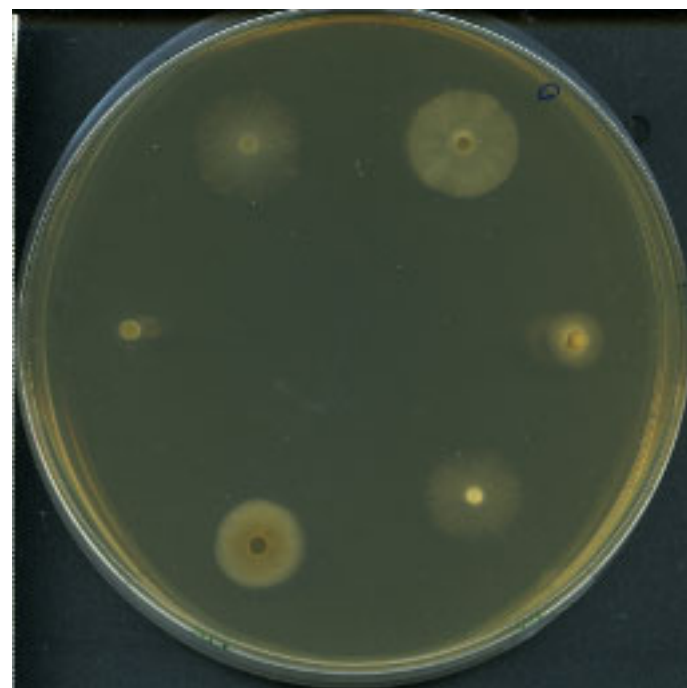

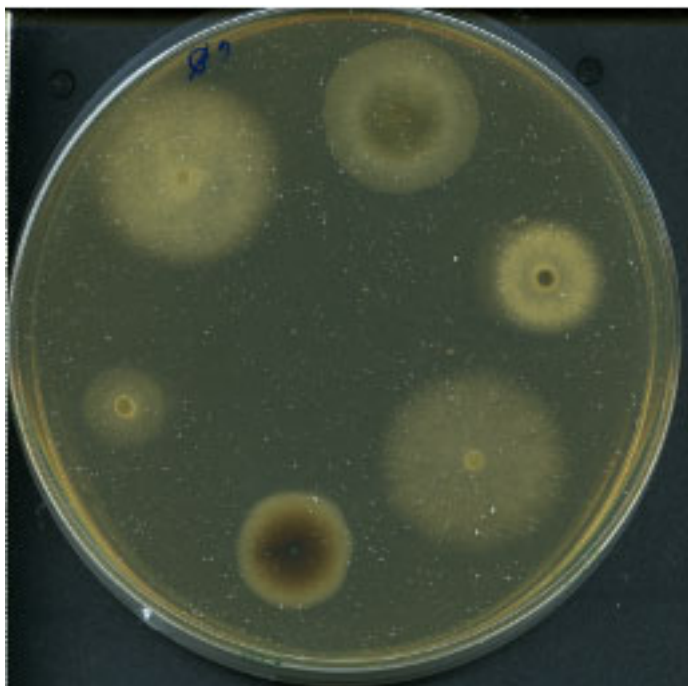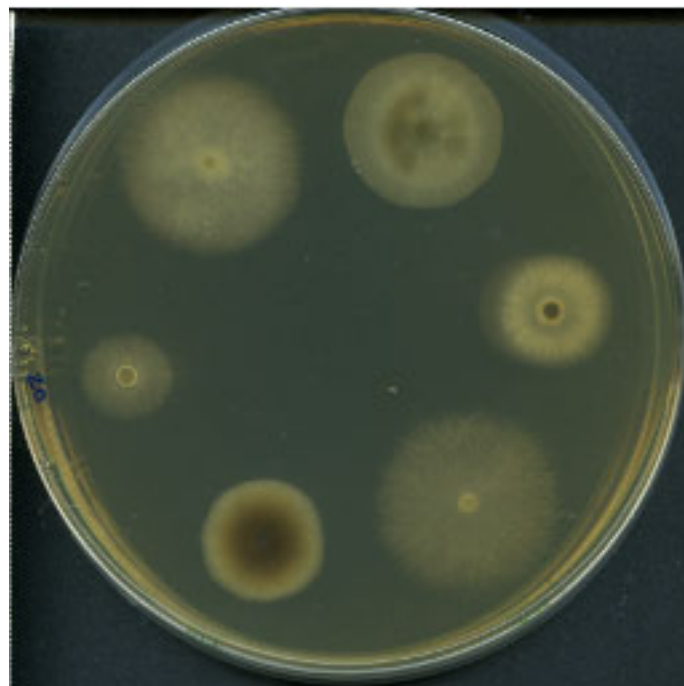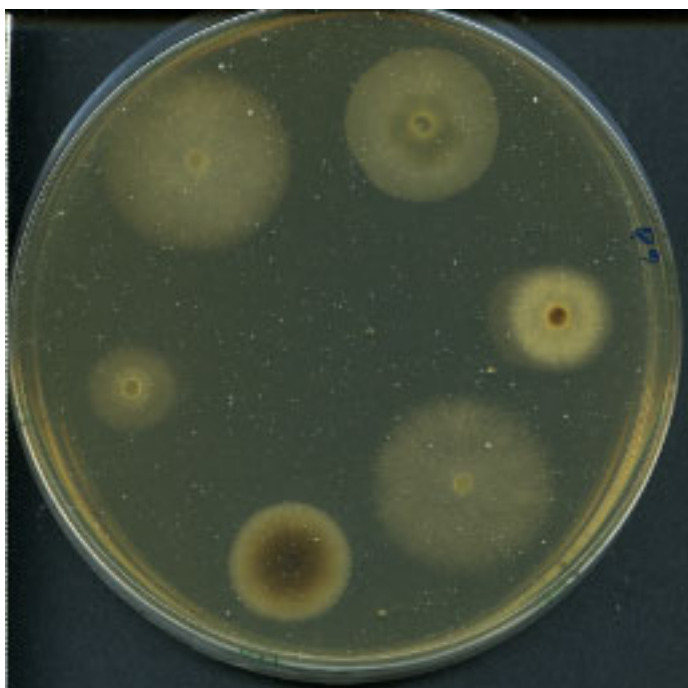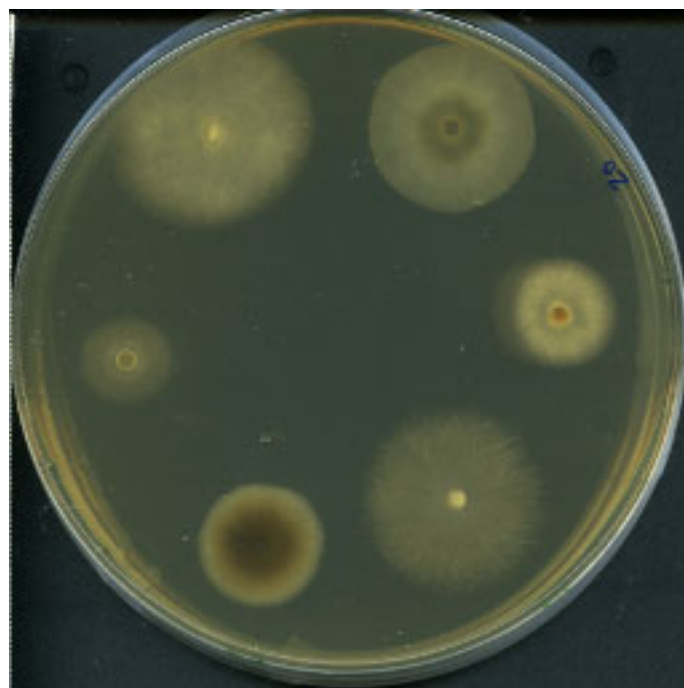

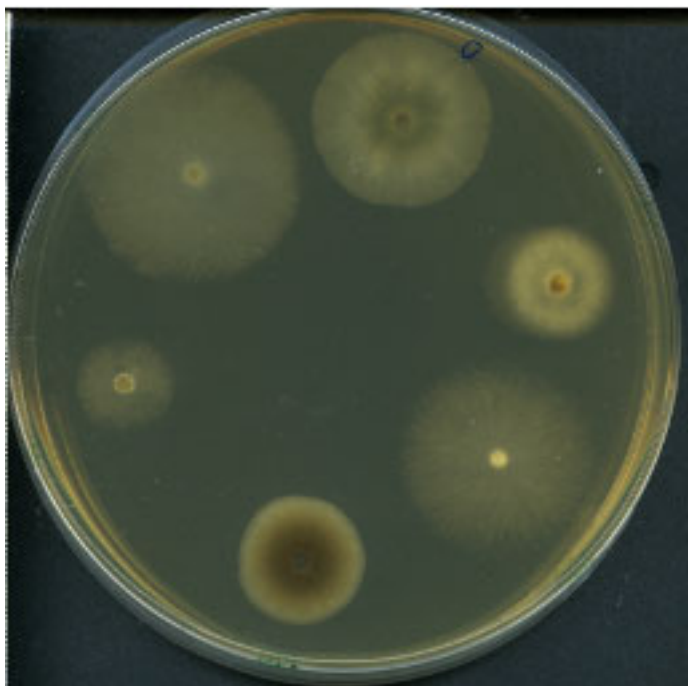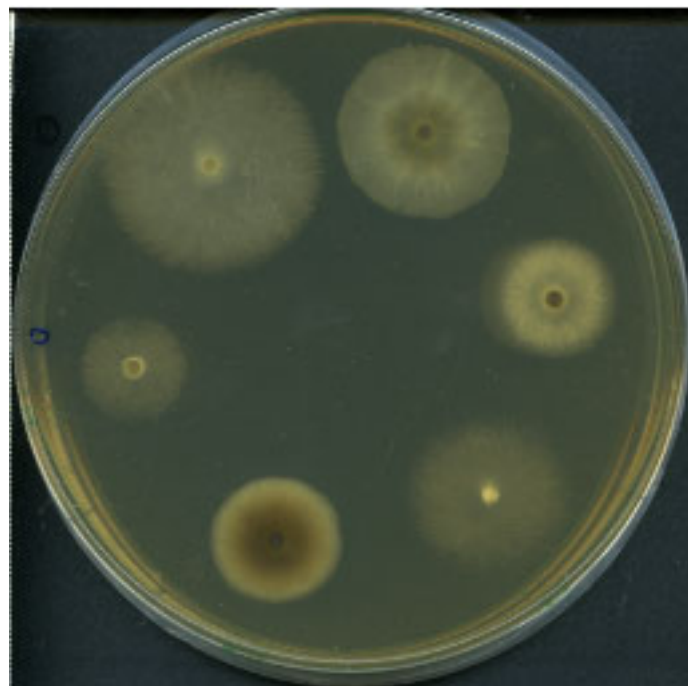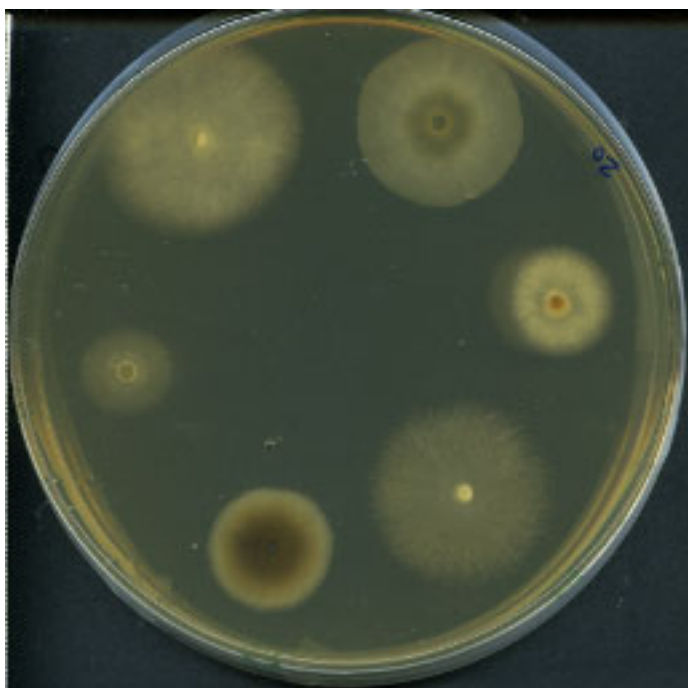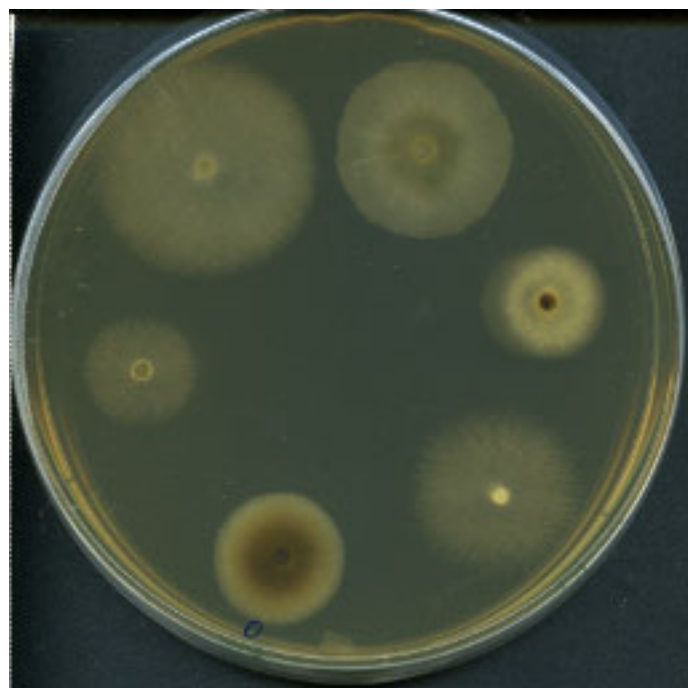

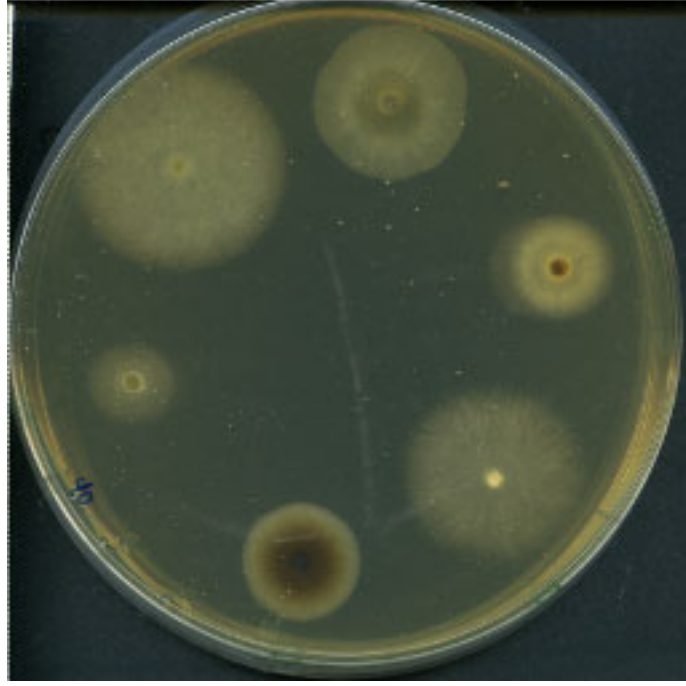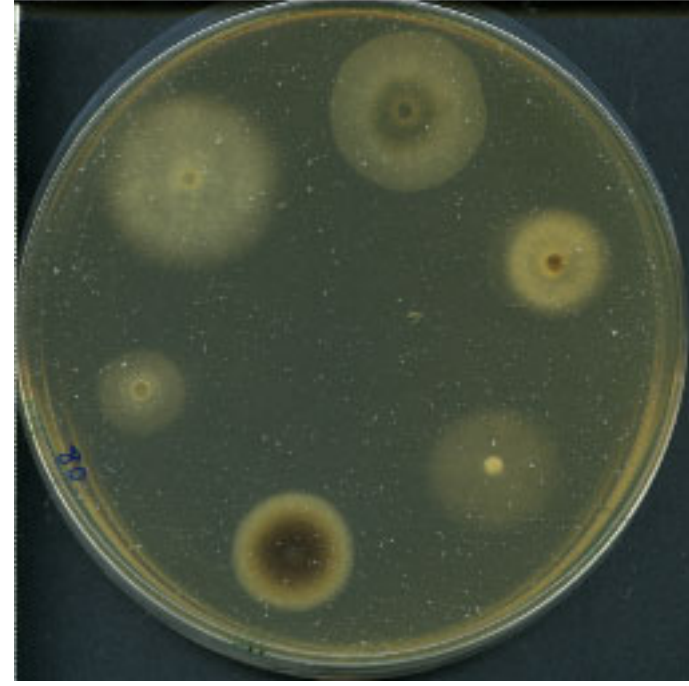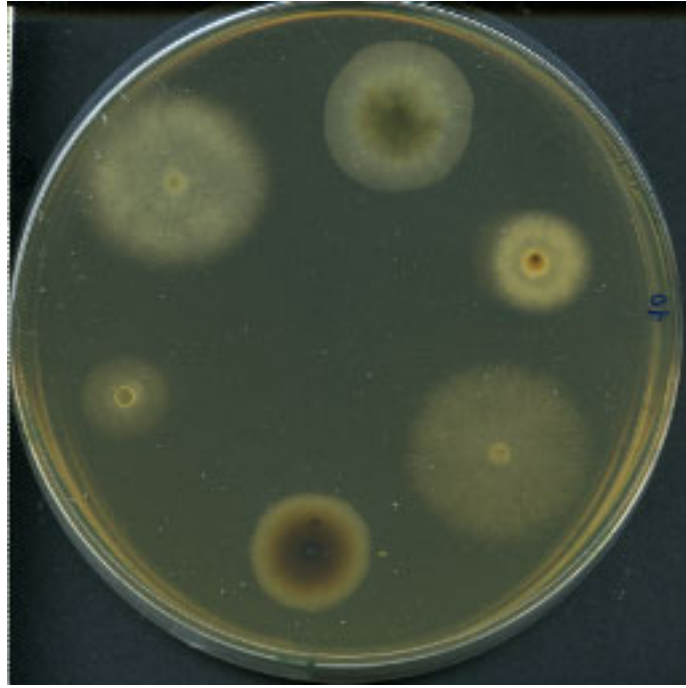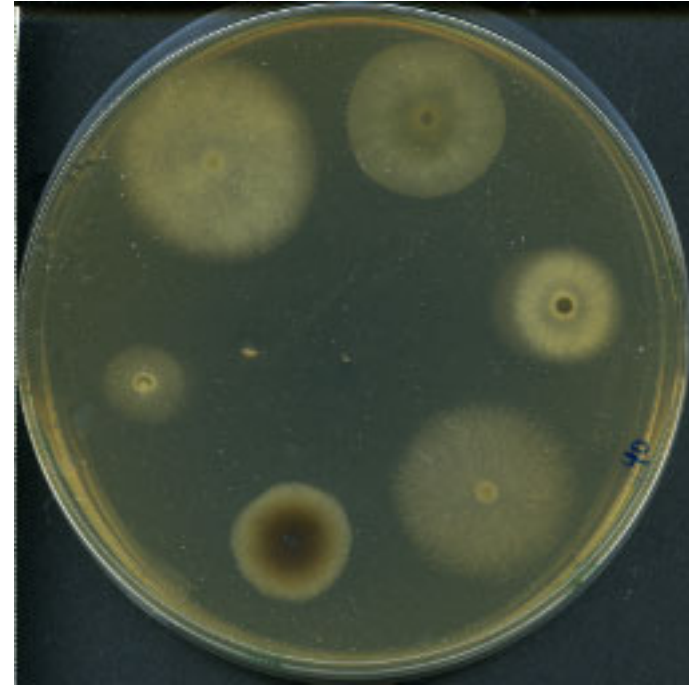

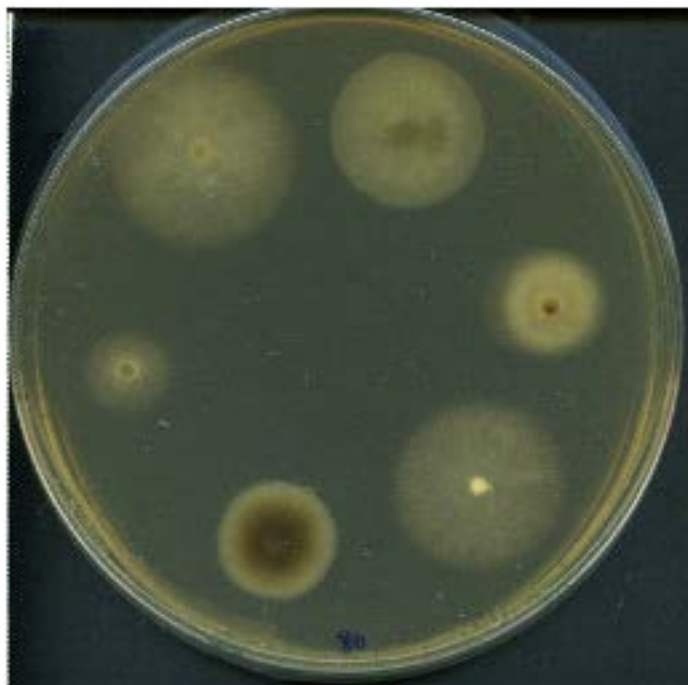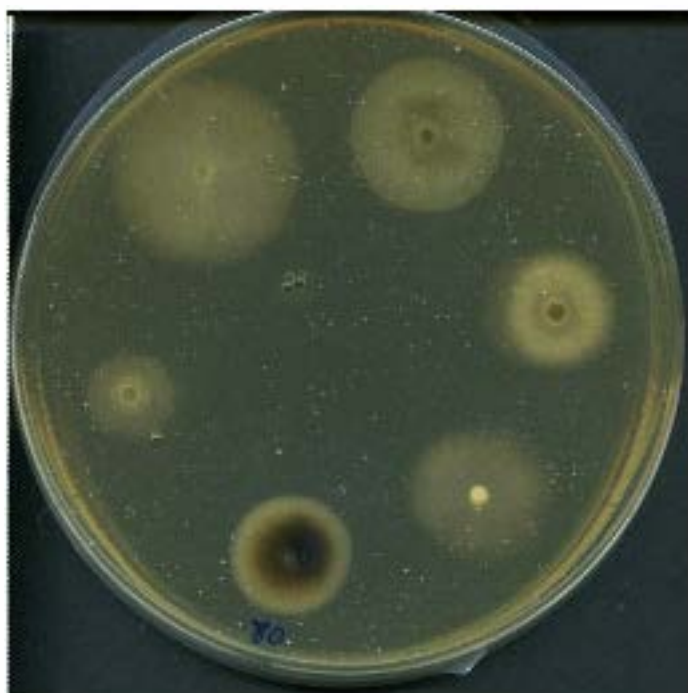

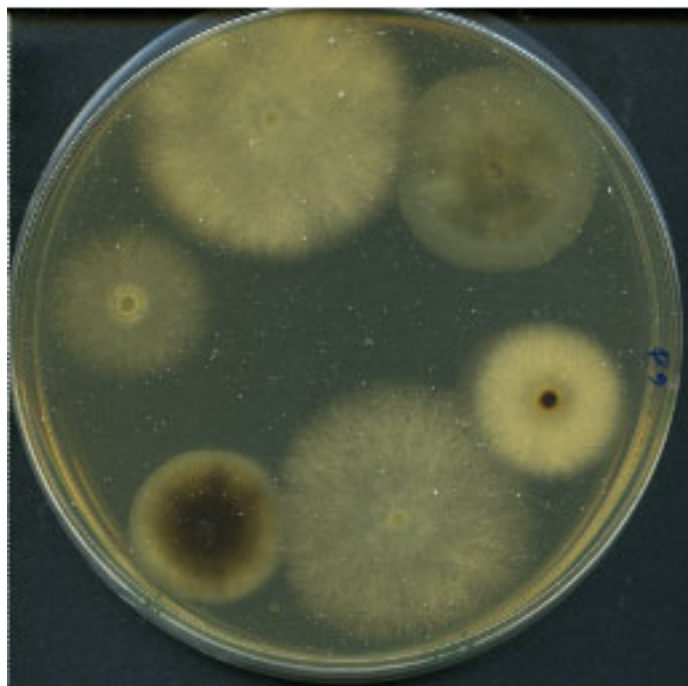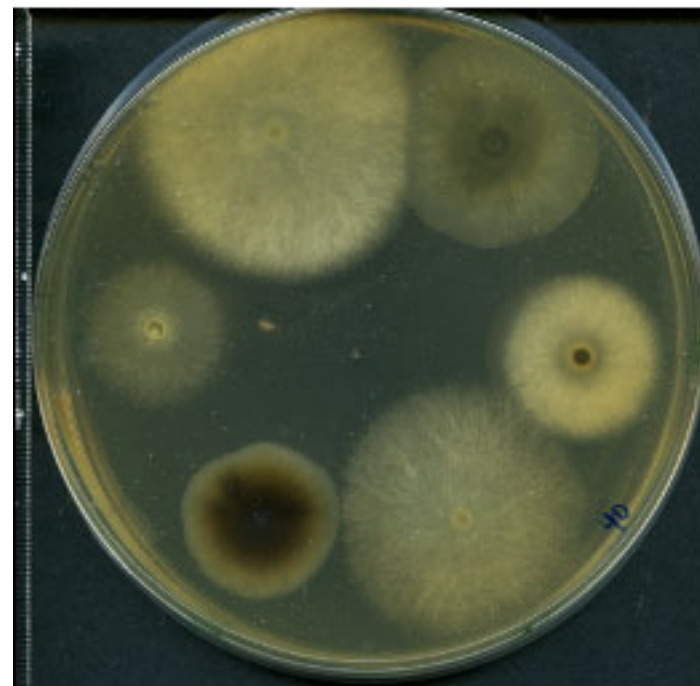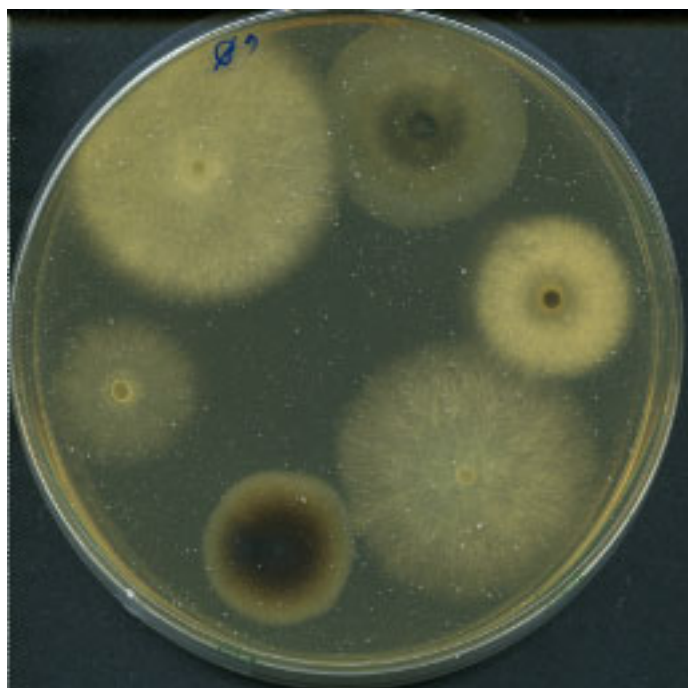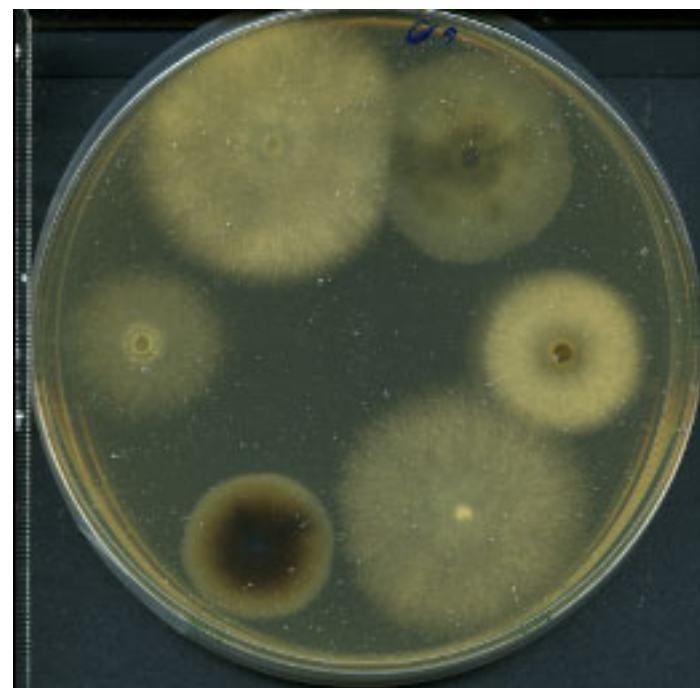

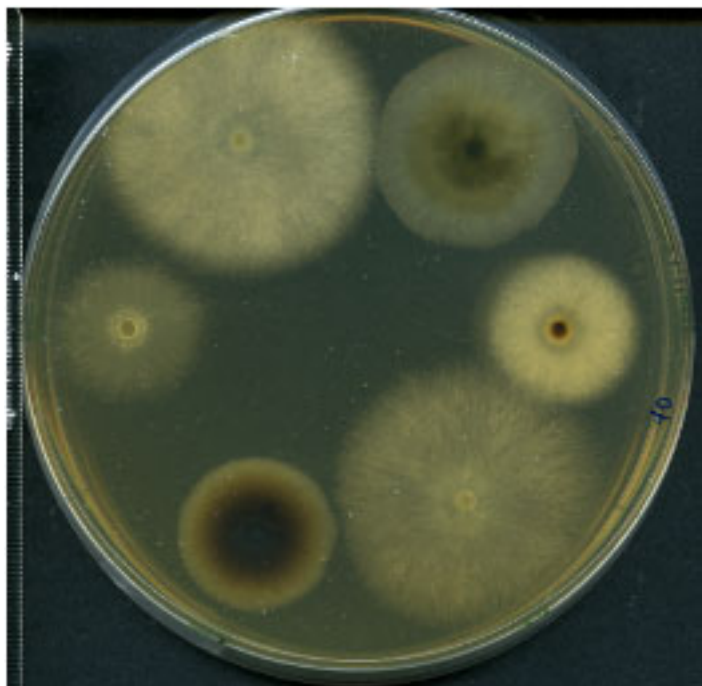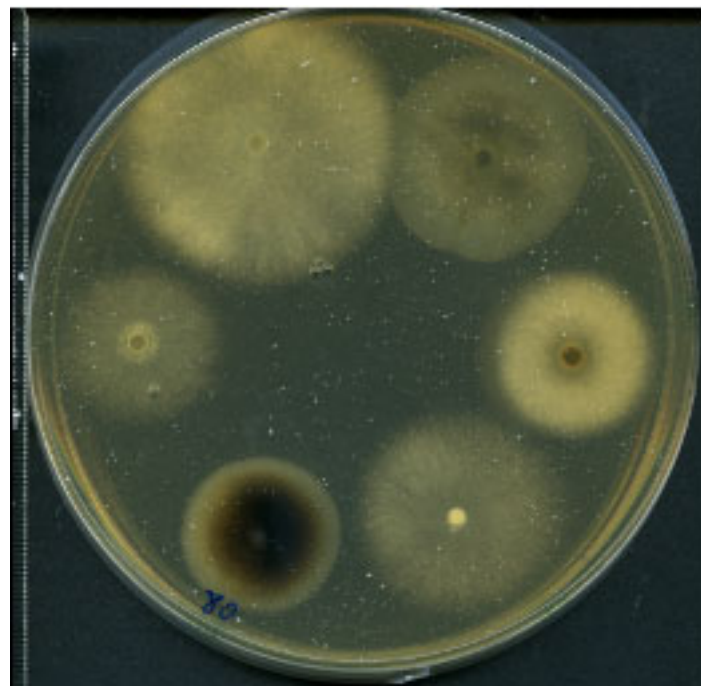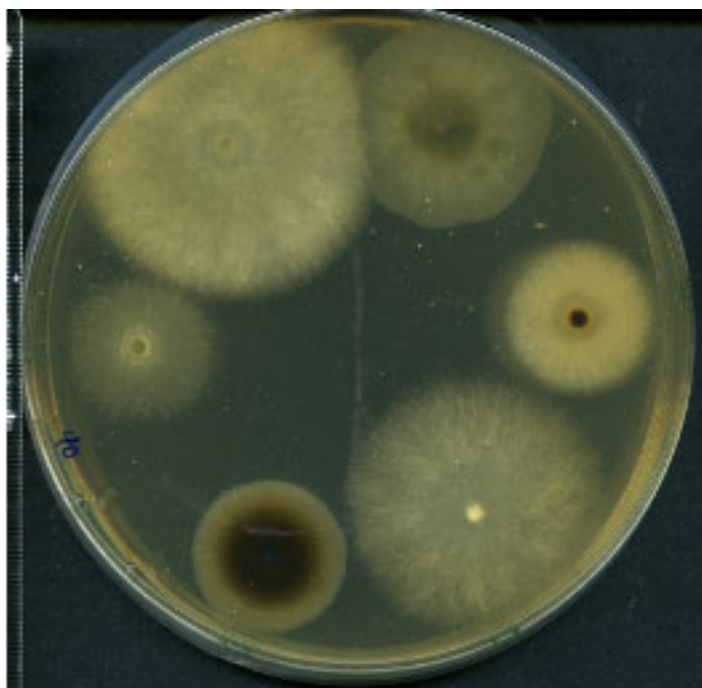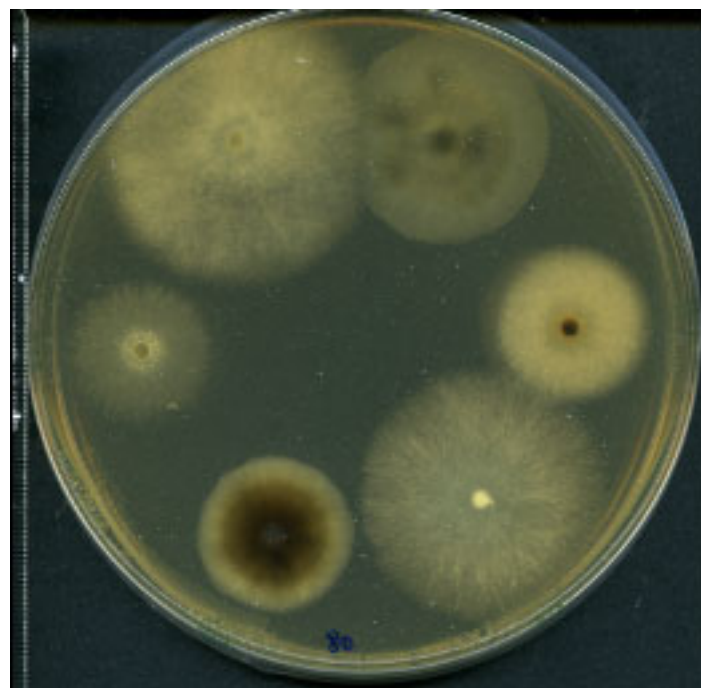

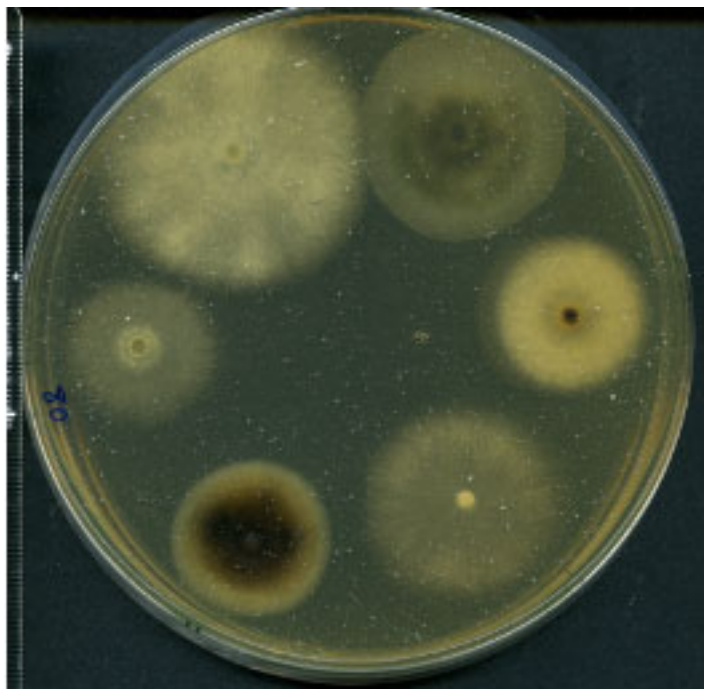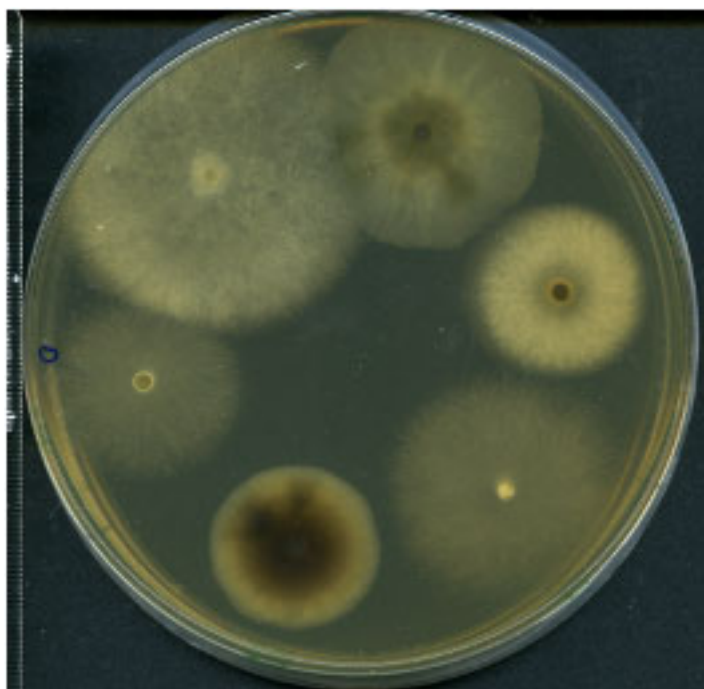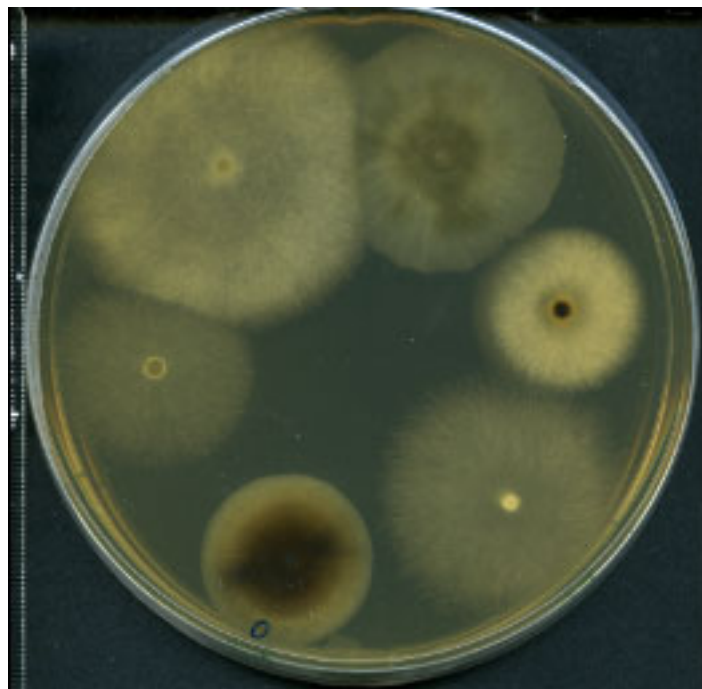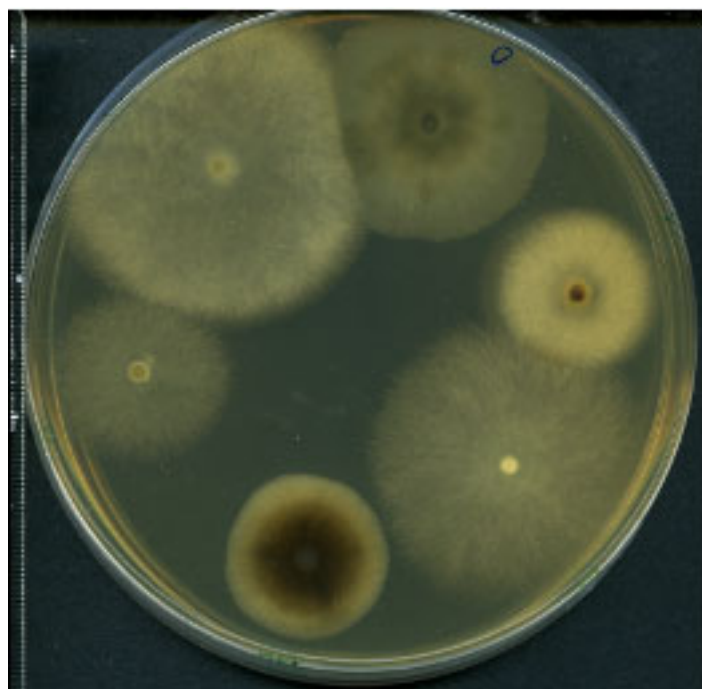

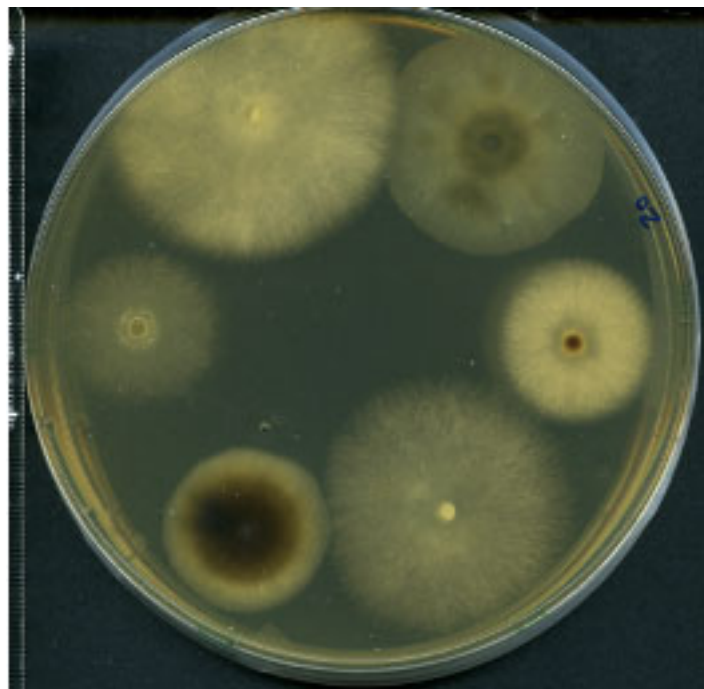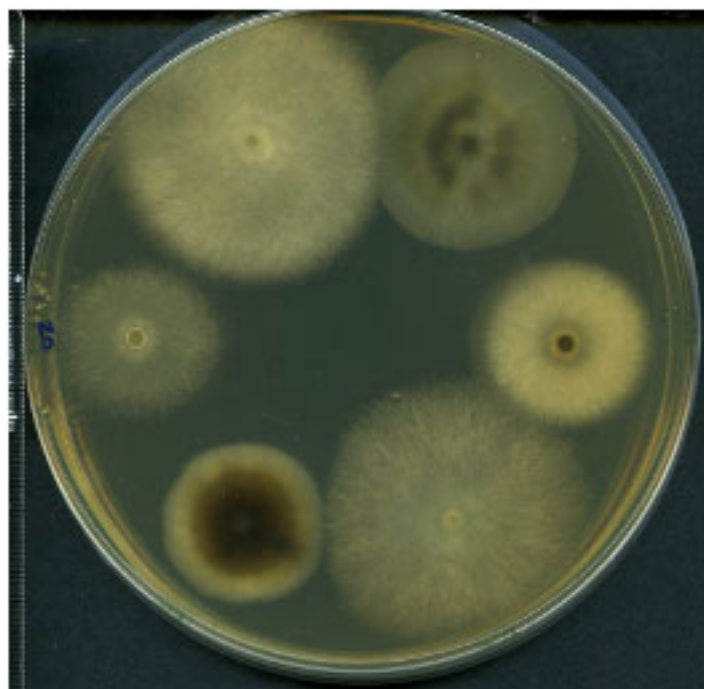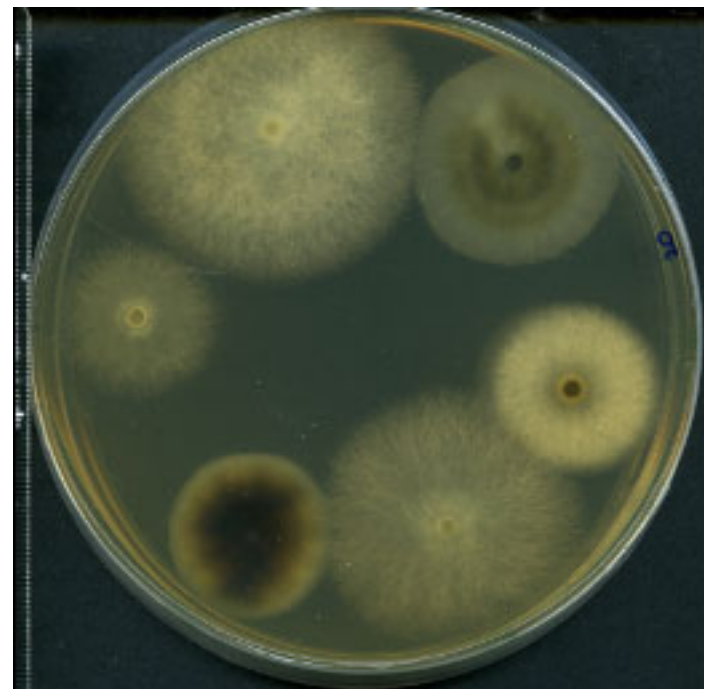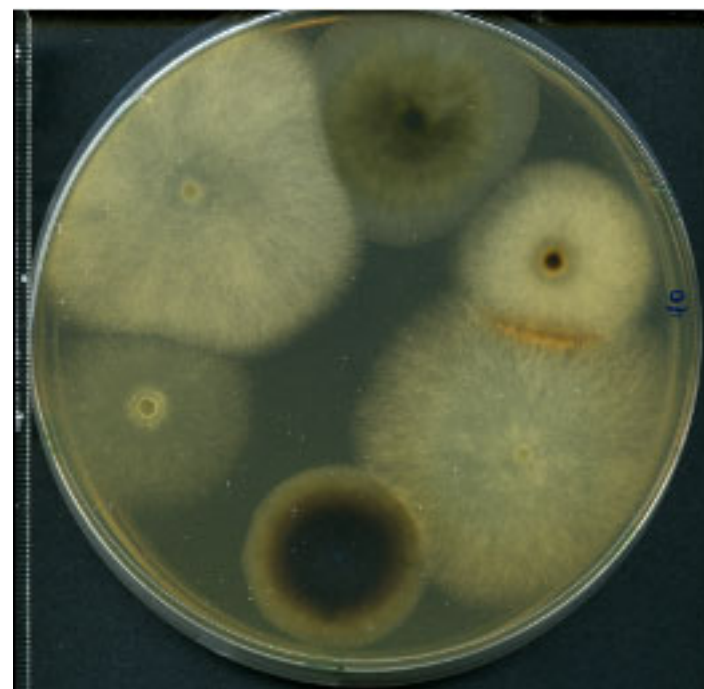

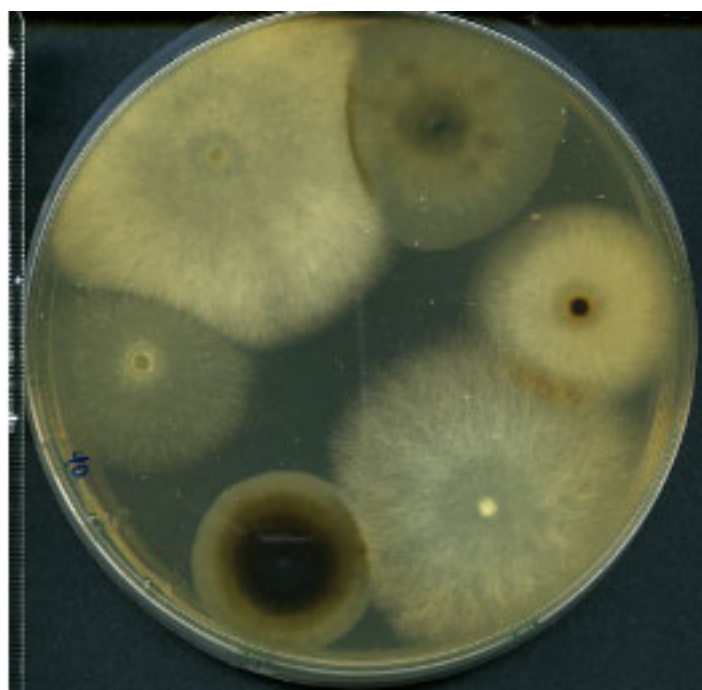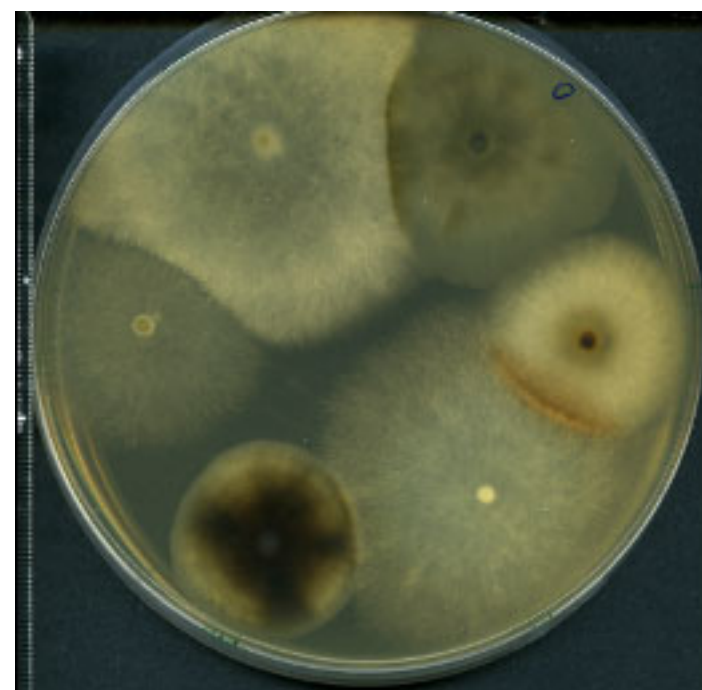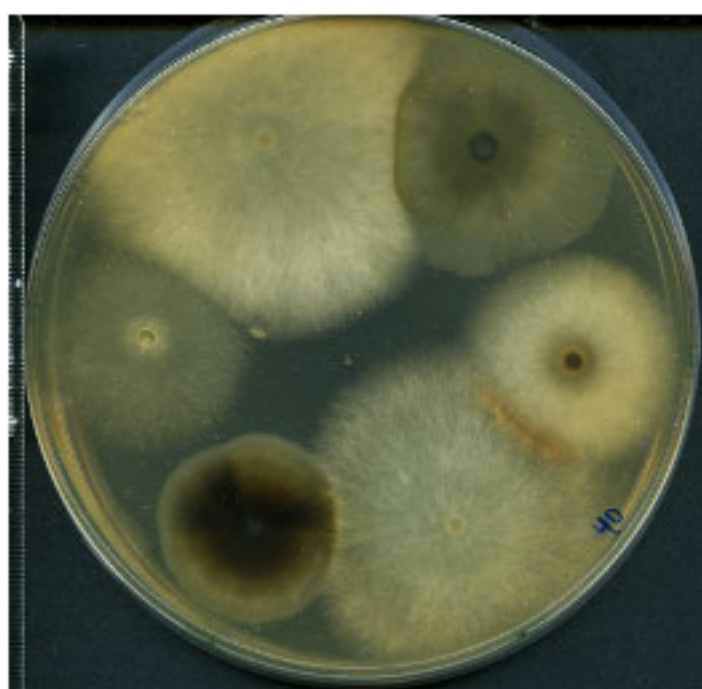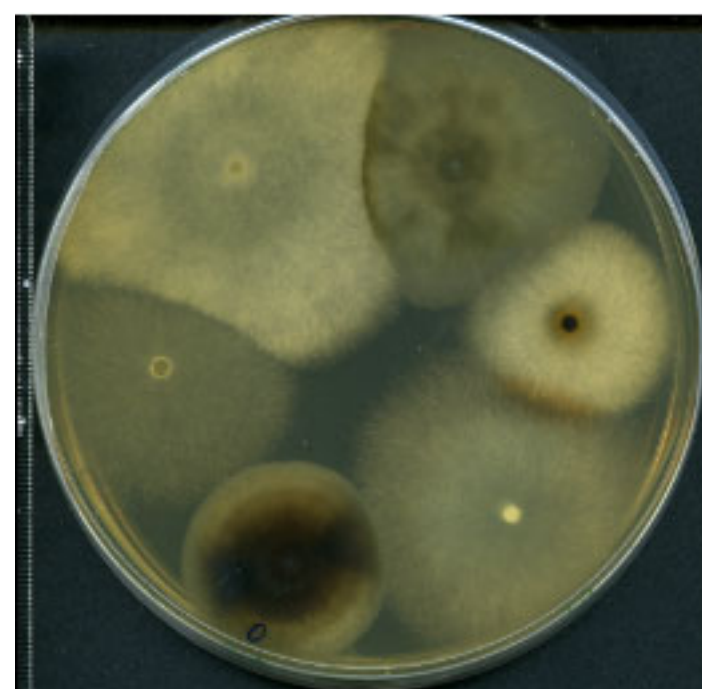

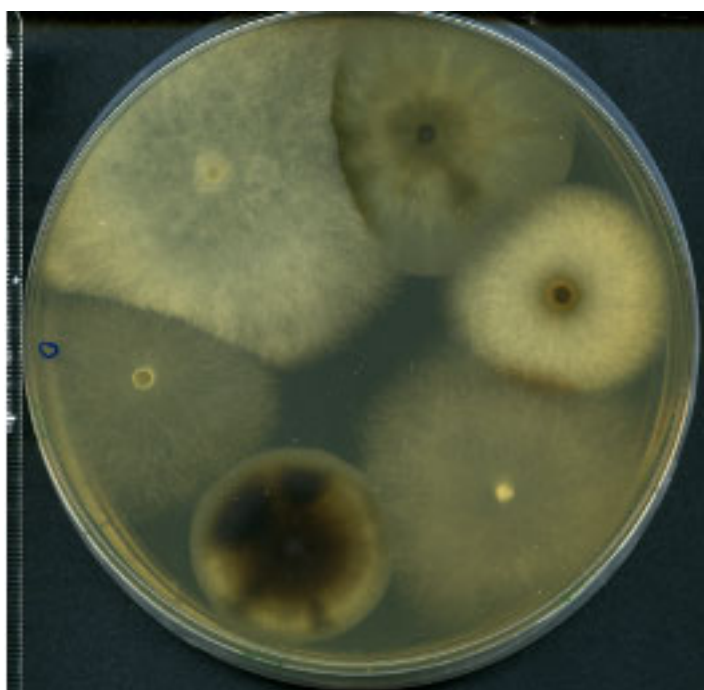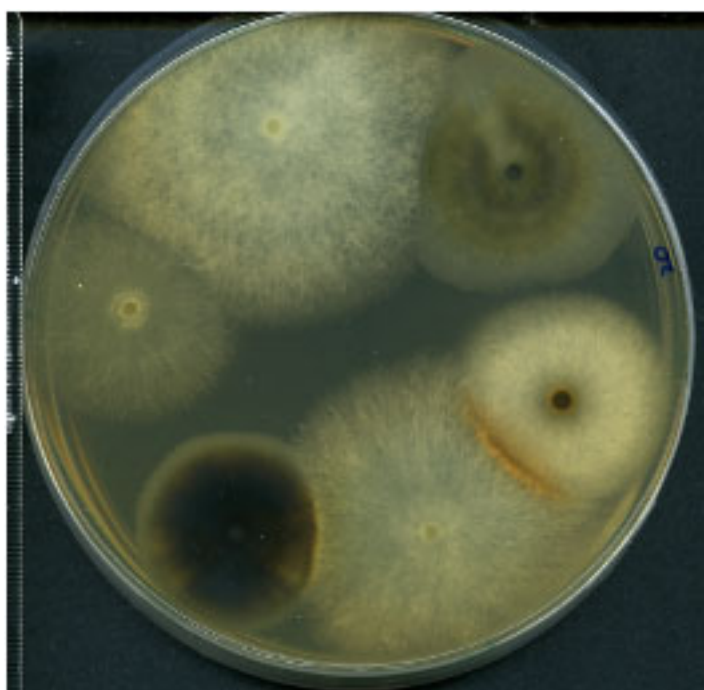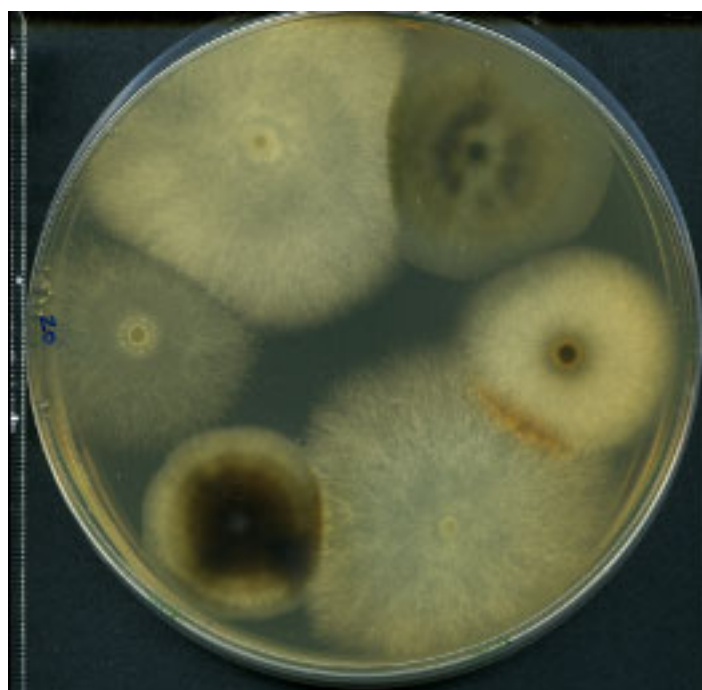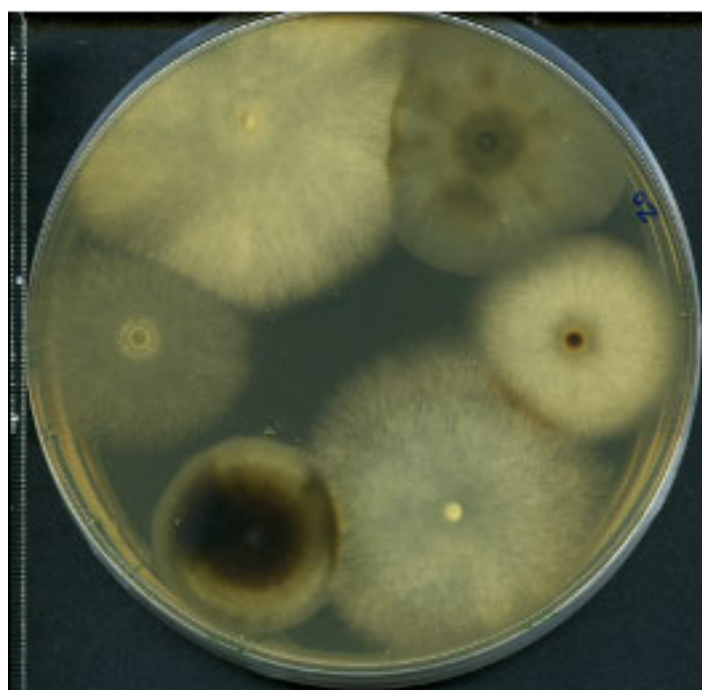

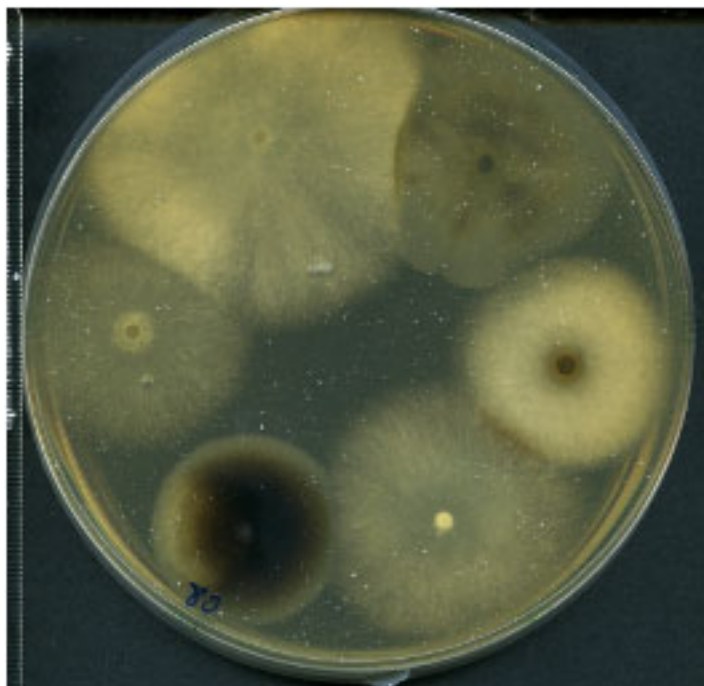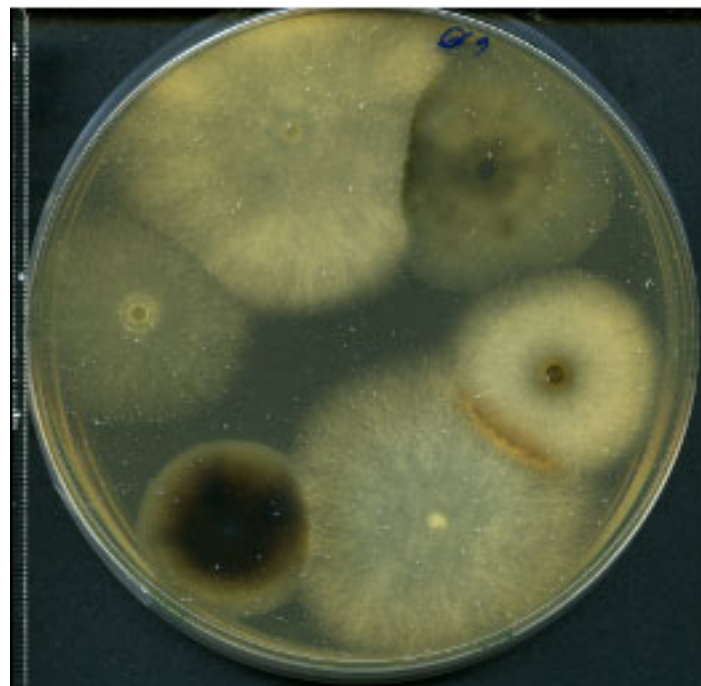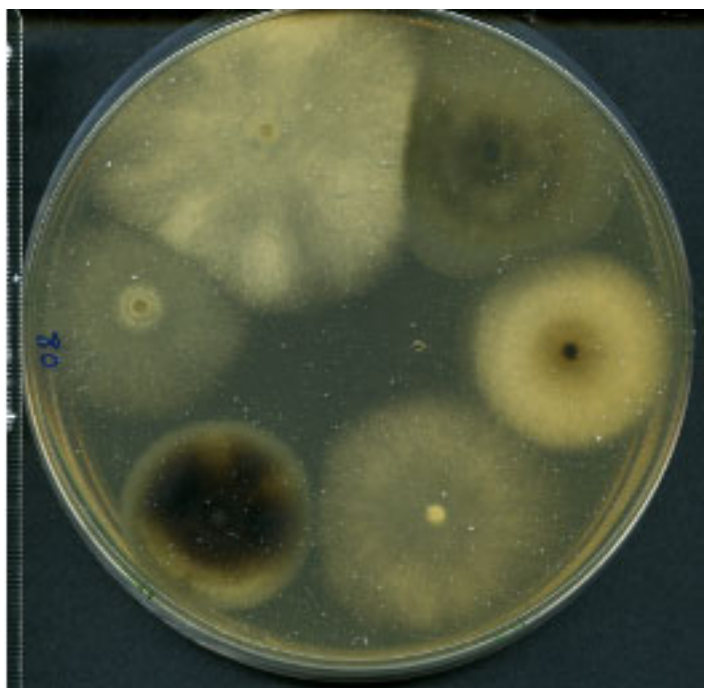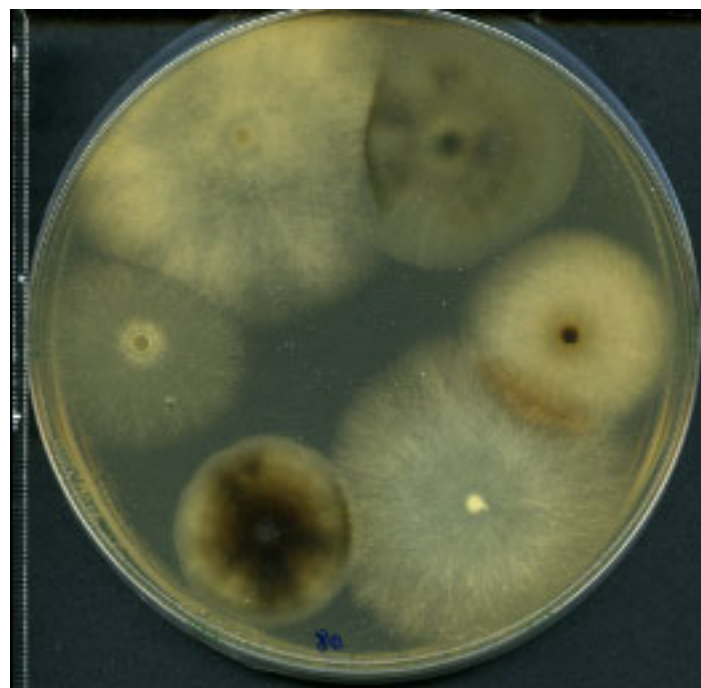

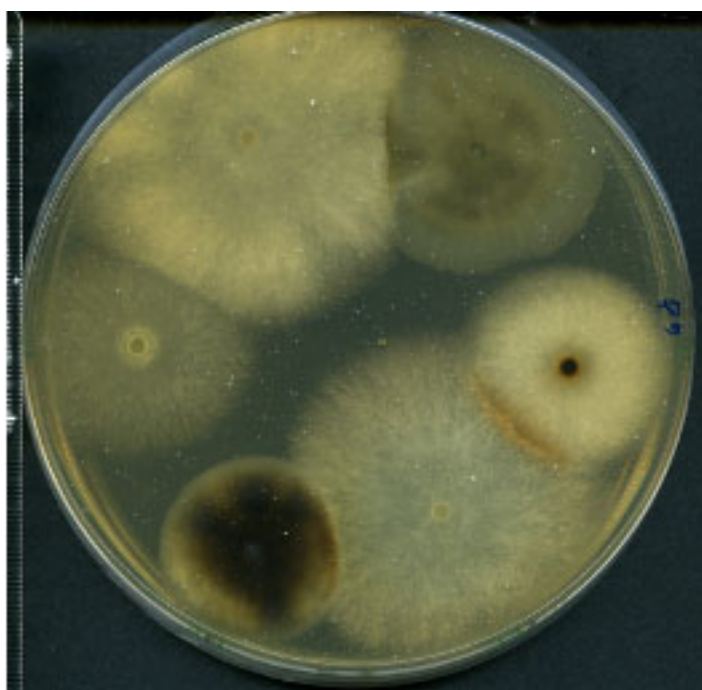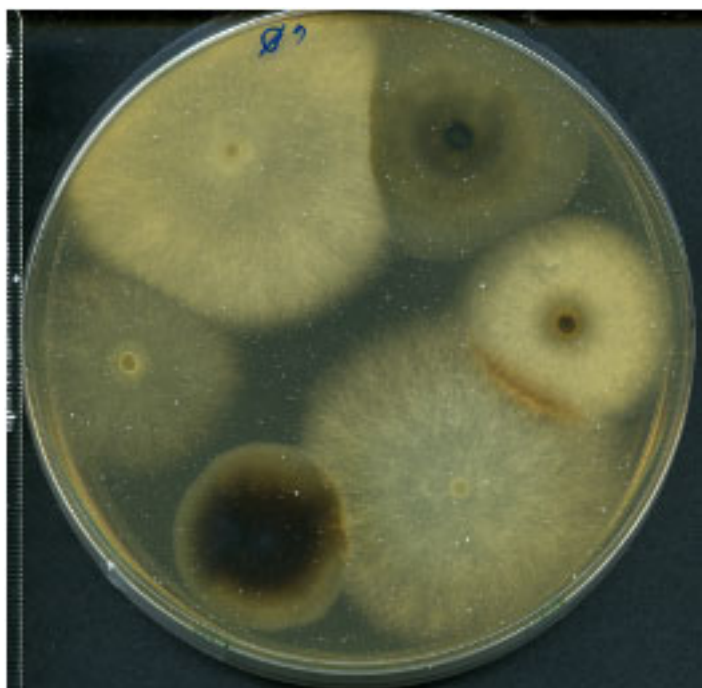

Supplement: Supplementary file 2 [file Data_Sheet_2.zip › Raw images_MMN growth Assays.pdf]

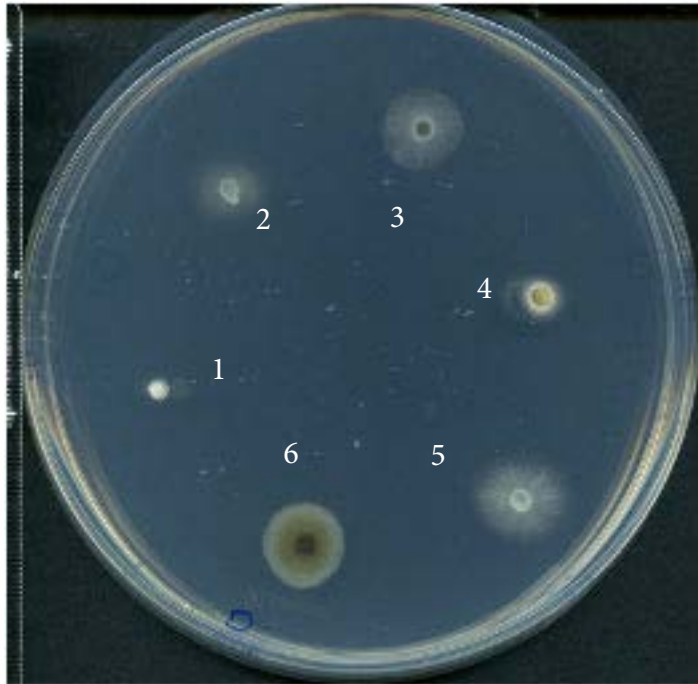

1. *H. annosum*
2. *B. adusta* (m)
3. *Diaporthe* sp.
4. *Annulohypoxyton* sp.
5. *B. adusta* (d)
6. *A. arborescens*

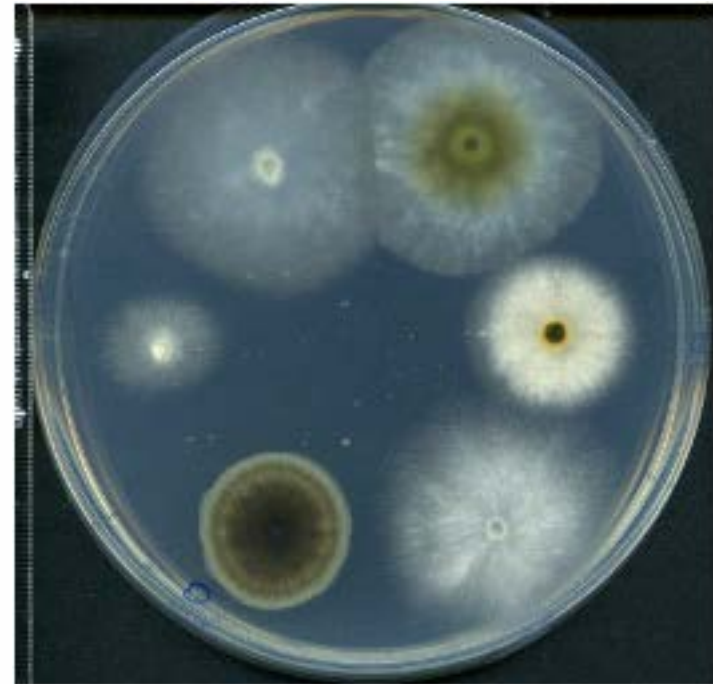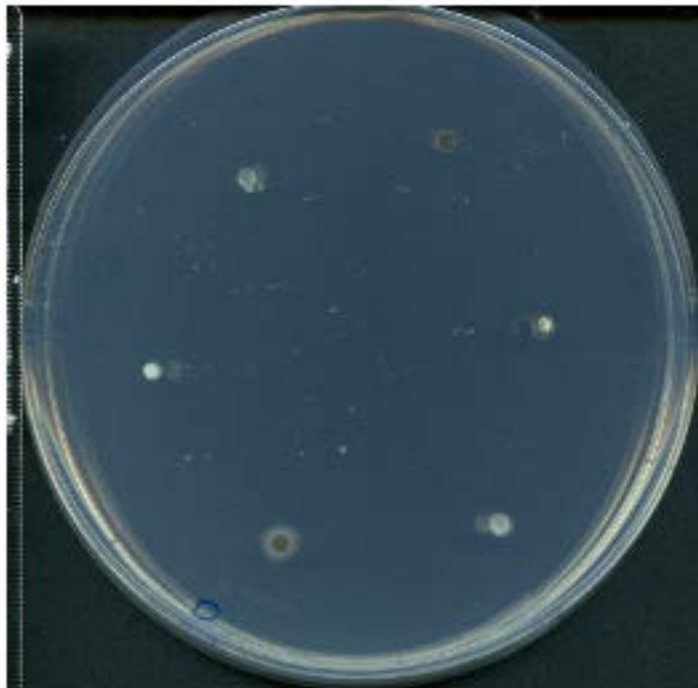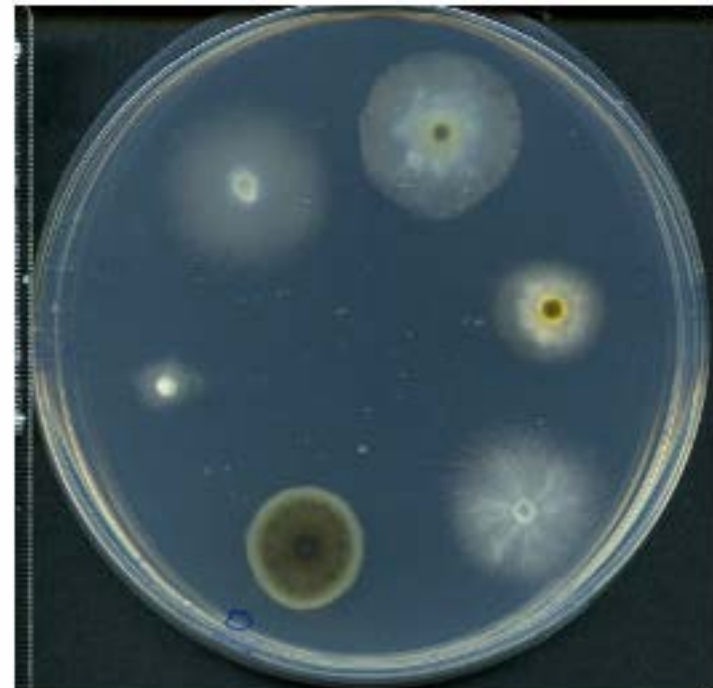

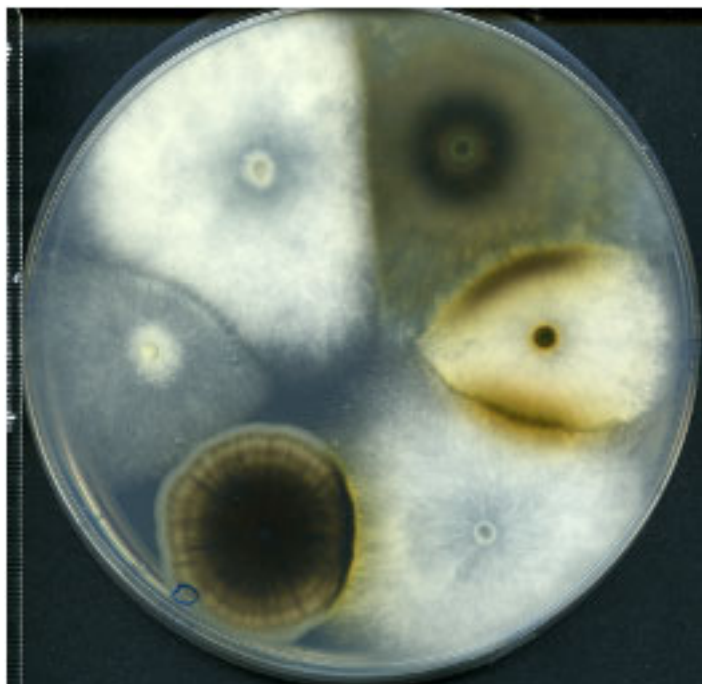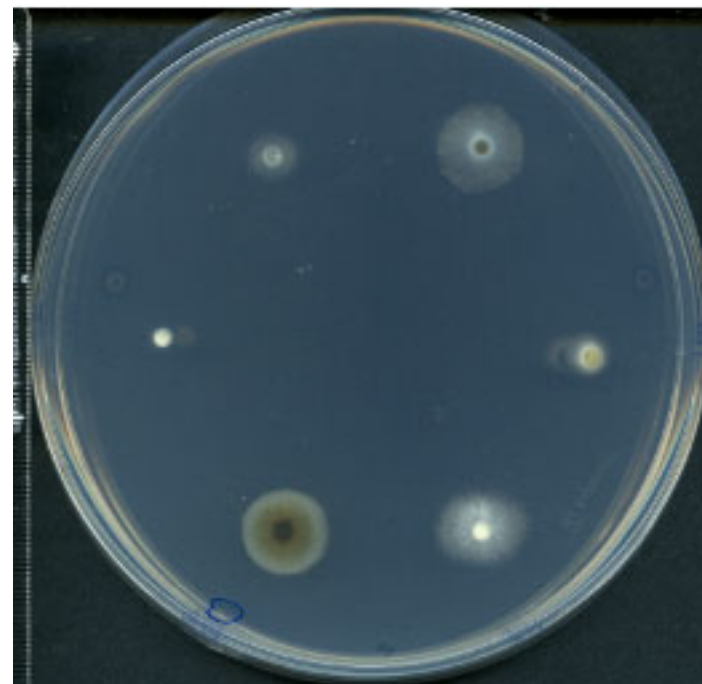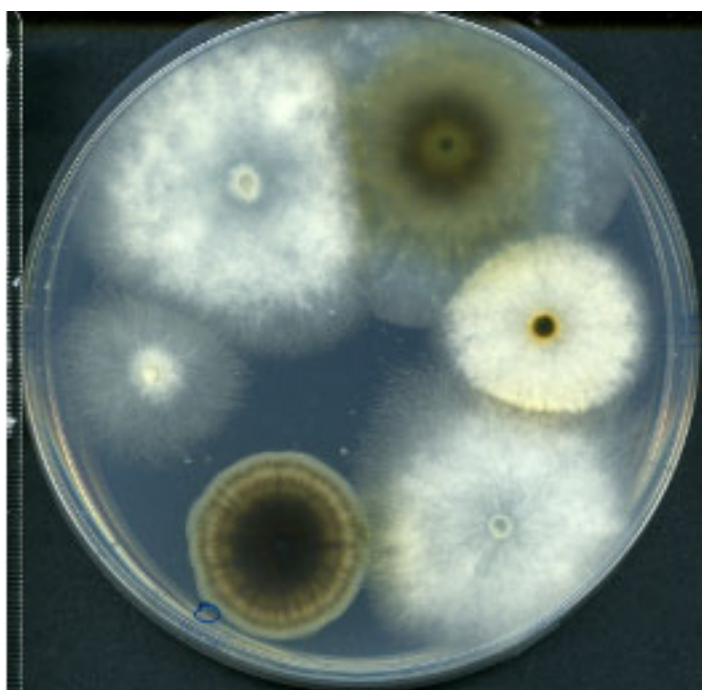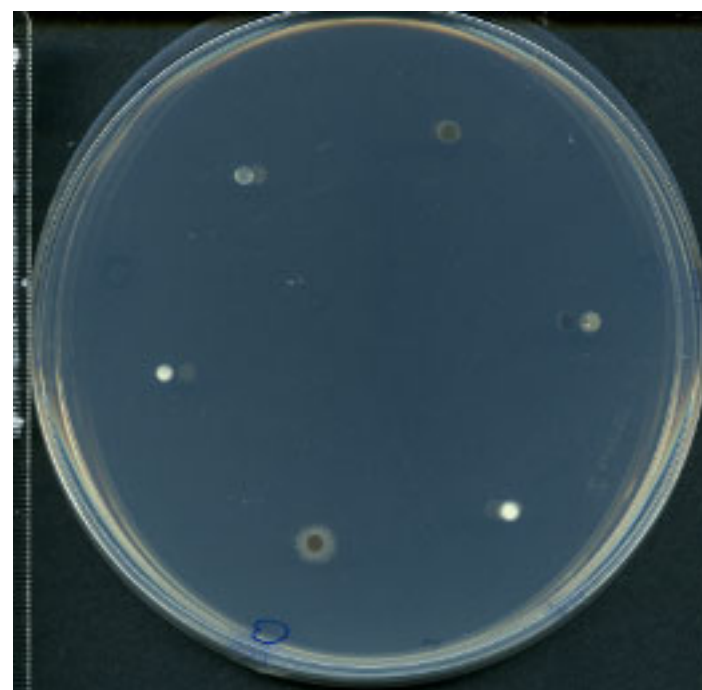

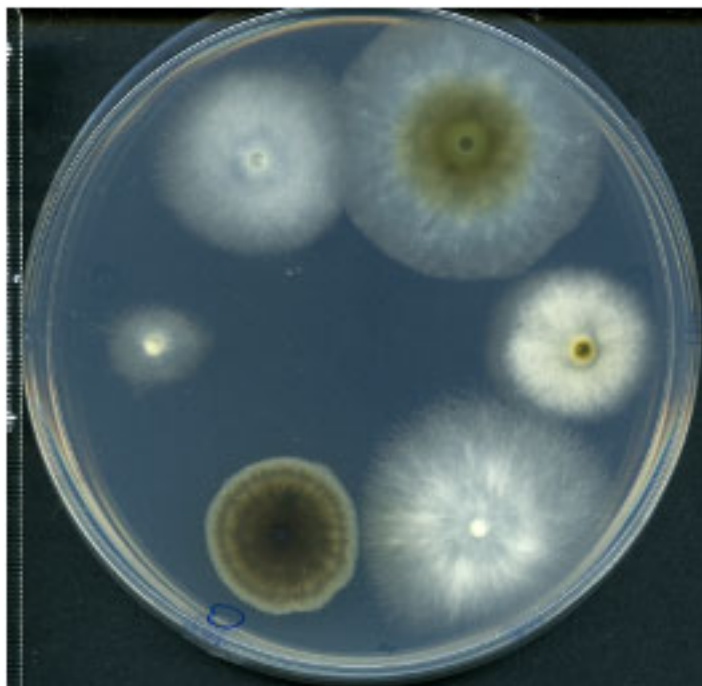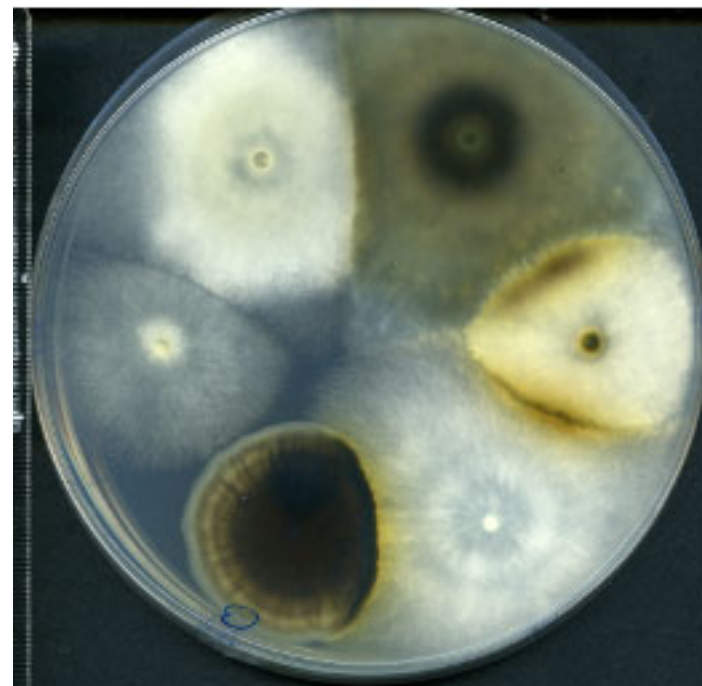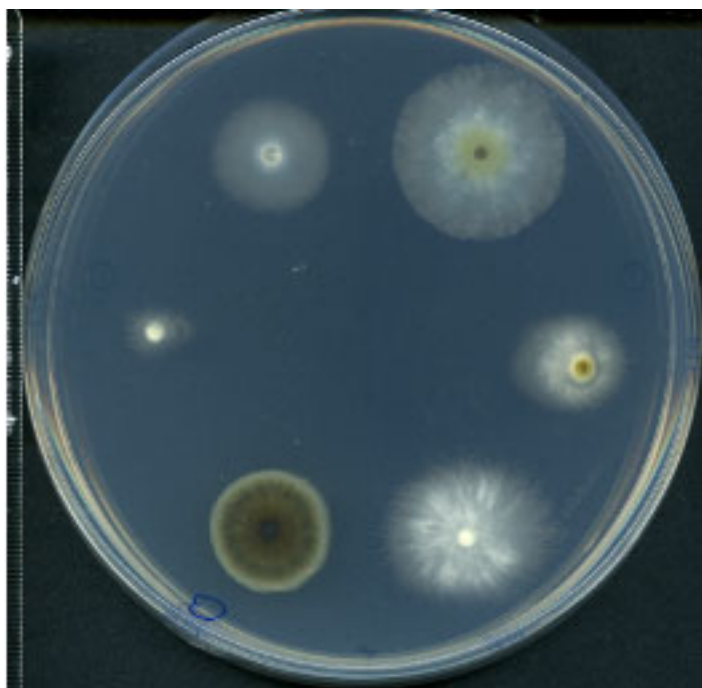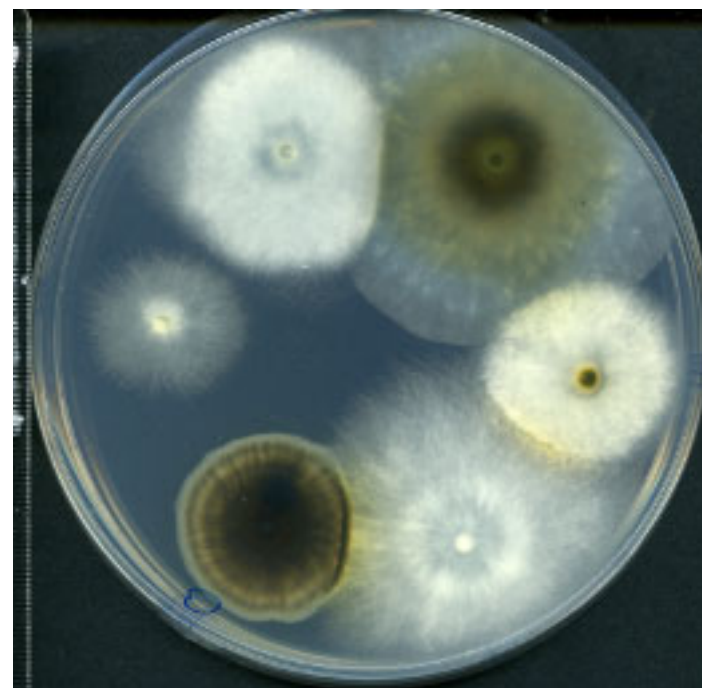

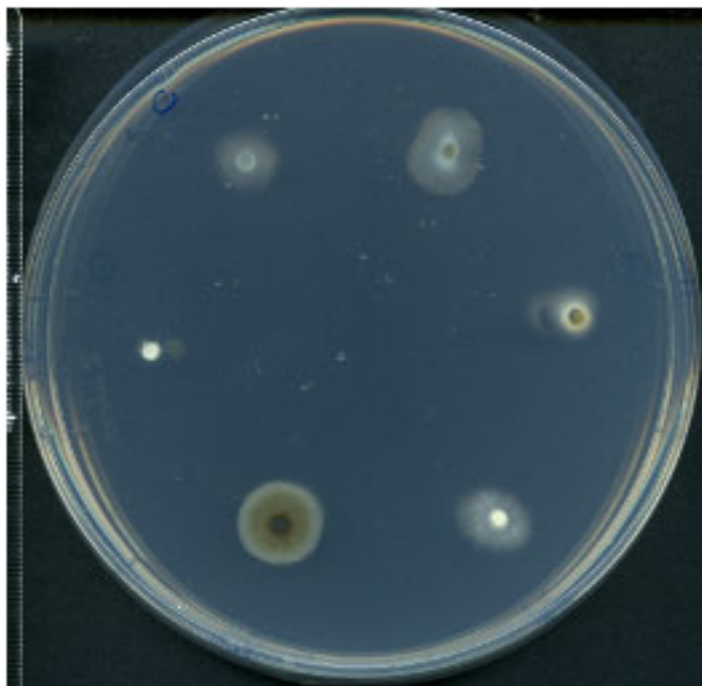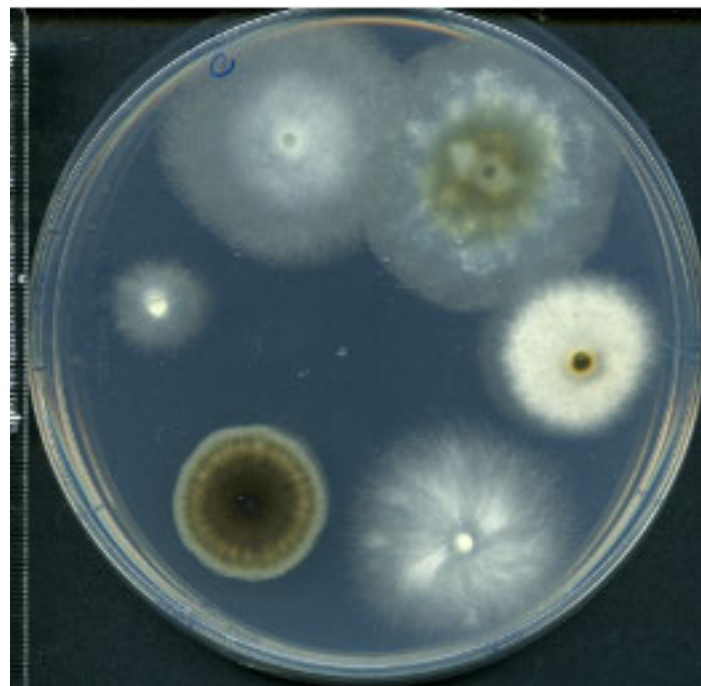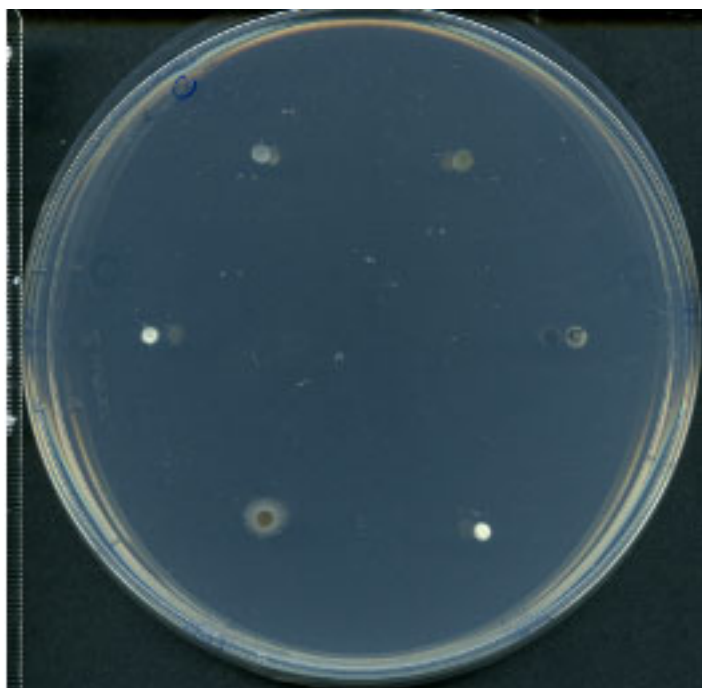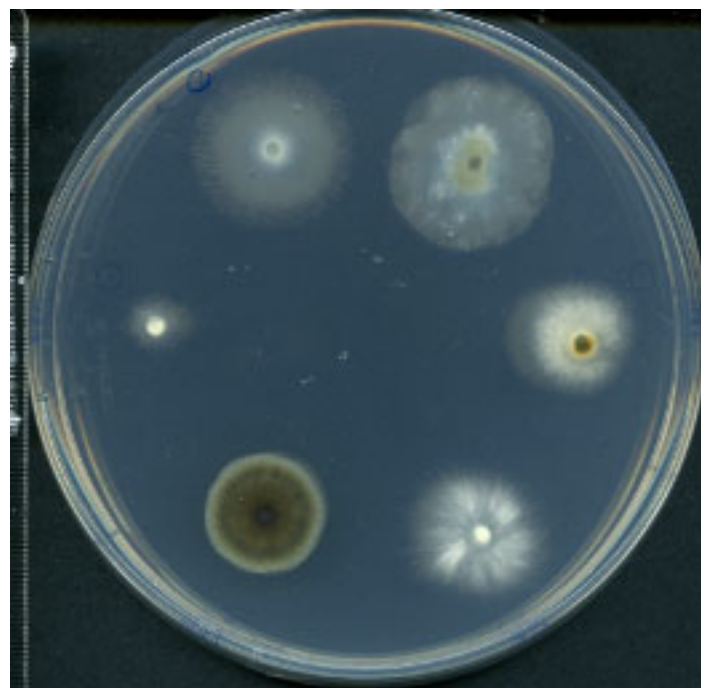

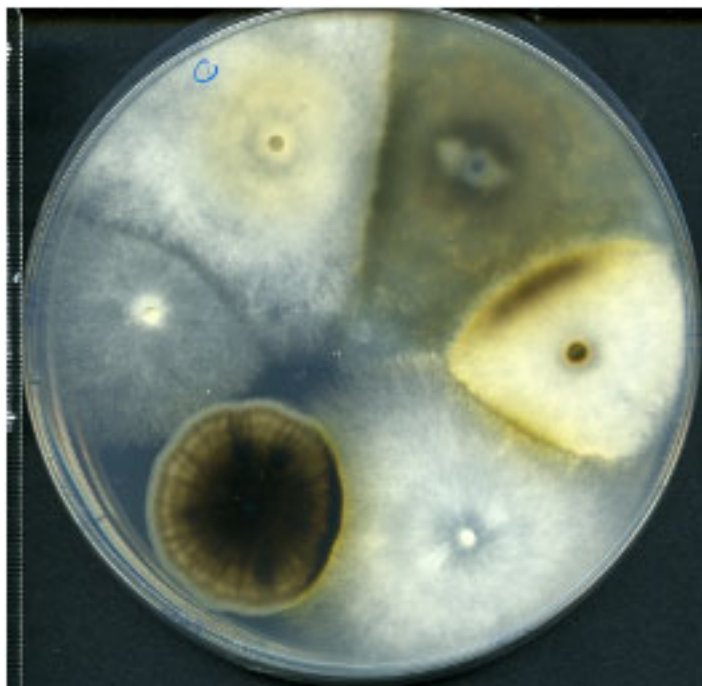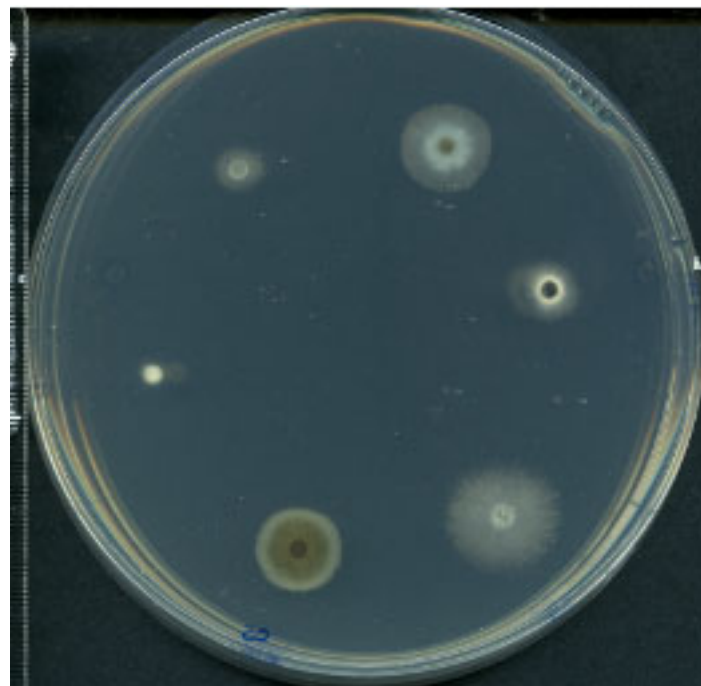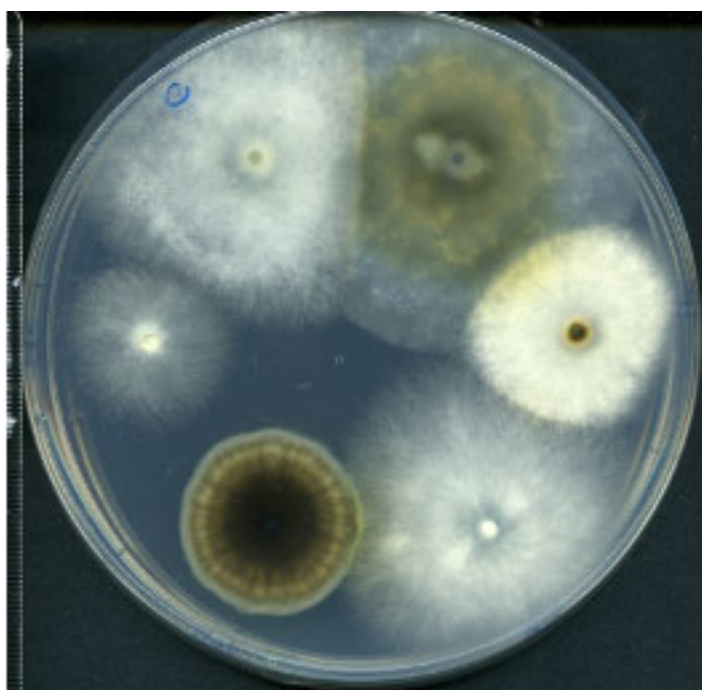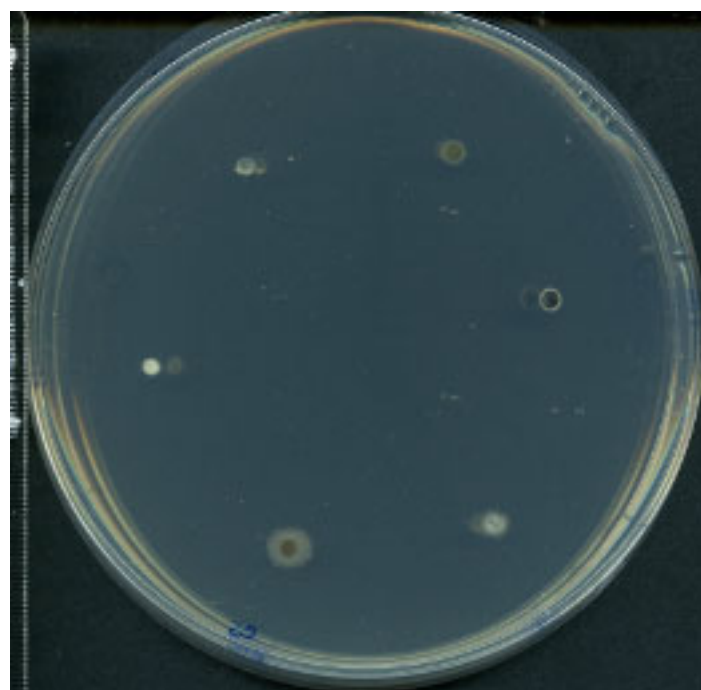

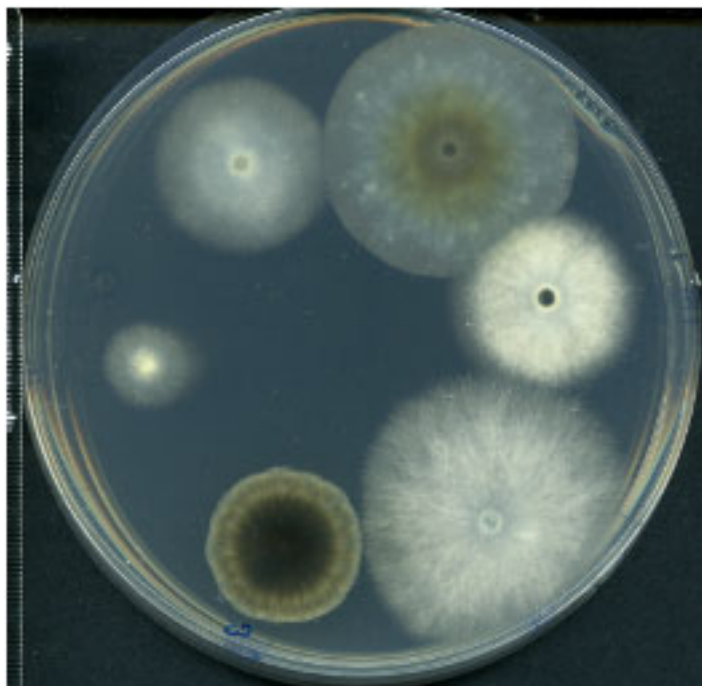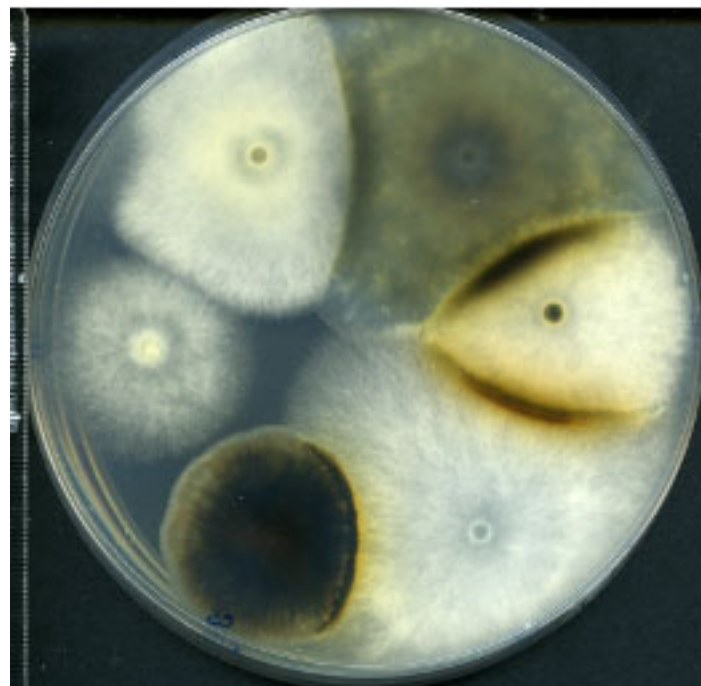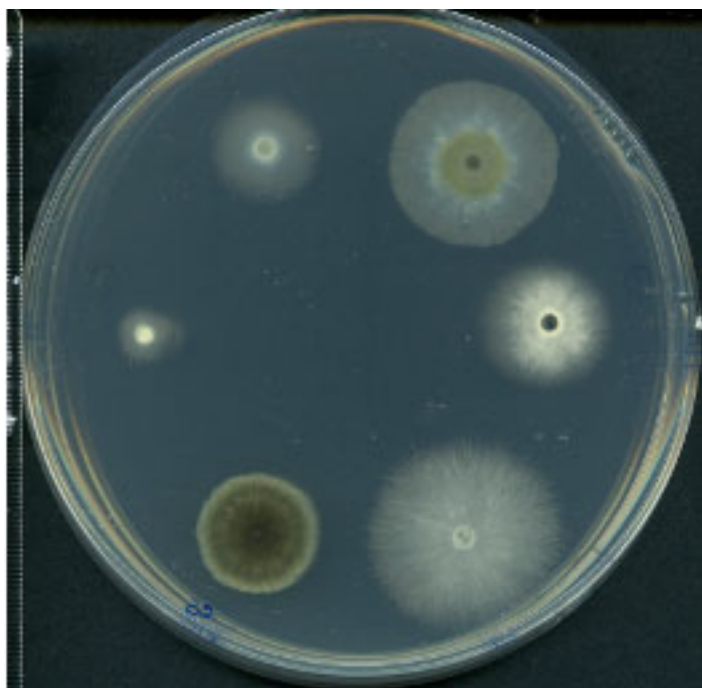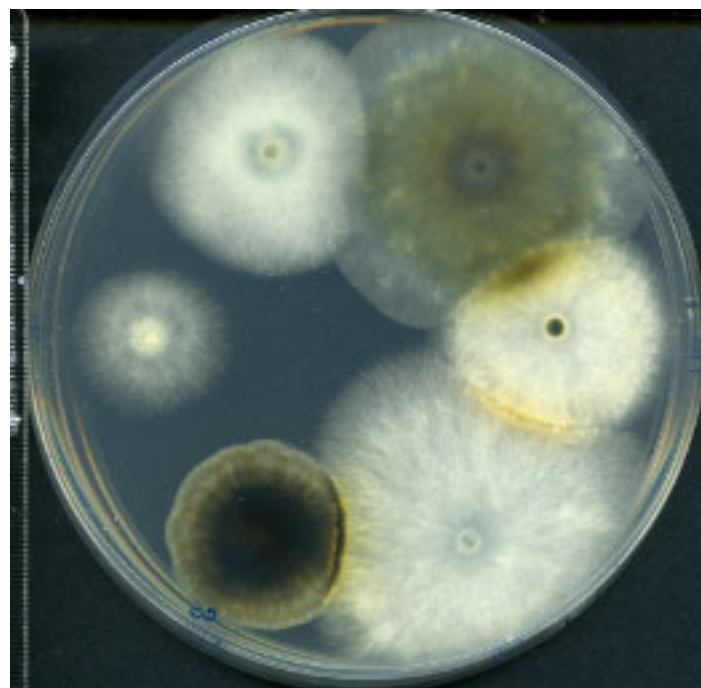

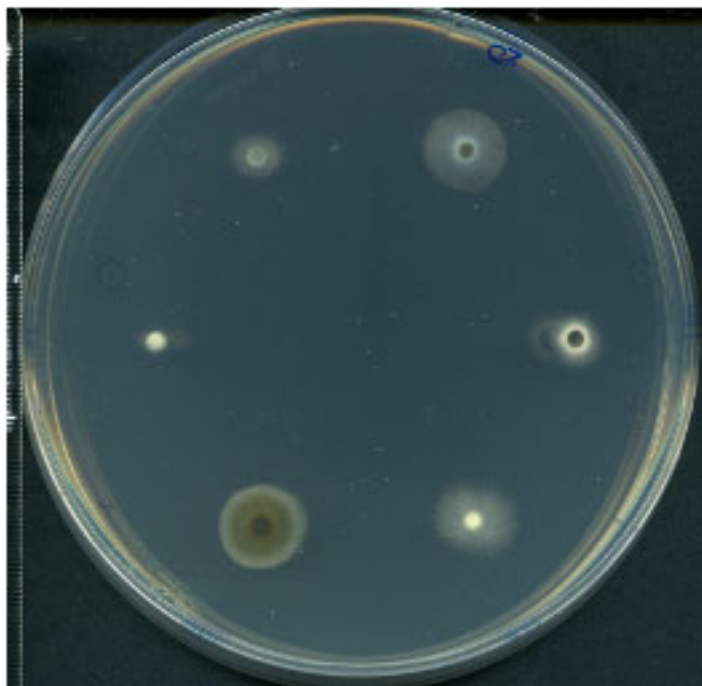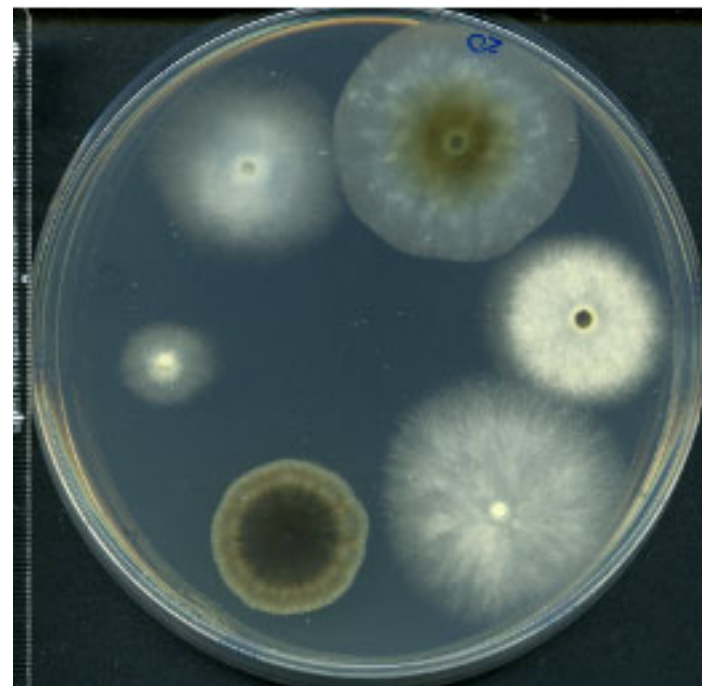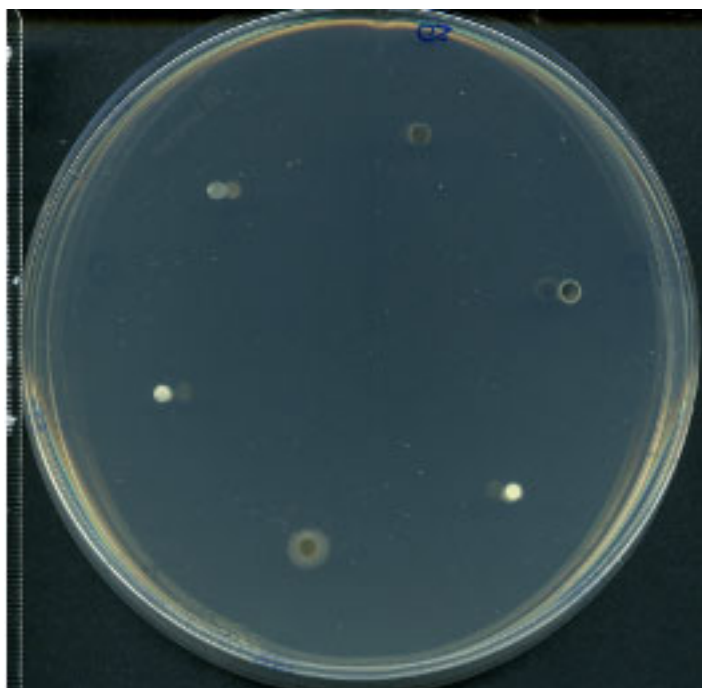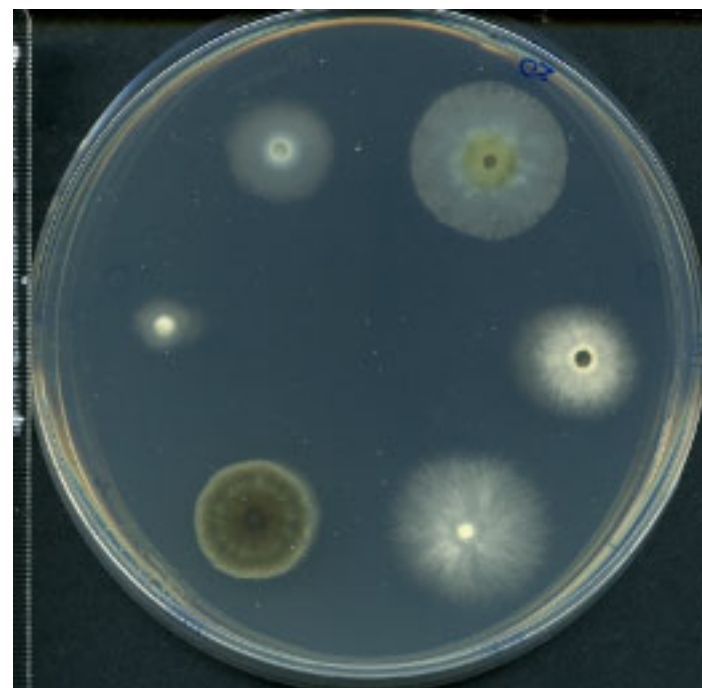

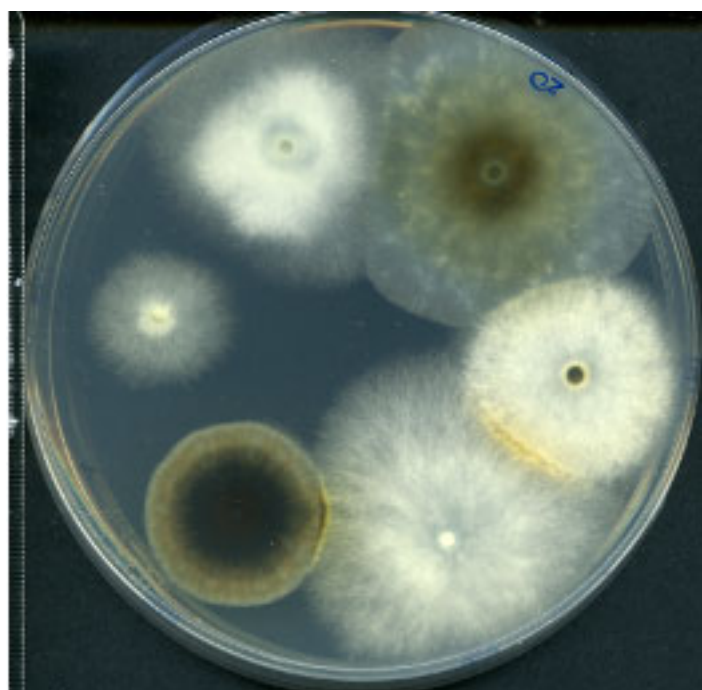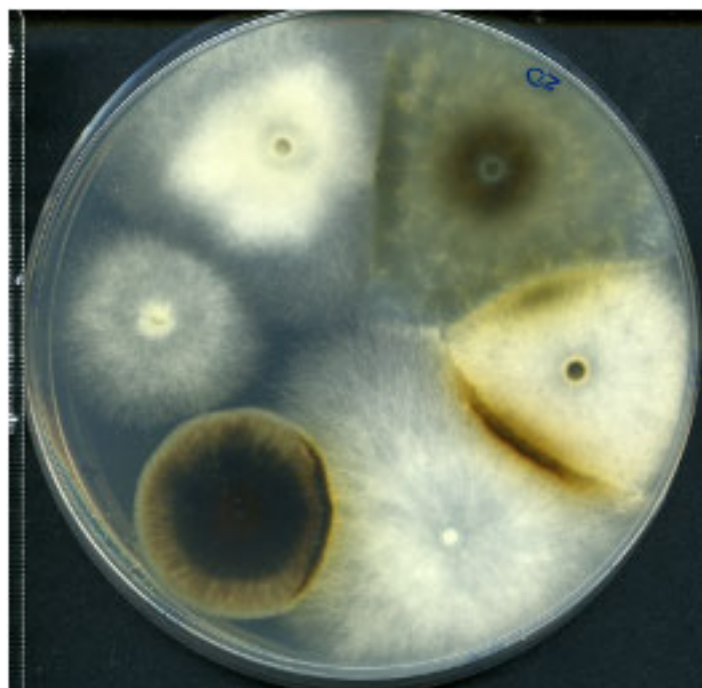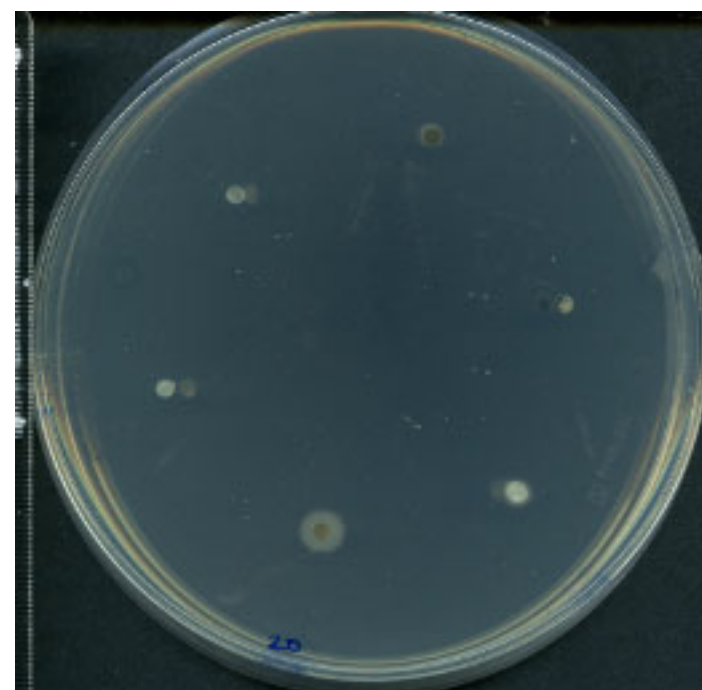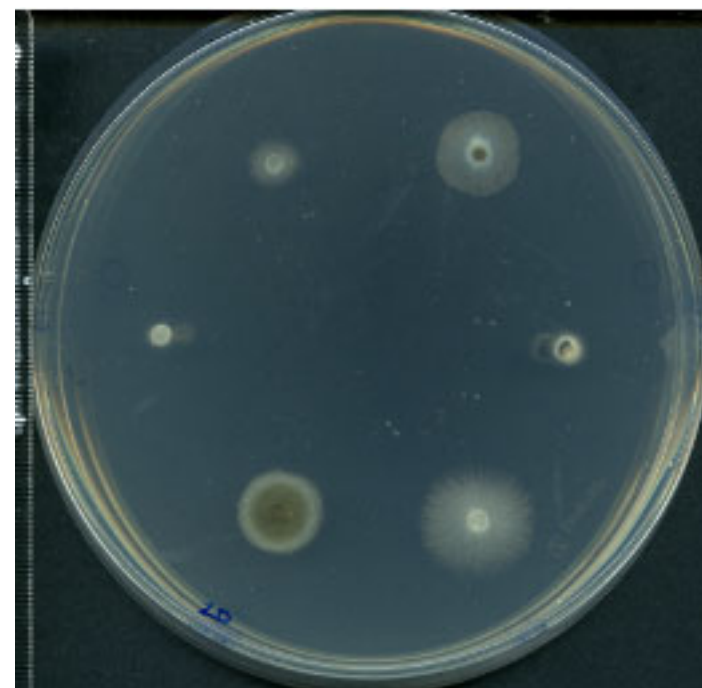

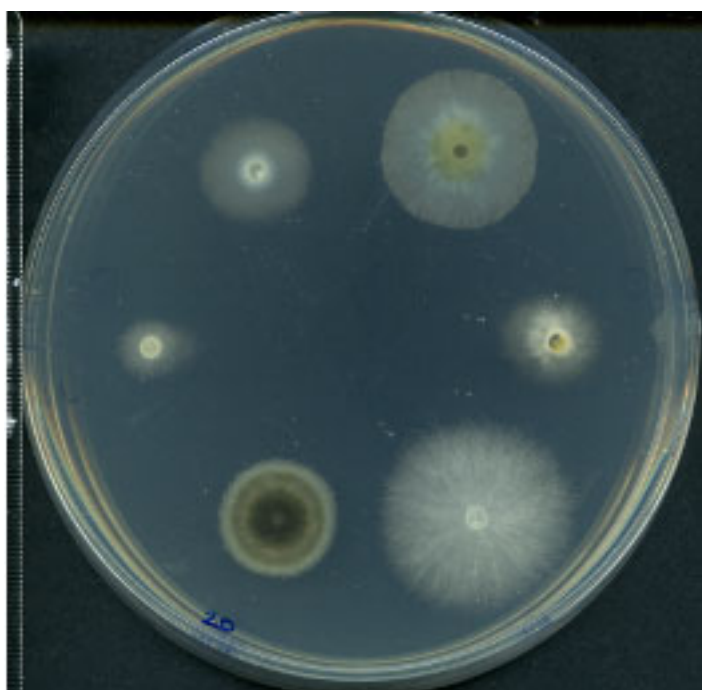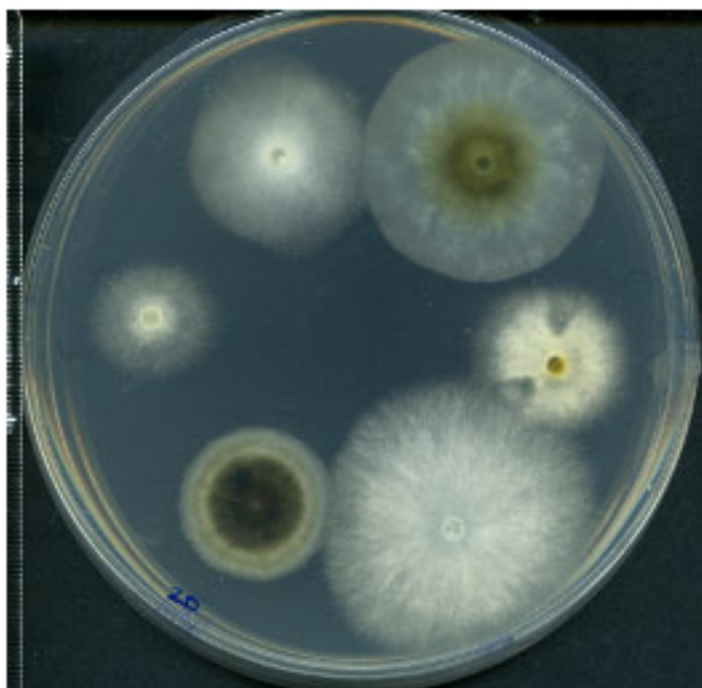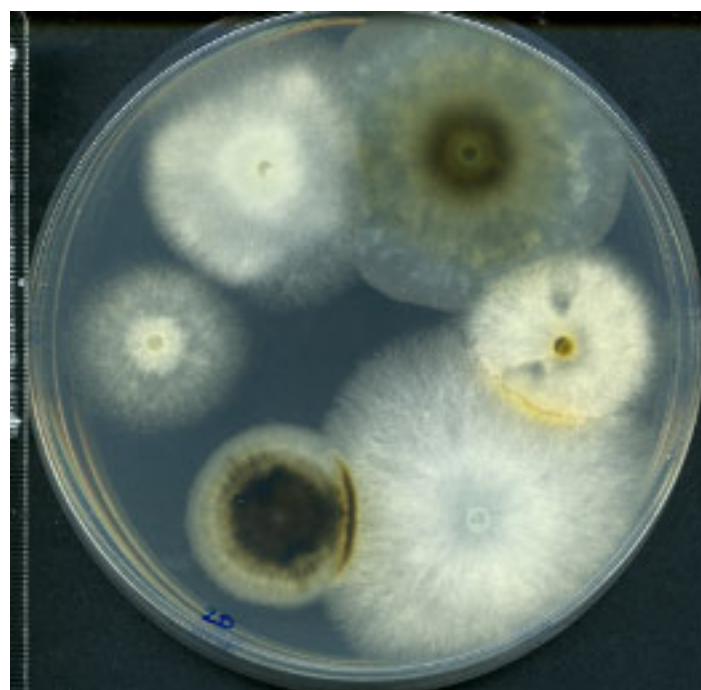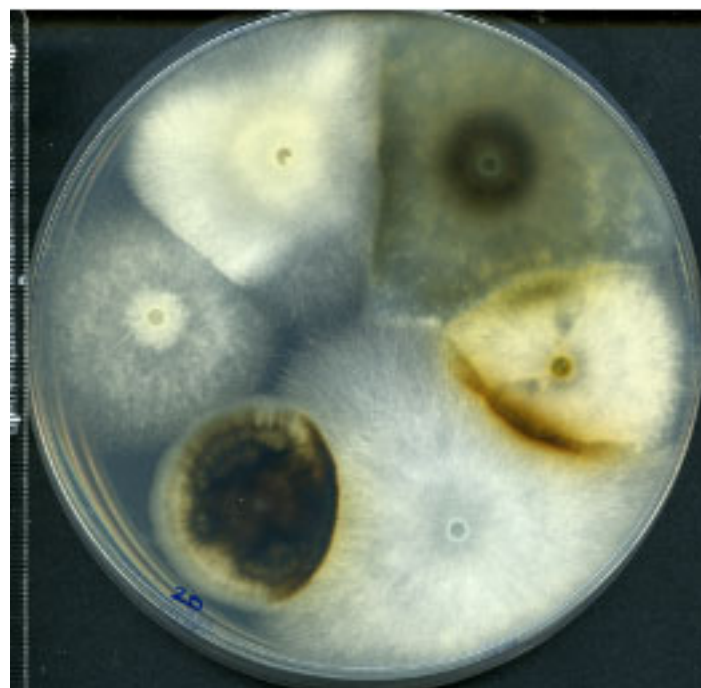

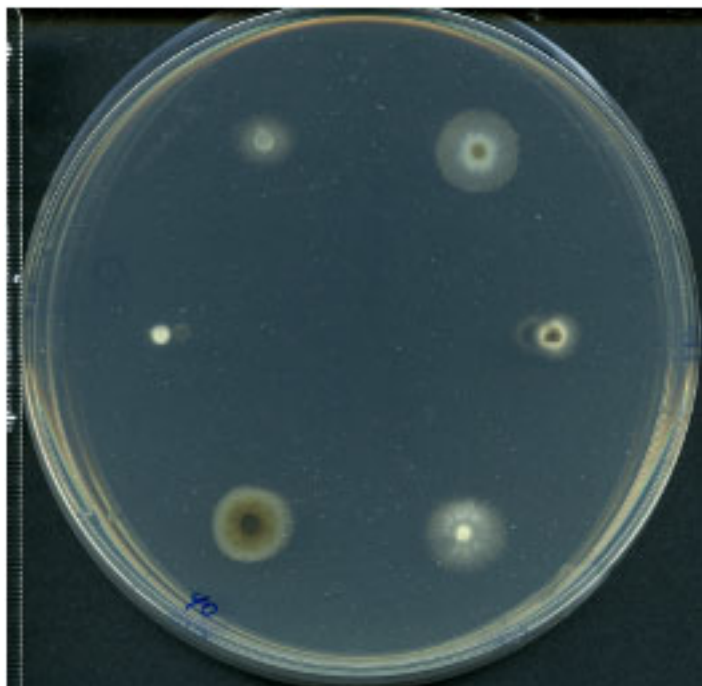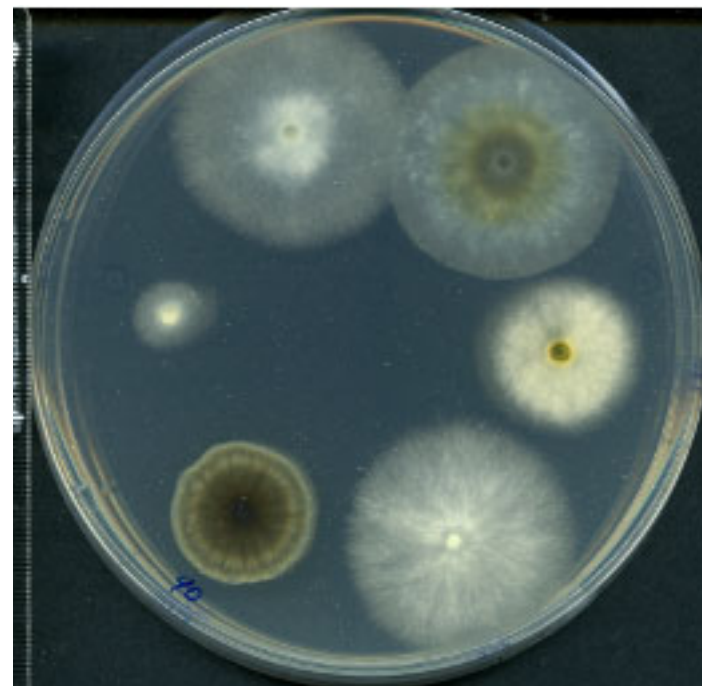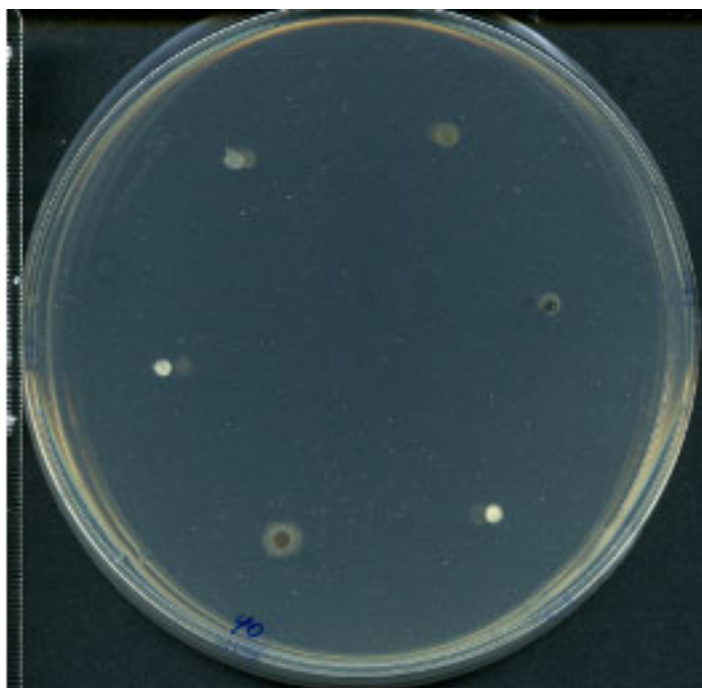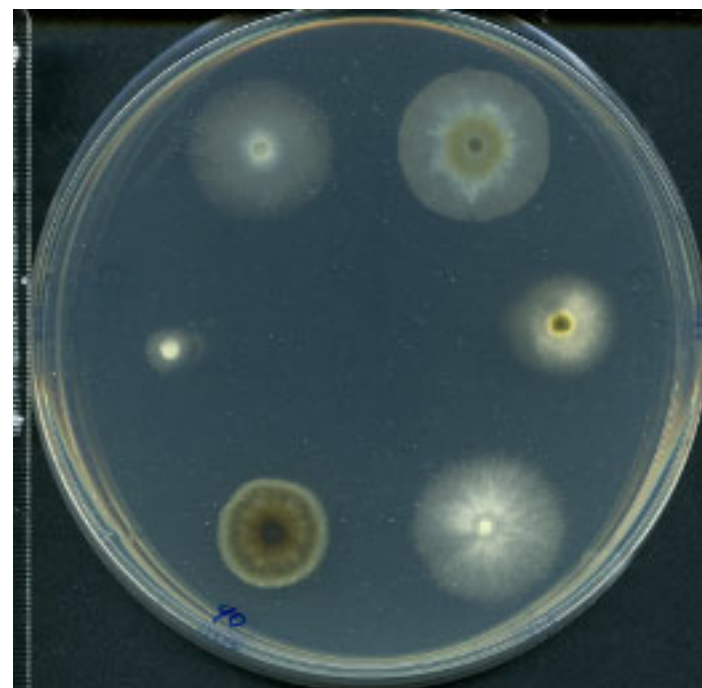

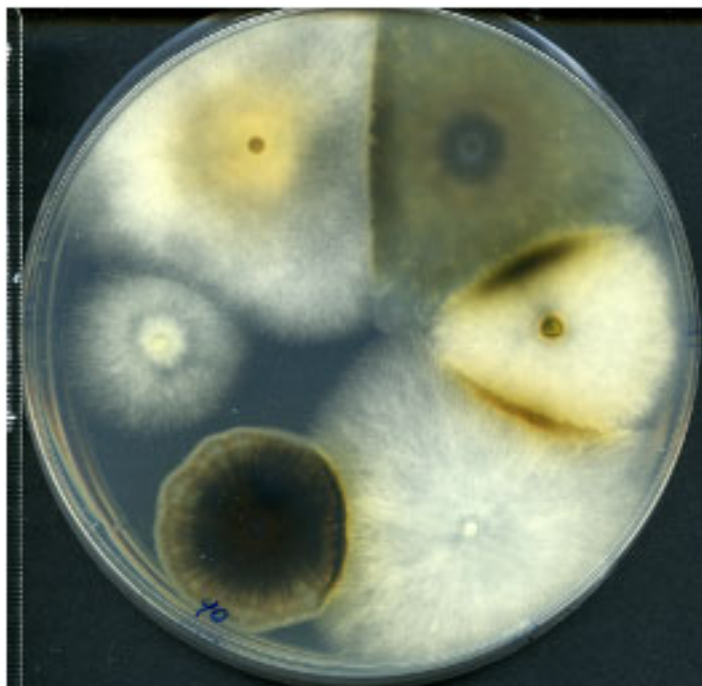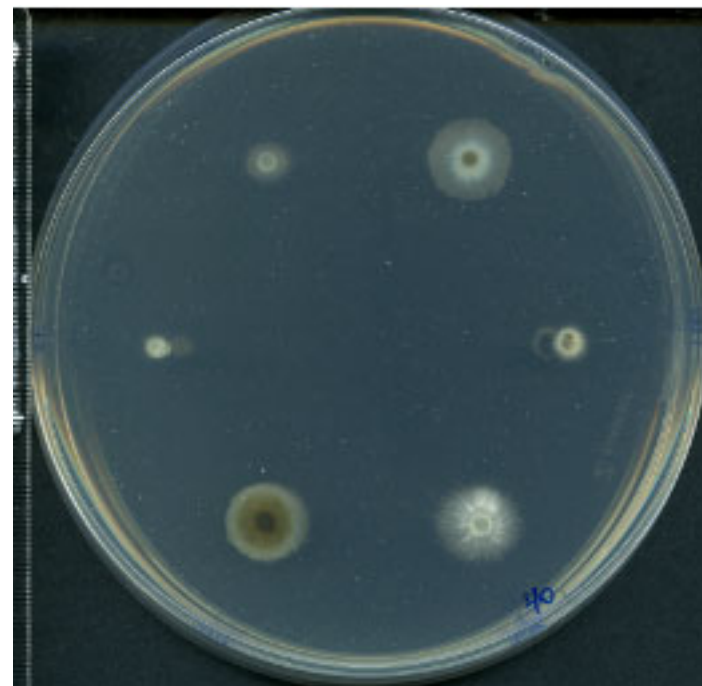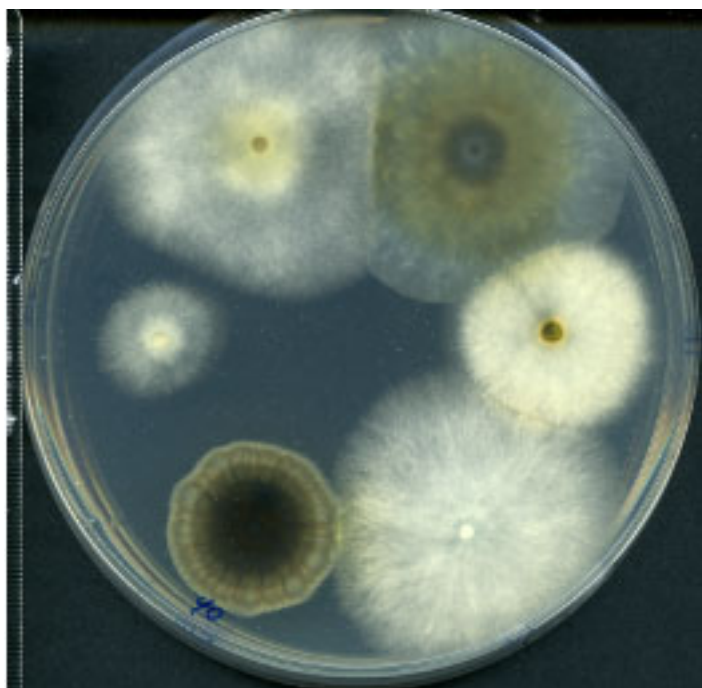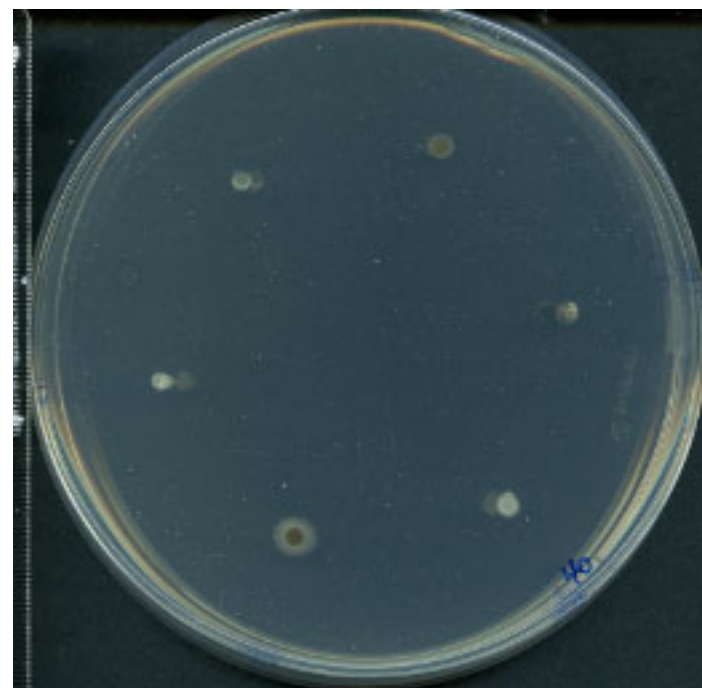

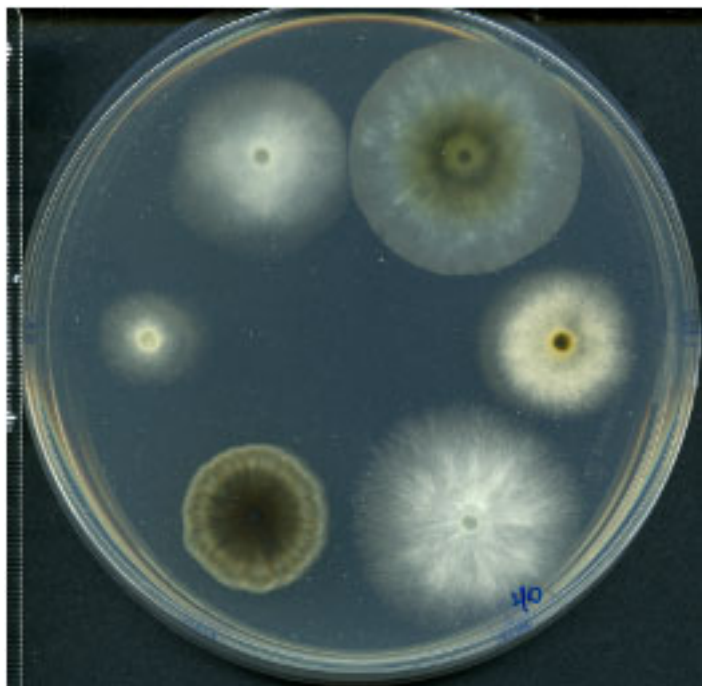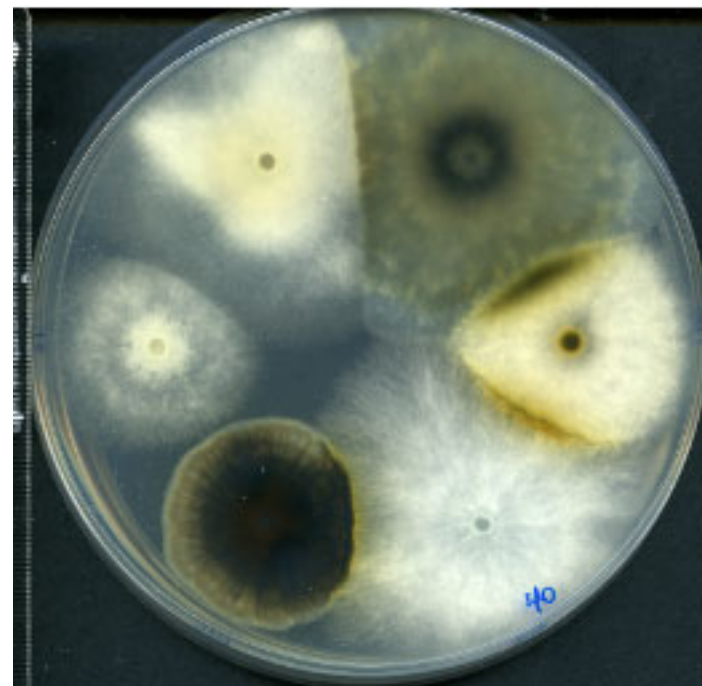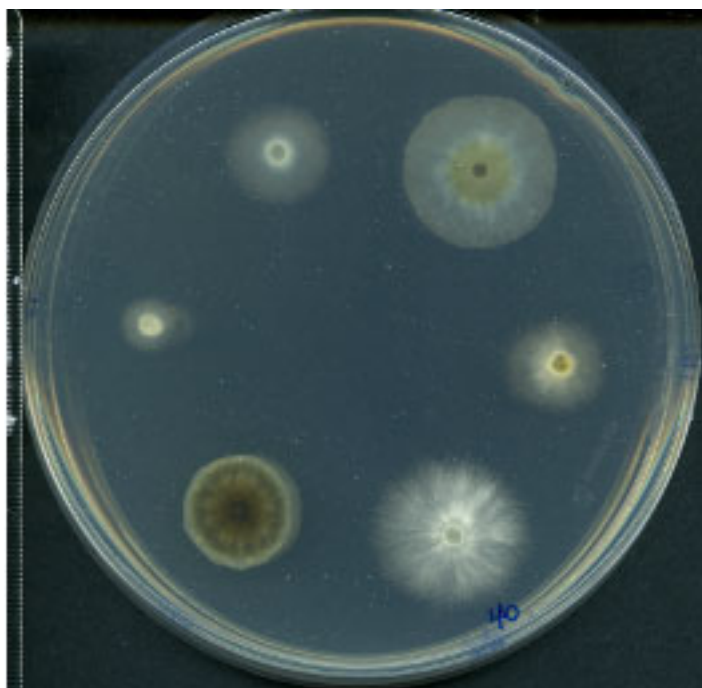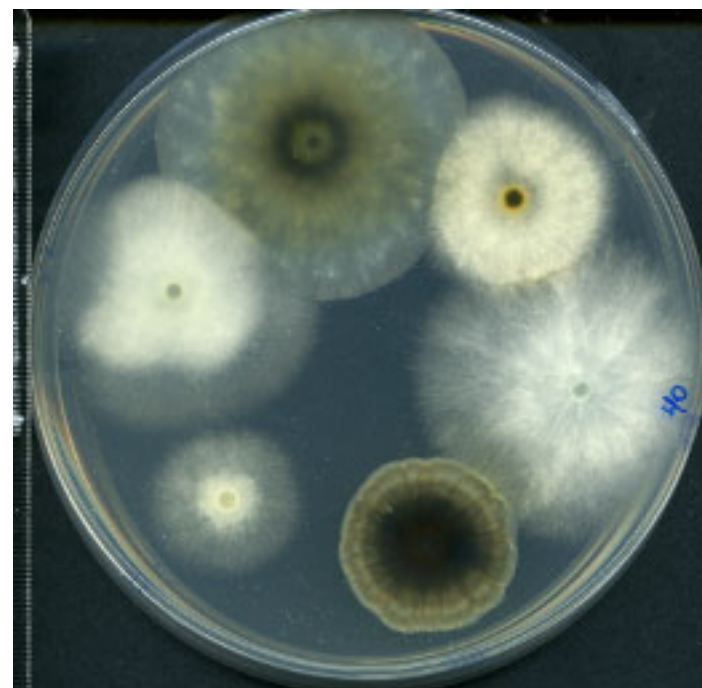

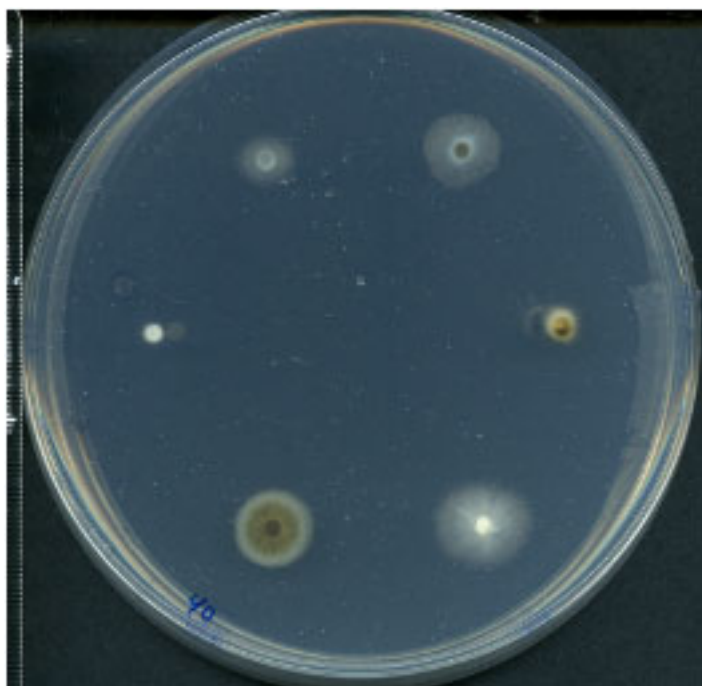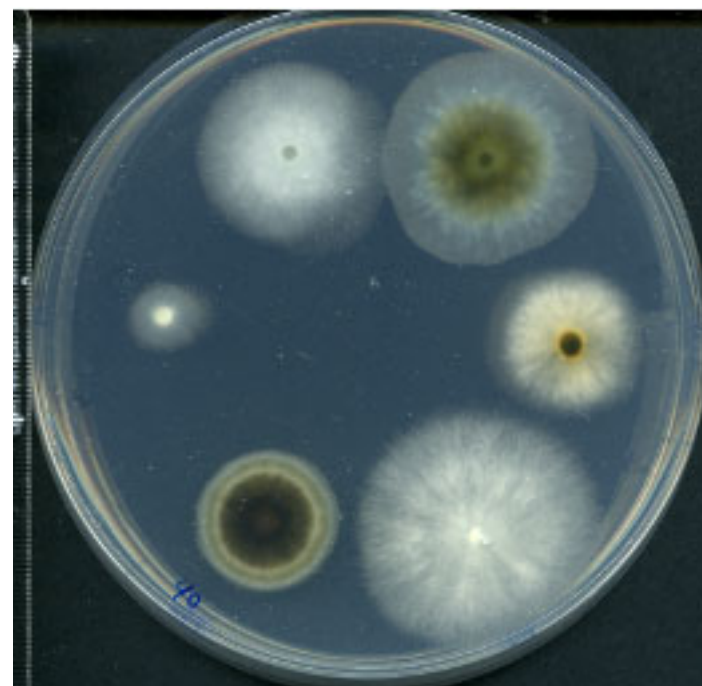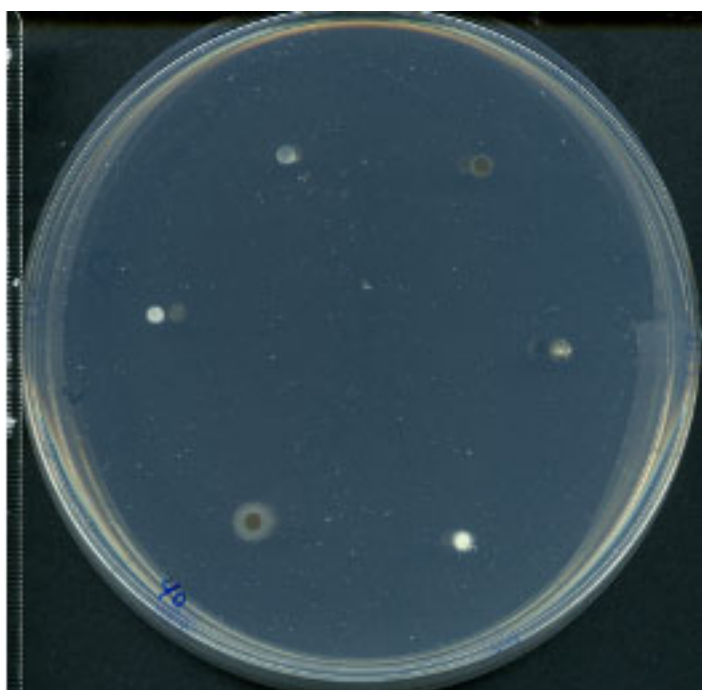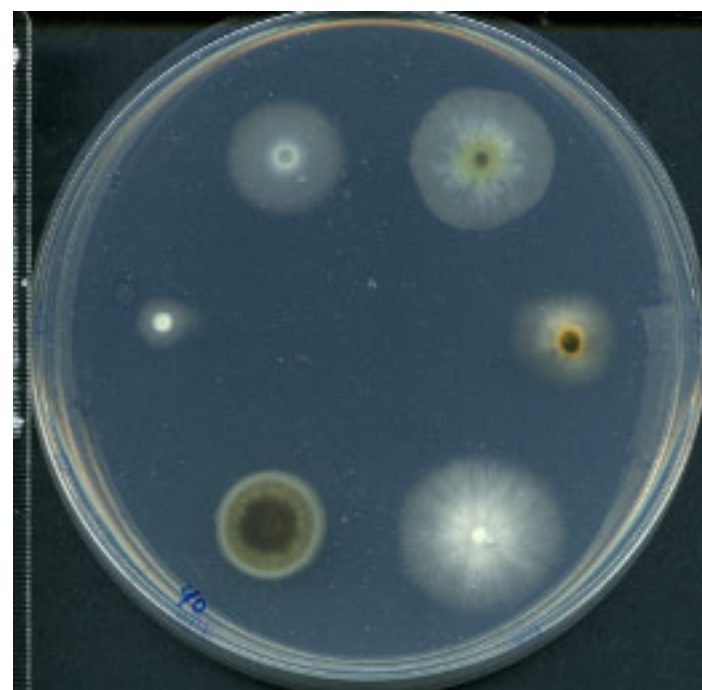

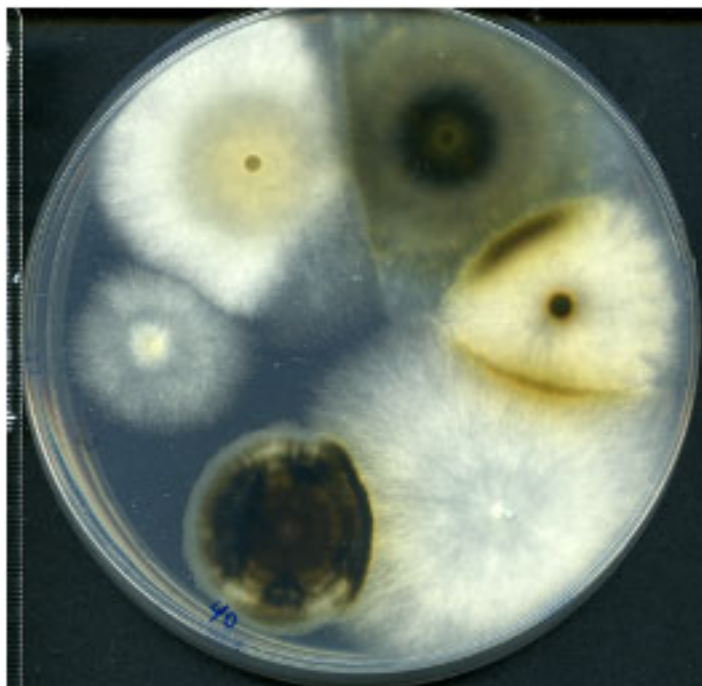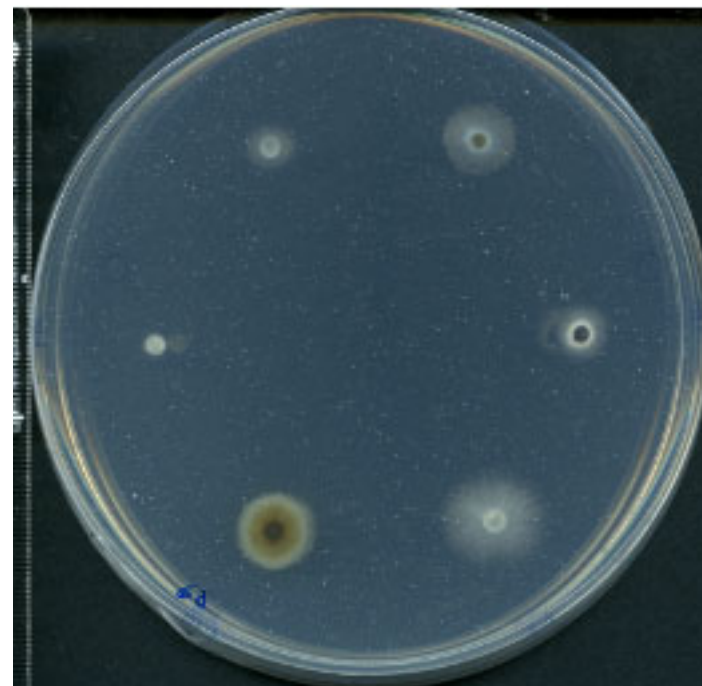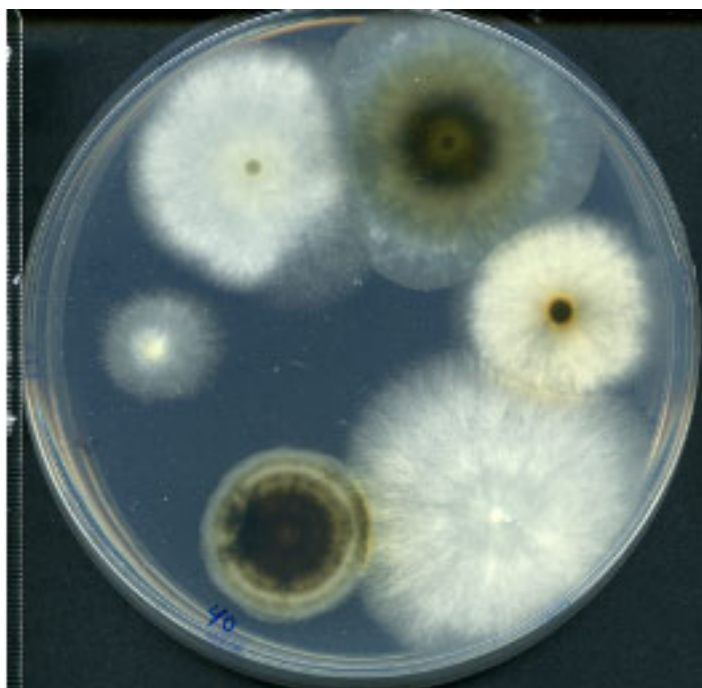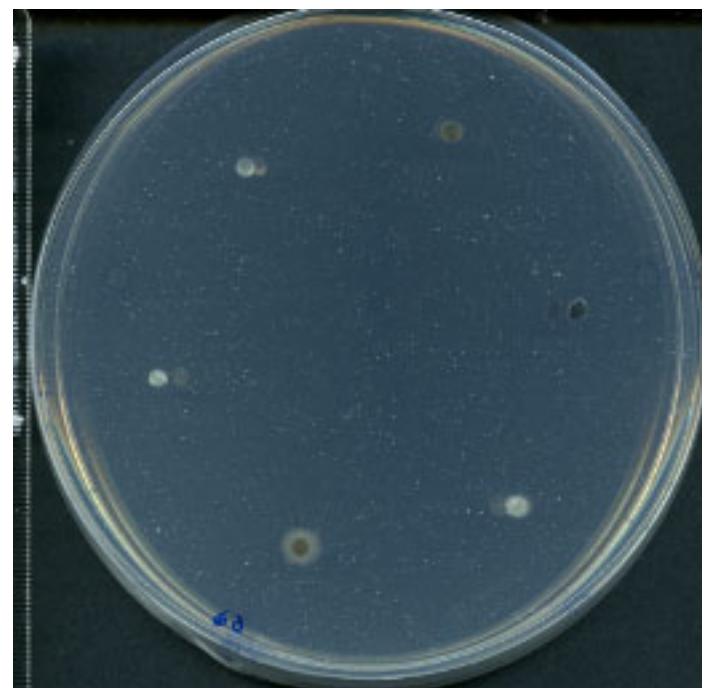

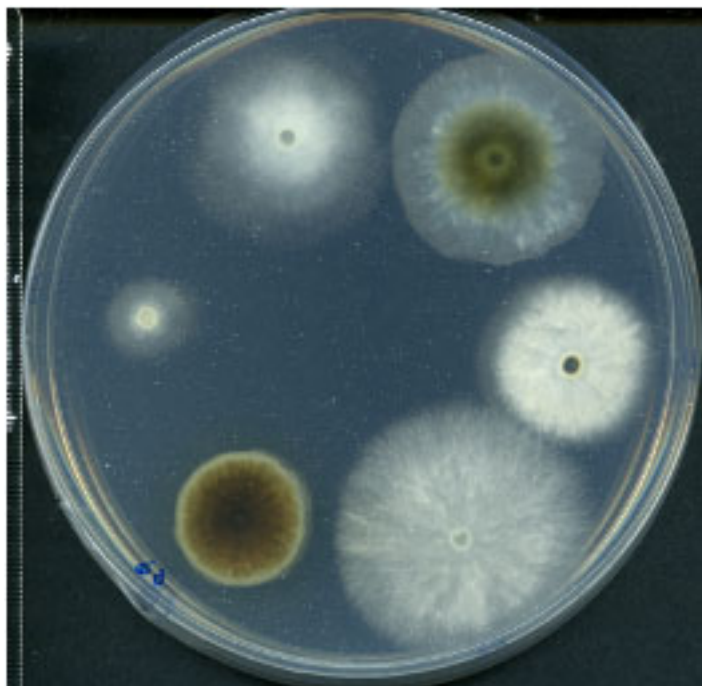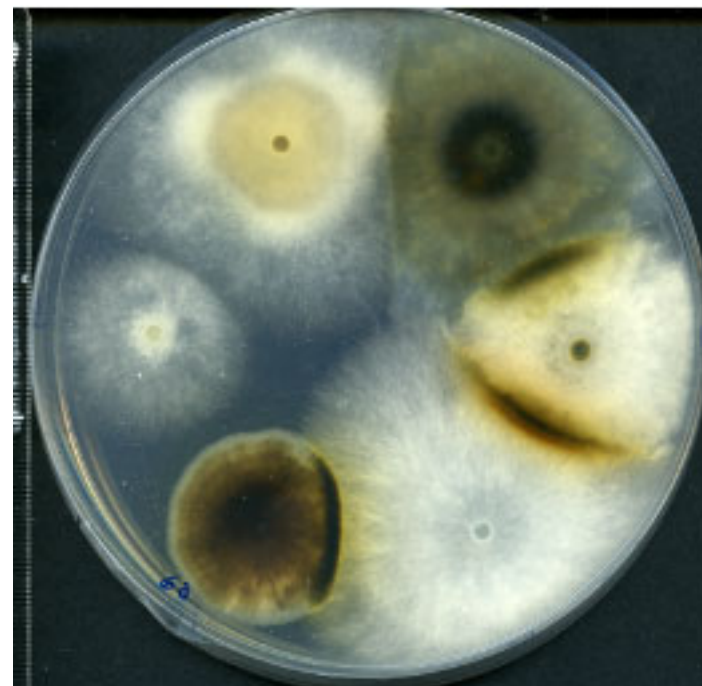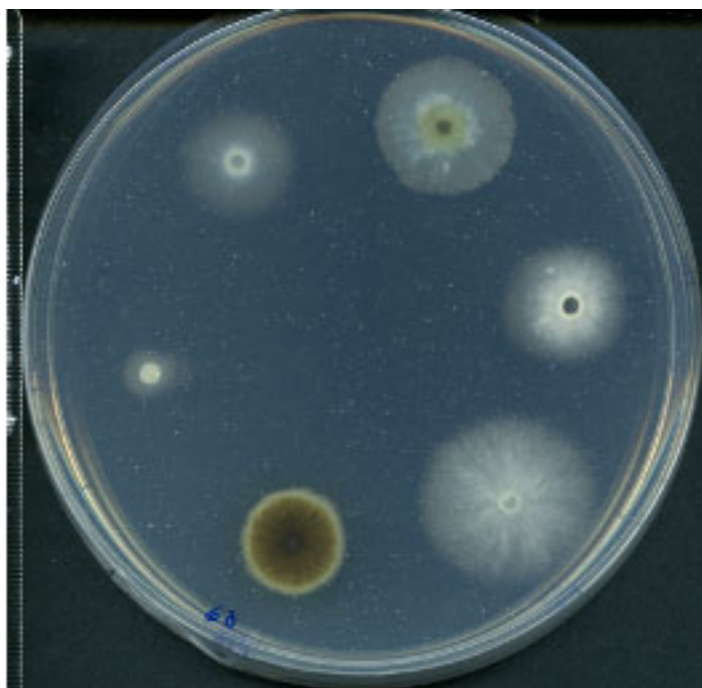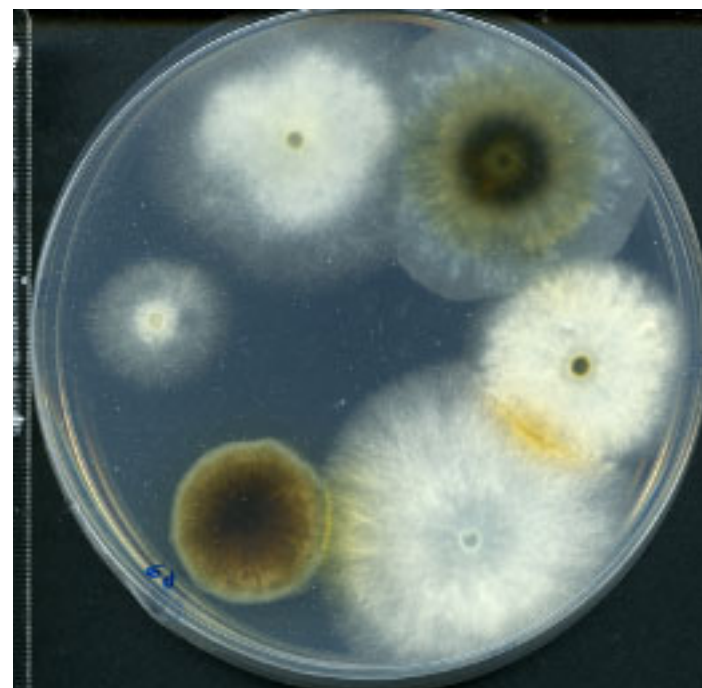

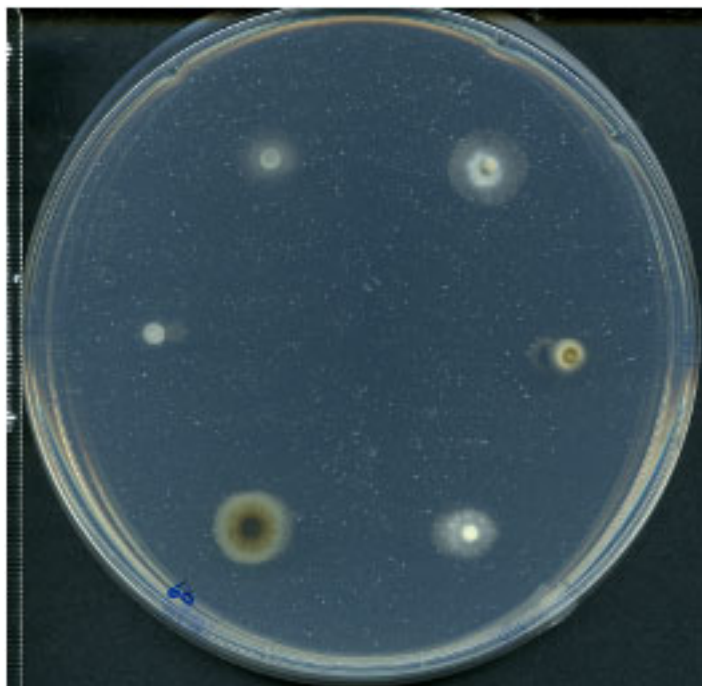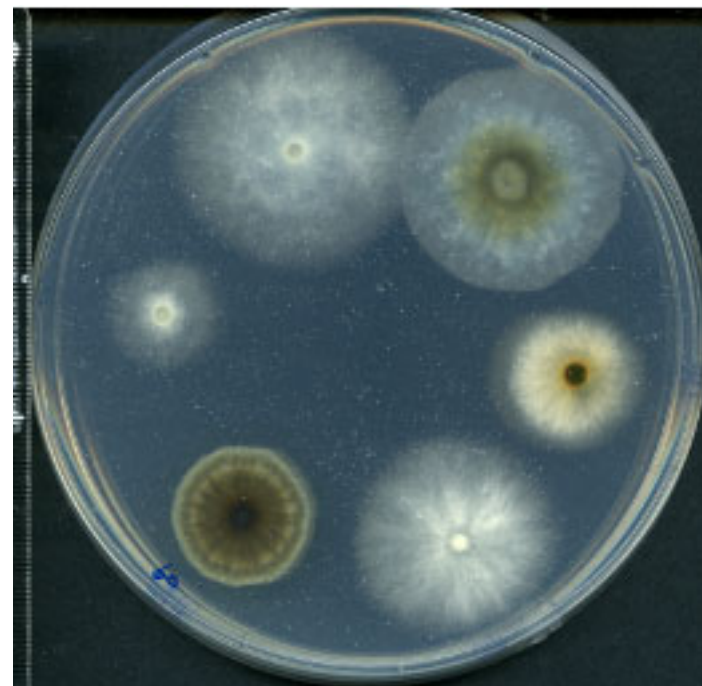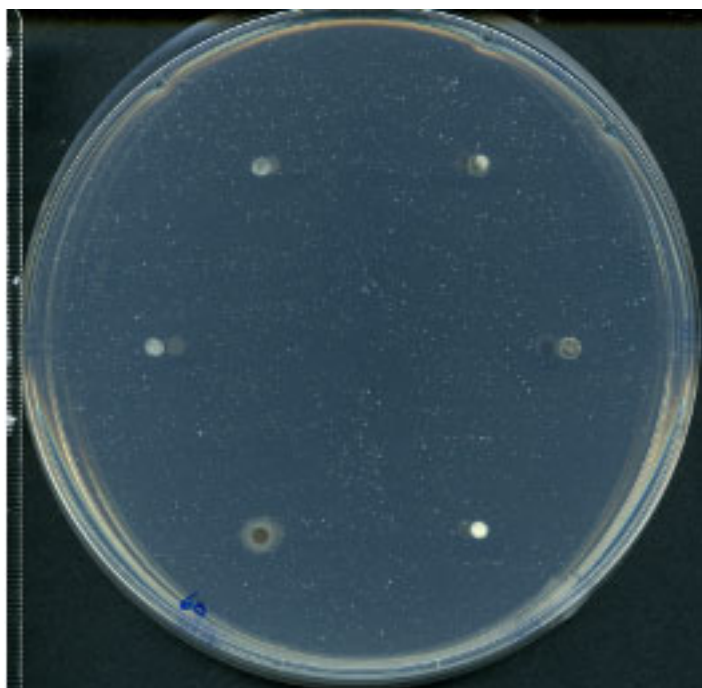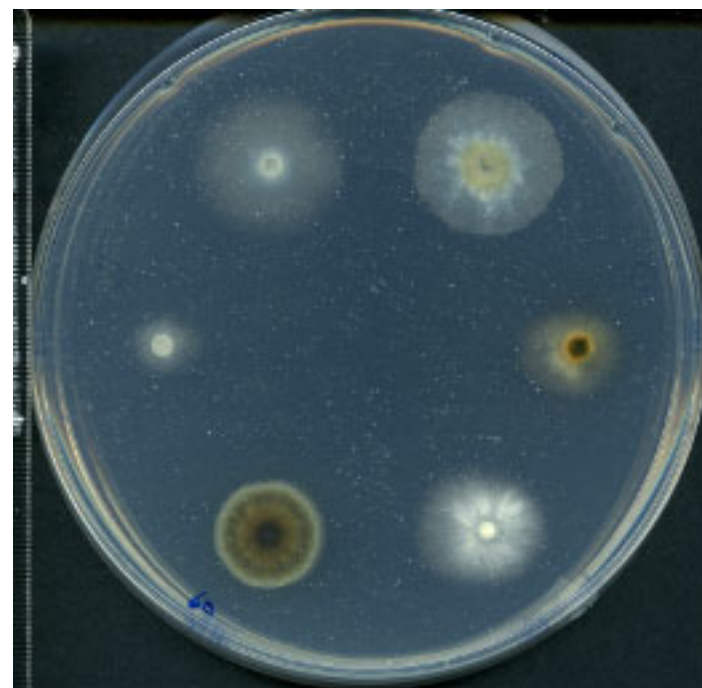

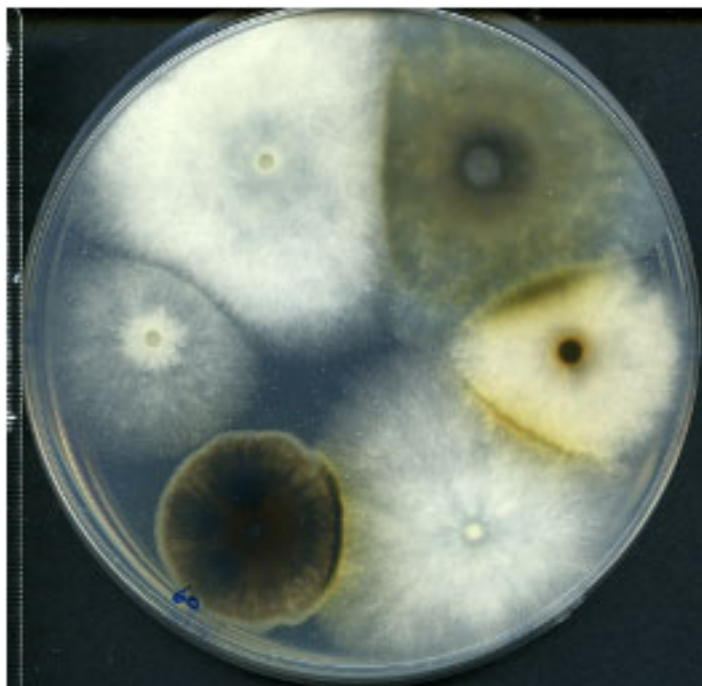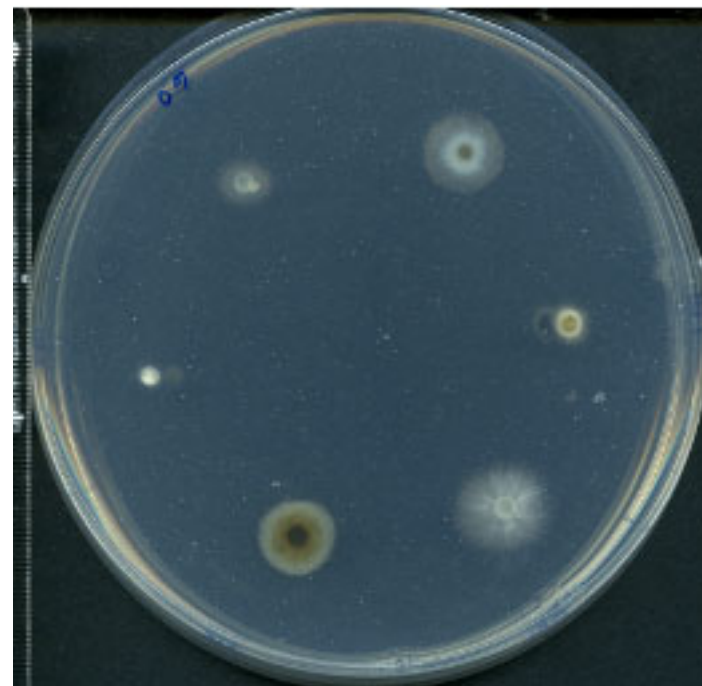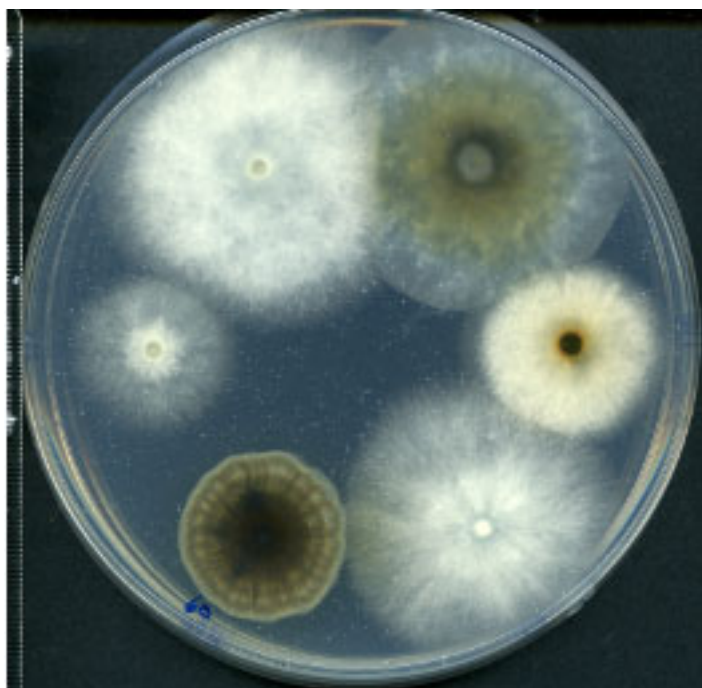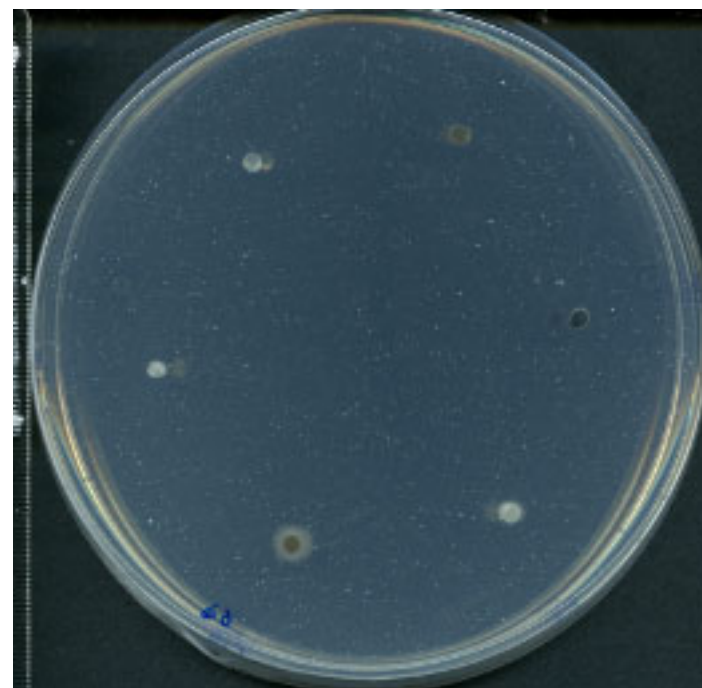

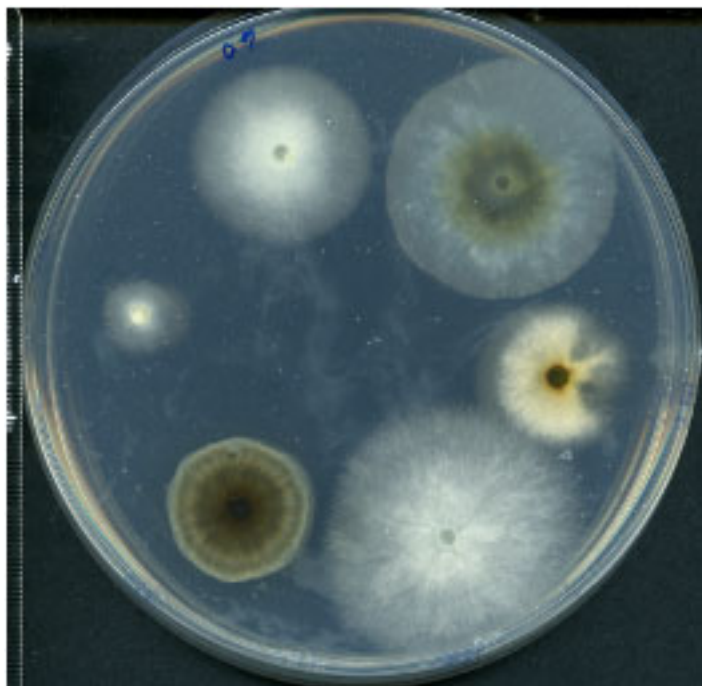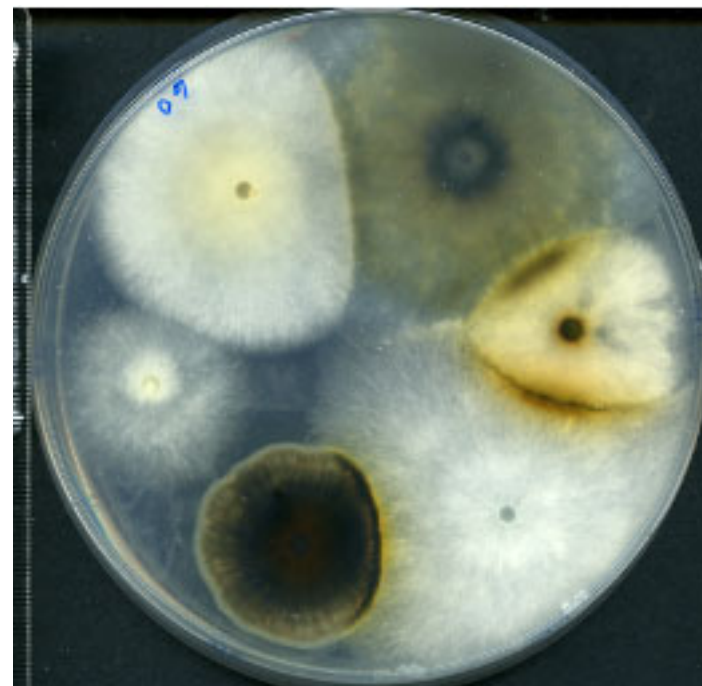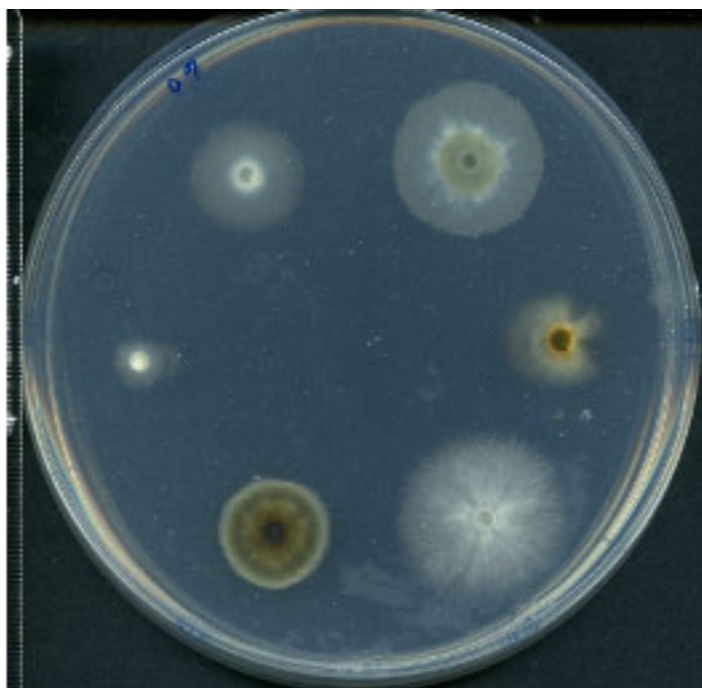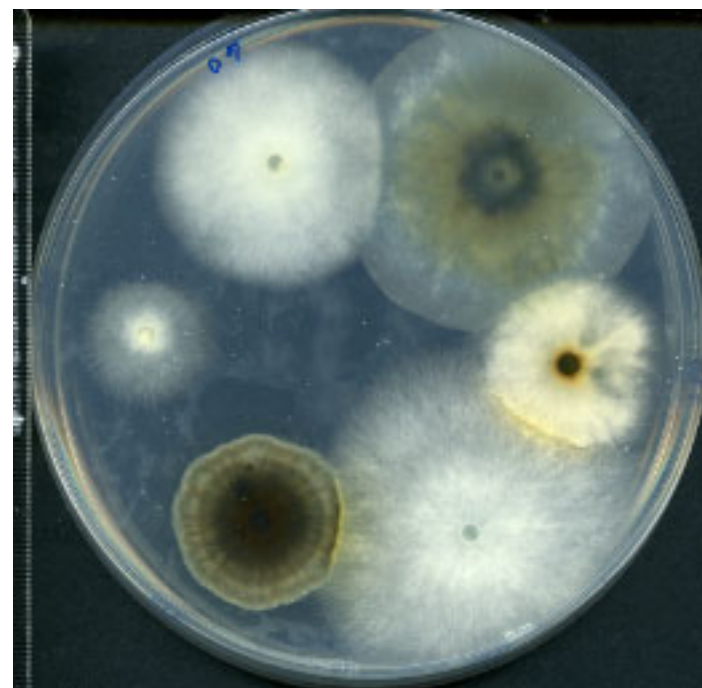

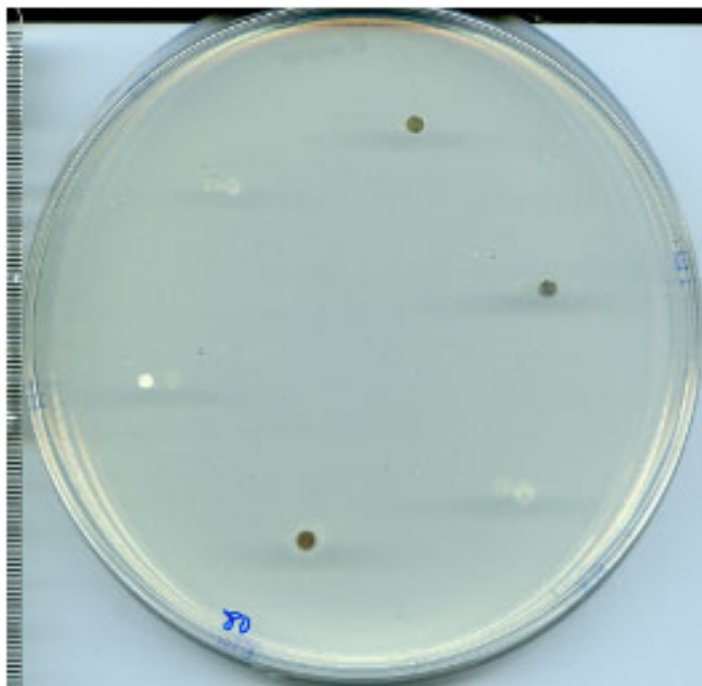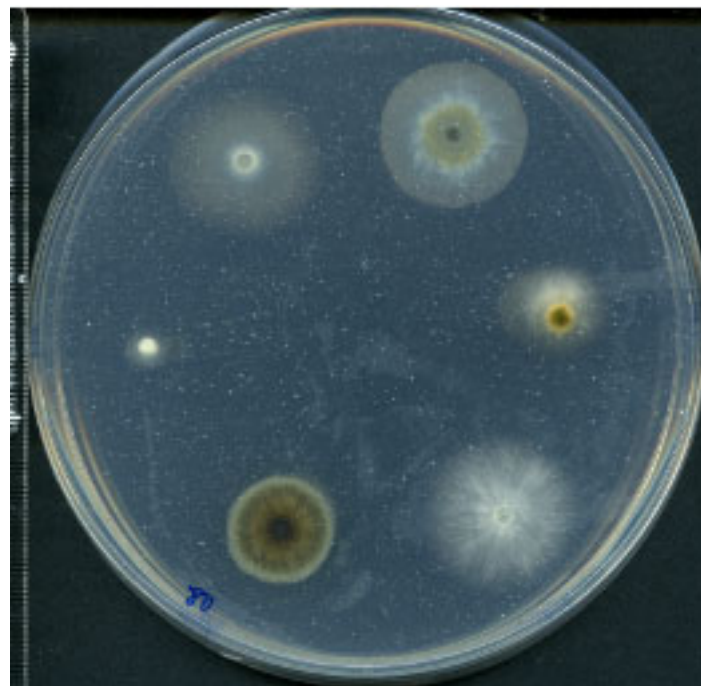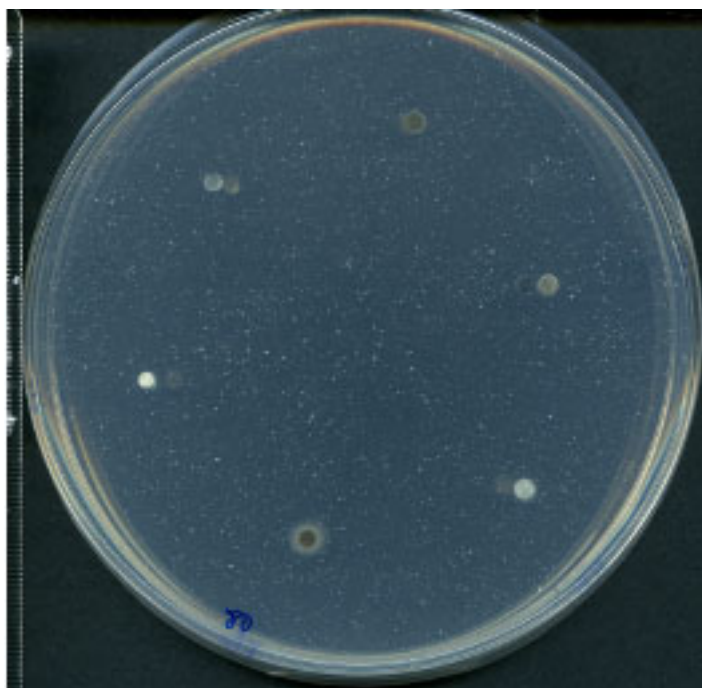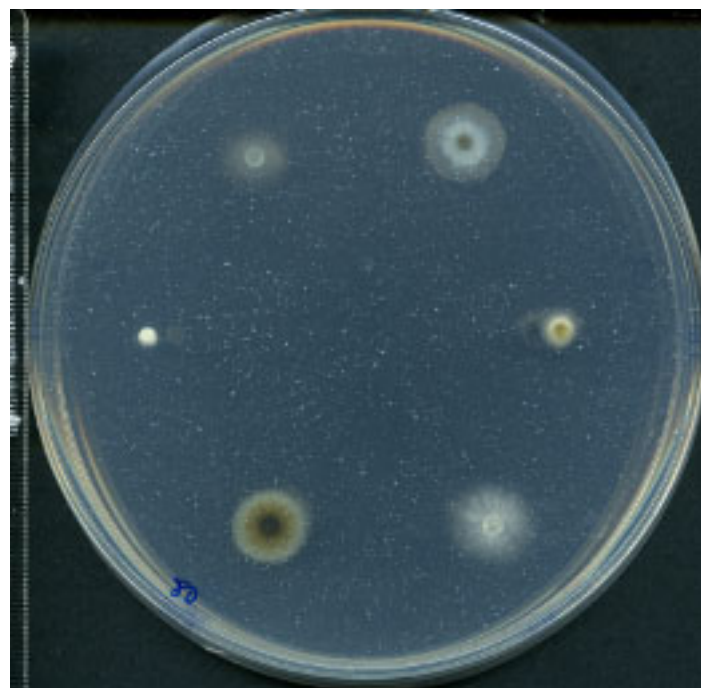

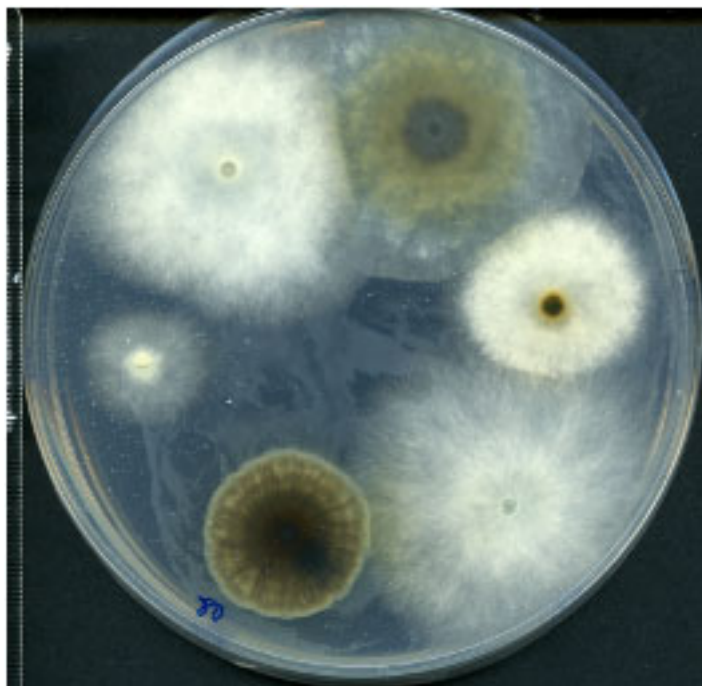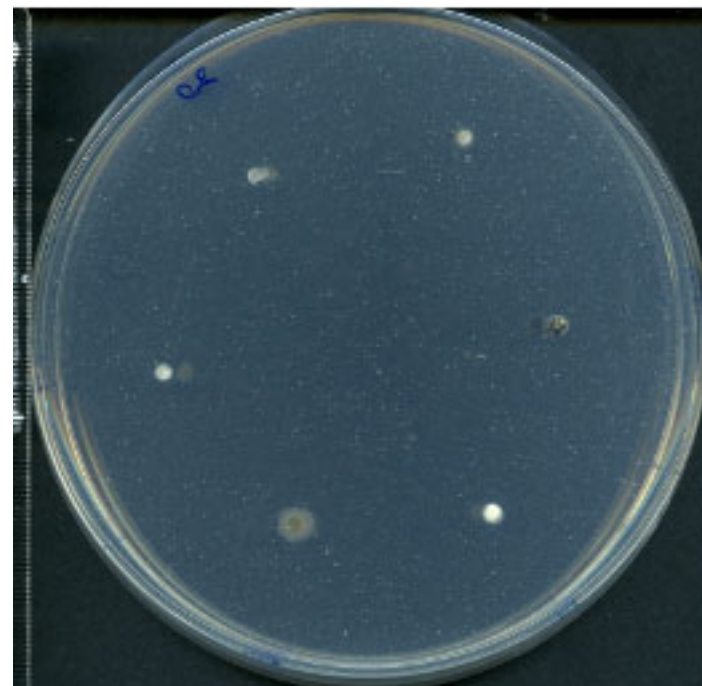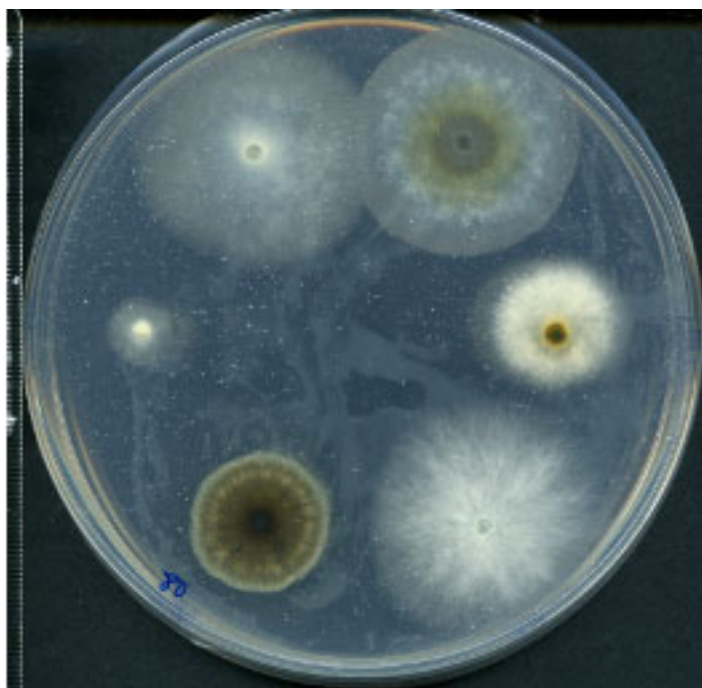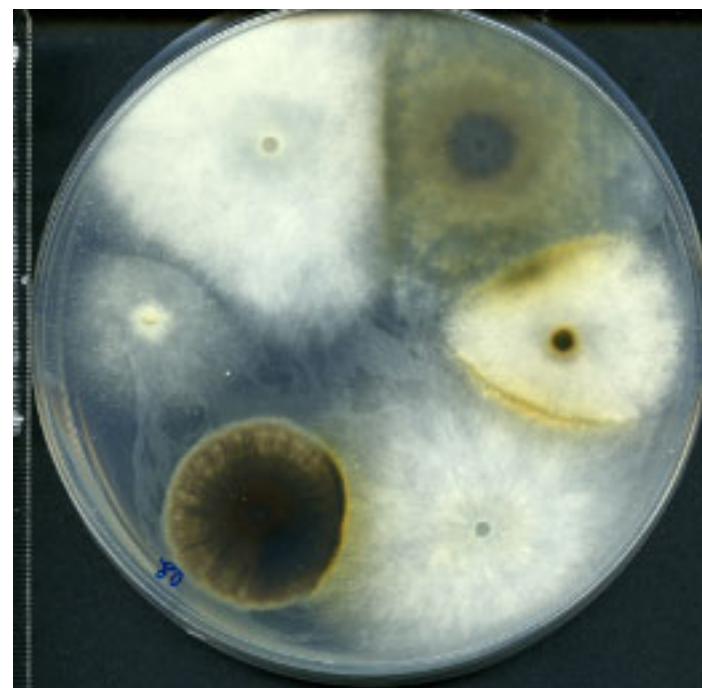

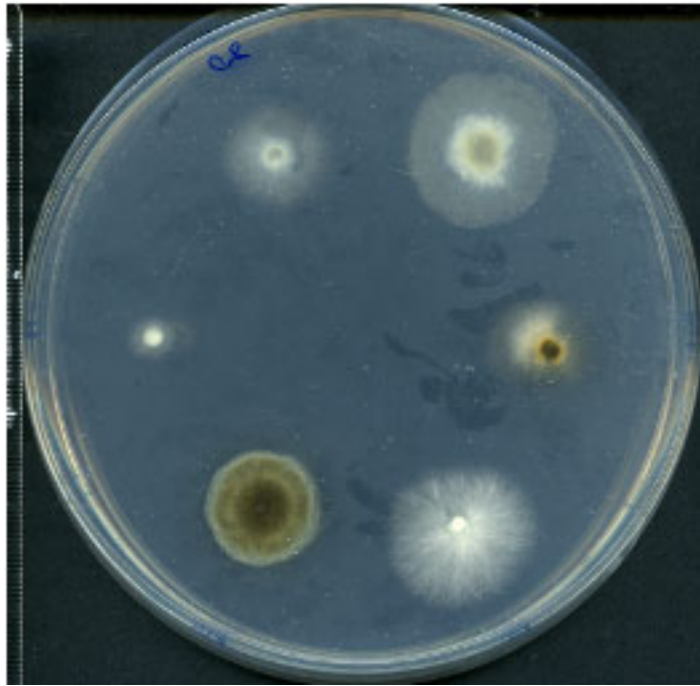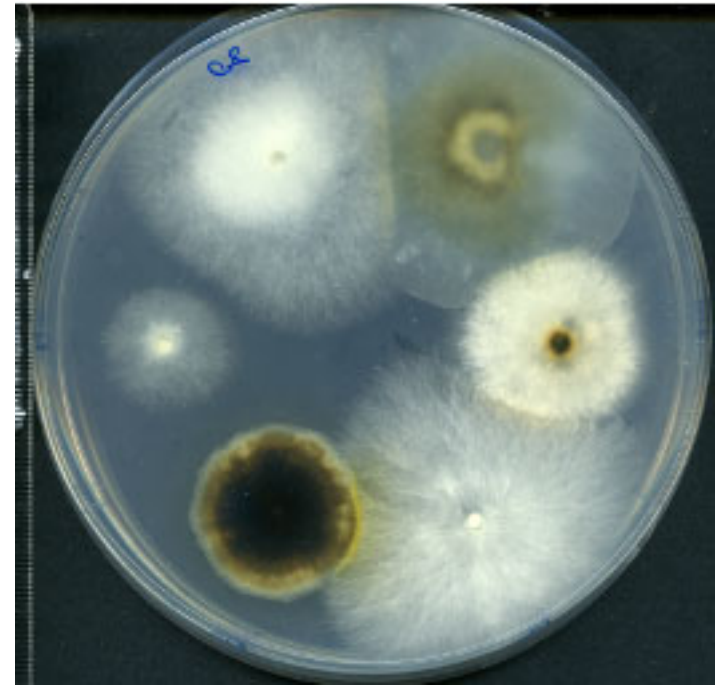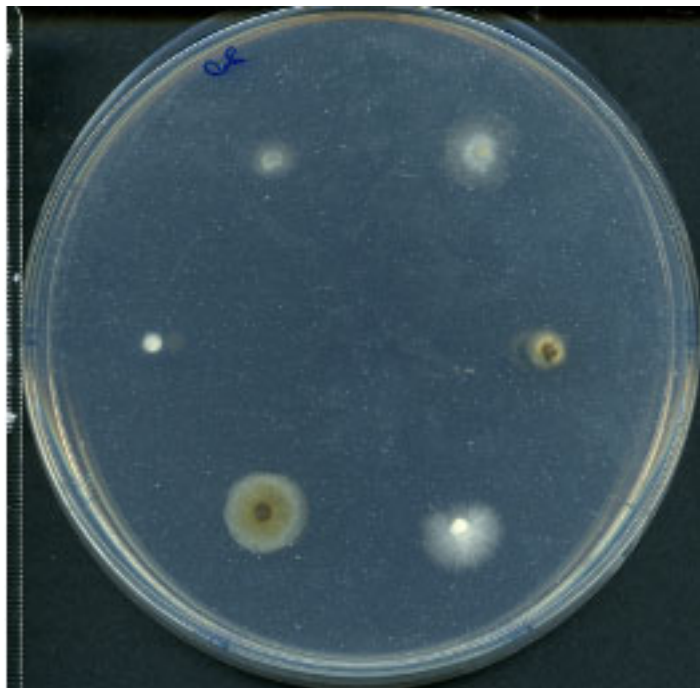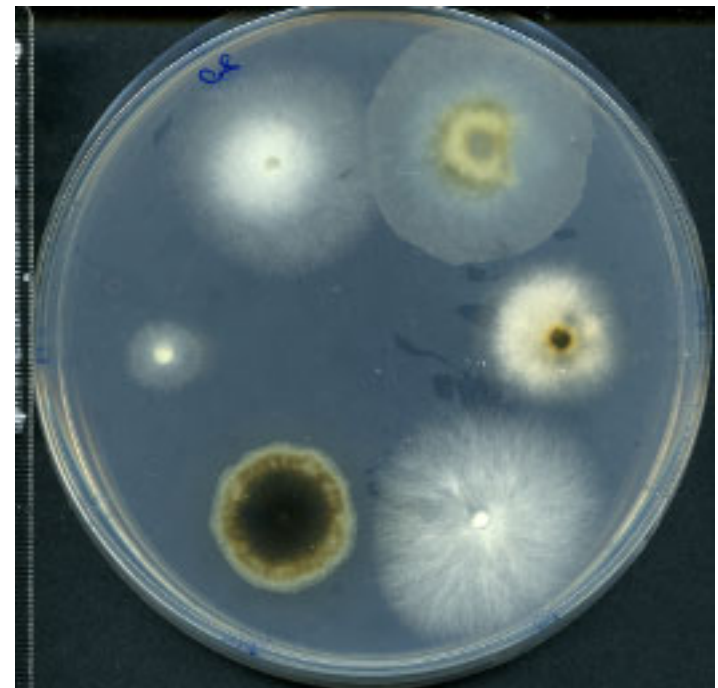

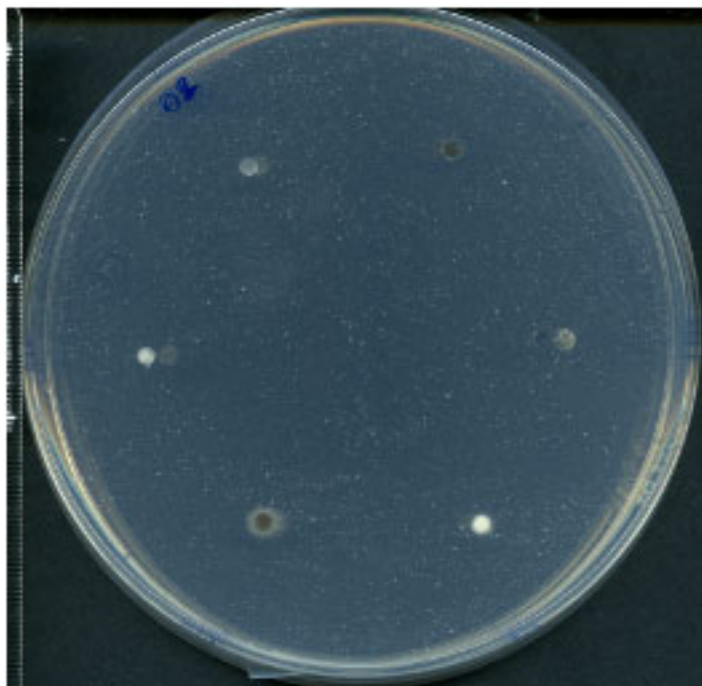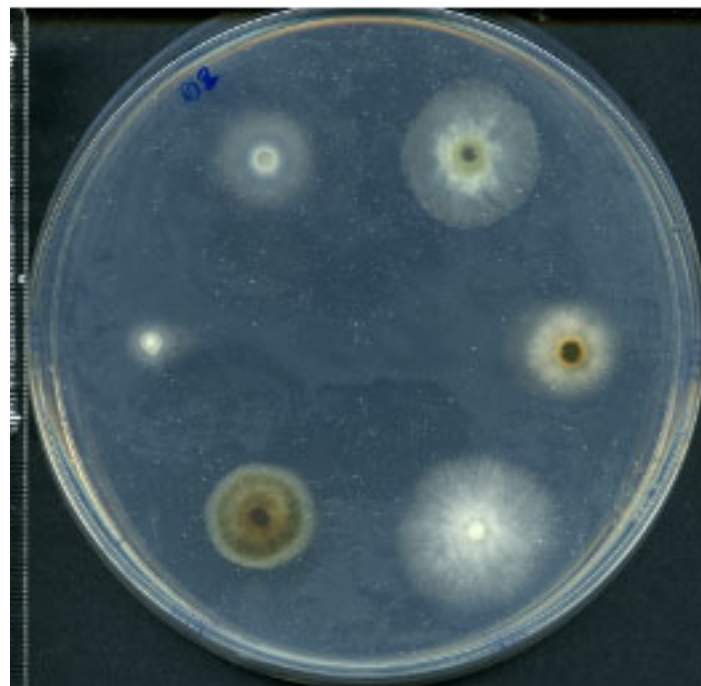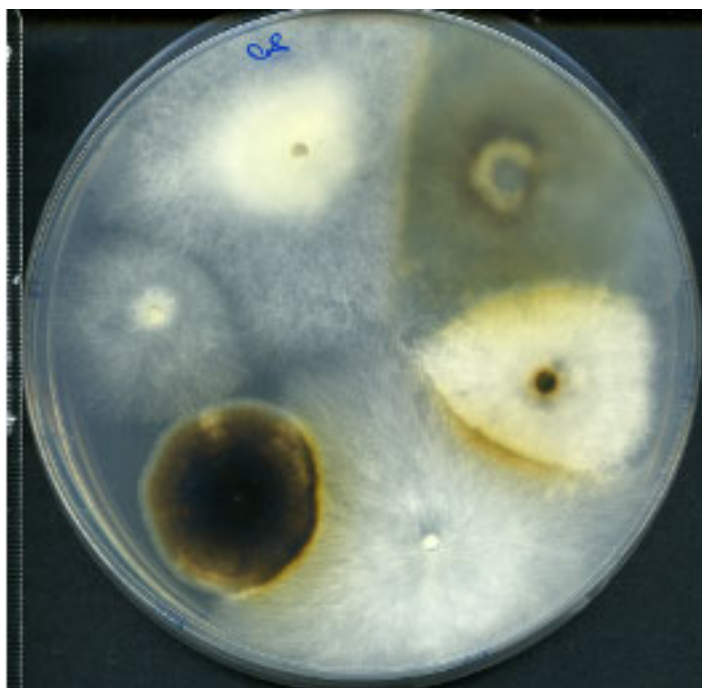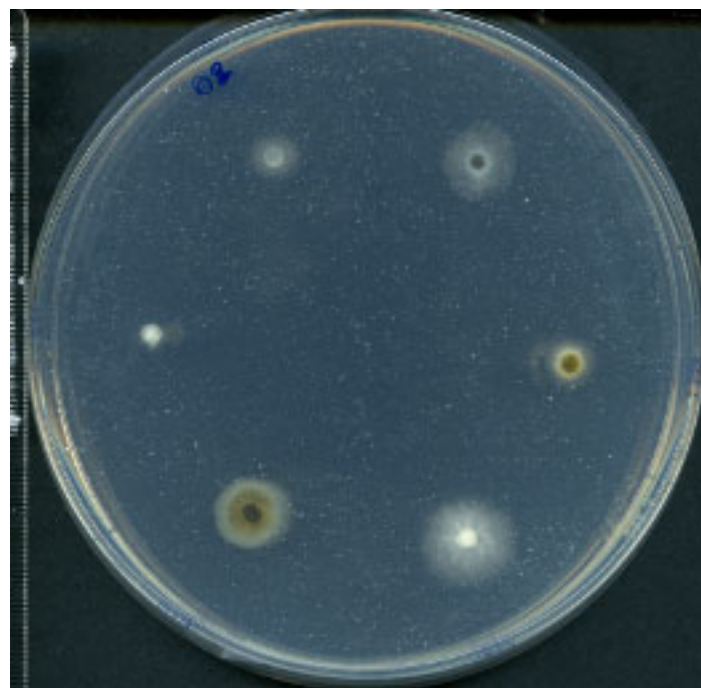

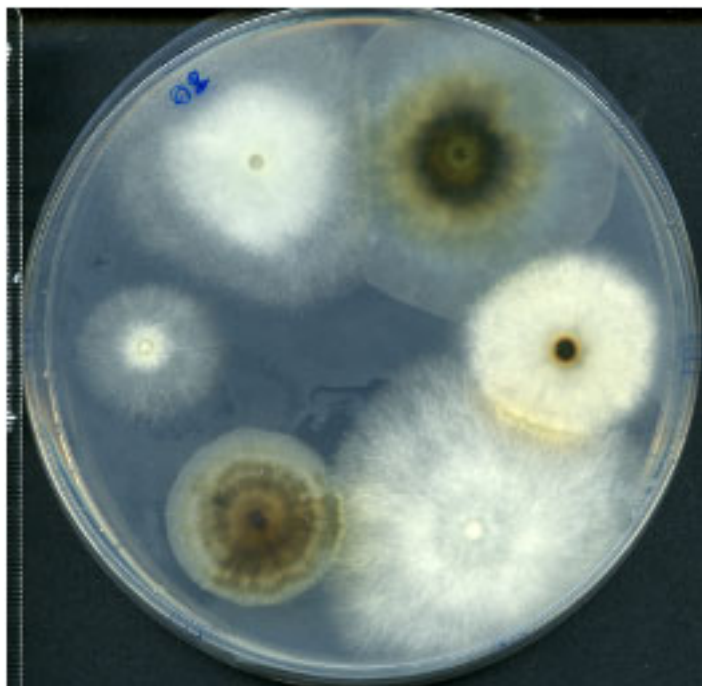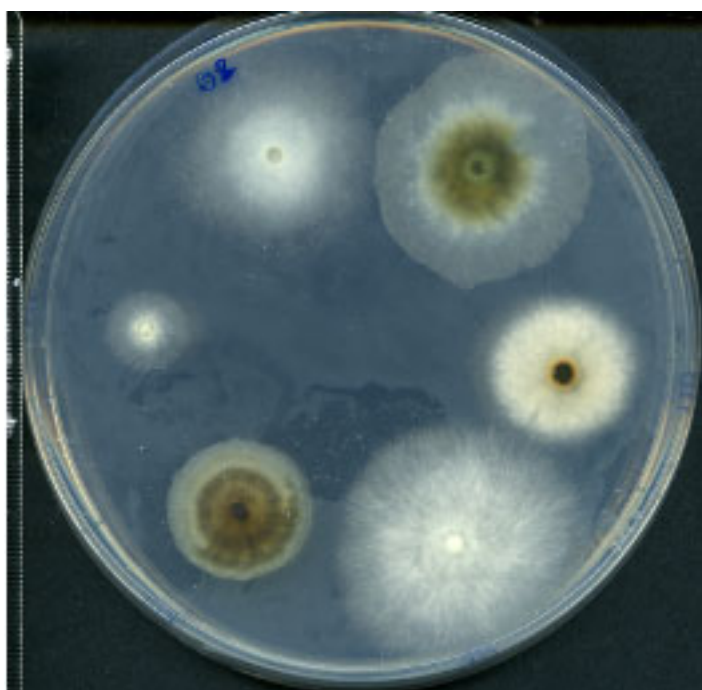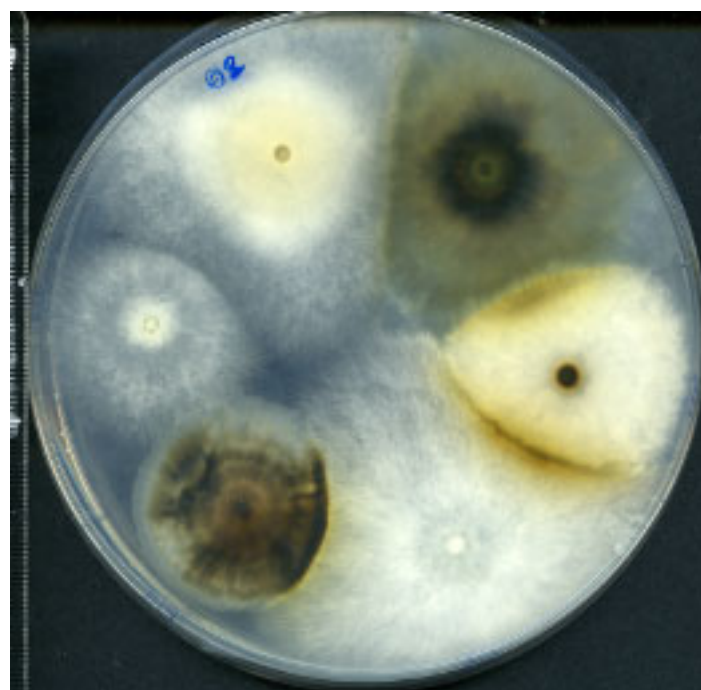

Supplement: Supplementary file 2 [file Data_Sheet_2.zip › Raw images_PDA growth Assays.pdf]
